# Supplementary material for: Cross-sectional surveys of the amount of sugar, energy and caffeine in sugar-sweetened drinks marketed and consumed as energy drinks in the UK between 2015 and 2017: monitoring reformulation progress
Source: BMJ Open. 2017 Dec 14;7(12):e018136. doi: 10.1136/bmjopen-2017-018136 (PMC5736046; doi:10.1136/bmjopen-2017-018136)
Supplement: Supplementary file 1 [file bmjopen-2017-018136supp001.pdf]

NA - product did not exist in 2015, so caffeine data collection was not applicable
NC - not collected and caffeine label may have not been available
NL - no caffeine warning label

Brand Name	Product Name	Pack Size (ml)	Energy (kcal) per 100ml 2015	Energy (kcal) per 100ml 2017	Sugars (g) per 100ml 2015	Sugars (g) per 100ml 2017	Caffeine (mg) per 100ml 2015	Caffeine (mg) per 100ml 2017
PepsiCo	Tropicana Energy Mango and Guava with Passionfruit 150ml	150	53		10.9		NC	NA
PepsiCo	Tropicana Energy Pineapple, Mango and Banana 150ml	150	49		9.8		NC	NA
Tesco	Tesco Blue Spark 6 x 250ml	250	44	24	9.8	4.9	32.0	30.0
Lidl	Lidl Freeway Up Tropical Juiced Energy Drink 250ml	250	46	36	10.5	8.8	NC	32.0
Lidl	Lidl Freeway Up Berry Juiced Energy Drink 250ml	250		36		8.8	NA	32.0
Asda	Asda Chosen by You Original Blue Charge 250ml	250	48	37	10.7	7.7	NC	30.0
Asda	Asda Chosen by You Chai Spice Flavour Blue Charge 250ml	250	43		9.6		30.0	NA
Lucozade	Lucozade Energy Pink Lemonade 250ml	250		40		6.8	NA	NL
Cott	Emerge Energy Drink Original 250ml	250	42	42	9.8	9.3	30.0	30.0
Scheckter's	Scheckter's Organic Energy 250ml	250	47	43	10.8	10	NC	32.0
Lidl	Lidl Freeway Up Lime Energy Drink 250ml	250		44		10.9	NA	32.0
Lidl	Lidl Freeway Up Classic Stimulation Drink 250ml	250		44		9.9	NA	30.0
Red Bull	Red Bull Energy Drink The Orange edition 250ml	250		45		11	NA	32.0
V	V Guarana Energy Drink 250ml	250	45	45	11.2	11	NC	31.0
Morrisons	Morrisons Source Orange and Passion Fruit Energy Drink 250ml	250	46	46	10.6	10.6	30.0	30.0
Red Bull	Red Bull Energy Drink 250ml	250	46	46	11.0	11	NC	32.0
Dico	MTV UP! Energy Drink Classic 250ml	250		46		11	NA	32.0
Red Bull	Red Bull Energy Drink The Tropical Edition 250ml	250		46		11	NA	32.0
Hergesteilt	Seagull Energy Drink 250ml	250		46		11	NA	32.0
Boost	Boost Energy Original 250ml	250	47	47	10.6	10.6	NC	30.0
Sun Mark	Bulldog Power Energy Drink 250ml	250		49		11	NA	31.5
Lucozade	Lucozade Energy Orange 250ml	250	62	62	12.5	13	NC	NL
Sainsbury's	Sainsbury's Blue Bolt Cherry & Blackcurrant Energy Drink 250ml	250	48		10.9		30.0	NA
Sainsbury's	Sainsbury's Blue Bolt Energy Drink 250ml	250	49		10.9		NC	NA
Sainsbury's	Sainsbury's Blue Bolt Mango & Passion Fruit Energy Drink 250ml	250	48		10.6		NC	NA
Asda	Asda Chosen by You Orange Twist Blue Charge Energy Drink 250ml	250	46		10.4		NC	NA
Waitrose	Waitrose Love Life Energy Release Drink 250ml	250	47		10.4		NC	NA
Big Up	Big Up Power Fruit Punch Energy Drink 250ml	250	52		12.2		30.0	NA
Big Up	Big Up Power Wild Ginger Energy Drink 250ml	250	51		12.1		30.0	NA
SoBe	SoBe Pure Rush Energy Drink Appleberry Burst 250ml	250	54		12.0		NC	NA
SoBe	SoBe Pure Rush Energy Drink Tropical Twist 250ml	250	54		12.0		NC	NA
Black	Black Mojito Energy Drink 250ml	250	47		11.3		NC	NA
Tiger	Tiger Energy Drink 250ml	250	46		10.9		NC	NA
Black	Black Energy Drink 250ml	250	46		10.8		NC	NA
Singh is King	Singh is King Energy Drink India's Energy Drink 250ml	250	48		10.8		30.0	NA
KX	KX Energy Stimulation Drink 250ml	250	44		10.1		32.0	NA
KX	KX Red Berry Stimulation Drink 250ml	250	42		10.1		NC	NA
Relentless	Relentless Origin Energy Drink 250ml	250	43		10.1		NC	NA
EQ8	EQ8 Natural Energy Drink Orange & Passion Fruit 250ml	250	40		9.3		NC	NA
EQ8	EQ8 Natural Energy Drink Cranberry & Apple 250ml	250	29		6.3		NC	NA
Organiq	Organiq Natural Energy Drink 269ml	269	42		5.6		32.0	NA
Atlantic Multipower	Multipower Red Kick 330ml	330	17	17	3	3	32.0	32.0
Little Miracles	Little Miracles Organic Energiser Lemongrass, Orange, Ginger, Ginseng, Agave	330		23		5.1	NA	NL
Little Miracles	Organic Energy Green Tea, Ginseng, Pomegranate, Acai, Acave 330ml	330	26	25	6.2	5.9	NL	NL
Little Miracles	Organic Energy White Tea, Ginseng, Cherry, Acai, Agave 330ml	330	27	26	6.3	6.1	NL	NL
Effect	Effect the Mental Energizer 330ml	330	45		10.7		32.0	NA
Little BigShot	Little BigShot Energy Mixed Berry 330ml - without caffeine!	330	36		9.0		0.0	NA
Little Miracles	Organic Energy Black Tea, Ginseng, Peach, Acai, Agave 330ml	330	27		6.6		NL	NA
Red Bull	Red Bull Energy Drink 355ml	355	46	45	11.0	11	NC	32.0
Innocent	Innocent Super Smoothie Energise 360ml	360	62	60	14.0	10	NL	NL
Powerade	Powerade Energy Sparkling Berry 375ml	375	44		10.5		NC	NA
Lucozade	Lucozade Energy Tropical Fusion Pineapple & Kiwi Flavour 6 x 380ml	380		35		4.3	NA	NL
Lucozade	Lucozade Energy Pink Lemonade 380ml	380	57	40	14.0	6.8	NC	NL
Lucozade	Lucozade Energy Caribbean Crush 380ml	380	57	40	14.0	6.5	NC	NL
Lucozade	Lucozade Energy Orange 380ml	380	62	62	12.5	13	NC	NL
Lucozade	Lucozade Energy Original 380ml	380	70	70	8.7	8.7	NC	NL
Lucozade	Lucozade Energy Apple 380ml	380	68		13.4		NC	NA
Lucozade	Lucozade Energy Tropical 380ml	380	70		13.0		NC	NA
Lucozade	Lucozade Energy Limited Edition Melonade 380ml	380	62		12.4		NC	NA
Lucozade	Lucozade Energy Cherry 380ml	380	61		12.3		NC	NA
Lucozade	Lucozade Energy Blackcurrant 380ml	380	61		12.2		NC	NA
Red Bull	Red Bull Energy Drink 473ml	473	46	45	11.0	11	32.0	32.0
No Fear	No Fear Extreme Energy 485ml	485	46		10.1		NC	NA
Monster	Monster Rehab Tea + Still Lemonade + Energy 500ml	500	10	10	2.1	2.1	NC	32.0
Relentless	Relentless Energy Drink Cherry 500ml	500		31		7.5	NA	32.0
Monster	Monster Khaos Energy + Juice 500ml	500	34	34	7.8	7.8	32.0	32.0
Lucozade	Lucozade Energy Tropical Fusion Pineapple & Kiwi Flavour 500ml	500		35		4.3	NA	NL
Monster	Monster Ripper Energy + Juice 500ml	500	47	37	10.6	8.4	NC	32.0
Monster	Monster Juiced Energy + Juice 500ml	500		37		8.4	NA	32.0
Monster	Monster Punch Energy 500ml	500		39		9	NA	32.0
Lucozade	Lucozade Energy Pink Lemonade 500ml	500	57	40	14.0	6.8	NL	NL
Lucozade	Lucozade Energy Caribbean Crush 500ml	500	57	40	14.0	6.5	NL	NL
Cott	Emerge Energy Drink Original 500ml	500	42	42	9.8	9.3	NC	30.0
Monster	Monster Energy The Doctor 500ml	500		44		10	NA	32.0
Relentless	Relentless Origin Energy Drink 500ml	500	43	45	10.1	11	NC	32.0
Relentless	Relentless Energy Drink Apple Kiwi 500ml	500		46		11	NA	32.0
KX	KX Energy Stimulation Drink 500ml	500	44	46	10.1	10.5	NC	30.0
Monster	Monster Energy 500ml	500	48	47	11.0	11	32.0	32.0
Boost	Boost Energy Original 500ml	500	47	47	10.9	10.6	NC	30.0
Relentless	Relentless Lemon Ice Energy Drink 500ml	500	48	48	11.6	12	NC	32.0
Sun Mark	Bulldog Power Energy Drink 500ml	500		48		11.3	NA	31.5
Monster	Monster Energy Assault 500ml	500		48		11	NA	32.0
Rockstar	Rockstar Energy Drink 500ml	500		51		12	NA	32.0
Lidl	Lidl Freeway Up Colossus Energy Drink 500ml	500		52		12.8	NA	32.0
Rockstar	Rockstar Xdurance Performance Energy Blueberry, Pomegranate and Acai Flavour 500ml	500	57	57	13.8	14	32.0	32.0

Brand Name	Product Name	Pack Size (ml)	Energy (kcal) per 100ml 2015	Energy (kcal) per 100ml 2017	Sugars (g) per 100ml 2015	Sugars (g) per 100ml 2017	Caffeine (mg) per 100ml 2015	Caffeine (mg) per 100ml 2017
Relentless	Relentless Energy Drink Passion Punch 500ml	500		58		14	NA	32.0
Rockstar	Rockstar Super Sours Energy Drink Bubbleburst 500ml	500	59	59	13.8	14	32.0	32.0
Lucozade	Lucozade Energy Orange 500ml	500	62	62	12.5	13	NC	NL
Lucozade	Lucozade Energy The Brazilian Mango Mandarin 500ml	500	62	62	12.4	12	NC	NL
Rockstar	Rockstar Juiced Energy + Juice Mango Orange Passion Fruit Flavour 500ml	500	66	66	15.0	15	32.0	32.0
Rockstar	Rockstar Punched Energy + Guava Tropical Guava Flavour 500ml	500	67	67	15.6	16	32.0	32.0
Sainsbury's	Sainsbury's Blue Bolt Cherry & Blackcurrant Energy Drink 500ml	500	48		10.9		NC	NA
Sainsbury's	Sainsbury's Blue Bolt Energy Drink 500ml	500	49		10.9		NC	NA
Rockstar	Rockstar Punched Energy + Punch Fruit Punch Flavour 500ml	500	64		15.2		32.0	NA
Rockstar	Rockstar Super Sours Energy Drink Green Apple 500ml	500	59		13.8		NC	NA
Rockstar	Rockstar Xdurance Performance Energy Orange Flavour 500ml	500	55		13.5		NC	NA
Lucozade	Lucozade Energy Blackcurrant 500ml	500	67		13.3		NC	NA
PepsiCo	Mountain Dew 500ml	500	48		13.0		18.0	NA
Lucozade	Lucozade Energy Limited Edition Melonade 500ml	500	62		12.4		NC	NA
Lucozade	Lucozade Energy Cherry 500ml	500	61		12.3		NC	NA
Rockstar	Rockstar Recovery Energy + Hydration Non-Carbonated Lemonade with Added Juice 500ml	500	52		12.1		NC	NA
Black	Black Energy Drink Pet 500ml	500	46		10.8		32.0	NA
Relentless	Relentless Tropical Juiced Energy Drink 500ml	500	44		10.7		NC	NA
Relentless	Relentless Berry Juiced Energy Drink 500ml	500	44		10.6		NC	NA
KX	KX Special Edition Jason Plato Energy Stimulation Drink 500ml	500	46		10.5		NC	NA
Powerade	Powerade Energy Sparkling Berry 500ml	500	44		10.5		NC	NA
Powerade	Powerade Energy Sparkling Orange 500ml	500	45		10.5		NC	NA
Relentless	Relentless Orange Energy Drink 500ml	500	43		10.2		NC	NA
Monster	Monster Energy Export 500ml	500	40		9.0		NC	NA
Lucozade	Lucozade Energy Original 500ml	500	70		8.7		NC	NA
Monster	Monster Rehab Tea Orangeade Energy 500ml	500	11		2.1		NC	NA
Monster	Monster Rehab Green Tea Energy 500ml	500	10		1.9		NC	NA
Morrisons	Morrisons Original Source Energy Drink 1 Litre	1000	44	33	10.3	7.3	30.0	30.0
Lucozade	Lucozade Energy Tropical Fusion Pineapple & Kiwi Flavour 1L	1000		35		4.3	NA	NL
Asda	Asda Chosen By You Original Blue Charge 1L	1000		37		7.7	NA	30.0
Lucozade	Lucozade Energy Pink Lemonade 1L	1000	57	40	14.0	6.8	NC	NL
Lucozade	Lucozade Energy Caribbean Crush litre	1000	57	40	13.5	6.5	NC	NL
Lucozade	Lucozade Energy The Brazilian Mango Mandarin 1L	1000		62		12	NA	NL
Lucozade	Lucozade Energy Original 1L	1000	70	70	8.7	8.7	NC	NL
Sainsbury's	Sainsbury's Orange Energy Drink 1L	1000	67		15.9		NC	NA
Lucozade	Lucozade Energy Limited Edition Melonade 1L	1000	62		12.4		NC	NA
Asda	Asda Chosen by You Mixed Fruit Blue Charge Energy Drink 1L	1000	46		10.4		NC	NA
