# Supplementary material for: Cross-sectional surveys of the amount of sugar, energy and caffeine in sugar-sweetened drinks marketed and consumed as energy drinks in the UK between 2015 and 2017: monitoring reformulation progress
Source: BMJ Open. 2017 Dec 14;7(12):e018136. doi: 10.1136/bmjopen-2017-018136 (PMC5736046; doi:10.1136/bmjopen-2017-018136)
Supplement: Author's manuscript [file bmjopen-2017-018136.draft_revisions.pdf]

BMJ Open is committed to open peer review. As part of this commitment we make the peer review history of every article we publish publicly available.

When an article is published we post the peer reviewers' comments and the authors' responses online. We also post the versions of the paper that were used during peer review. These are the versions that the peer review comments apply to.

The versions of the paper that follow are the versions that were submitted during the peer review process. They are not the versions of record or the final published versions. They should not be cited or distributed as the published version of this manuscript.

BMJ Open is an open access journal and the full, final, typeset and author-corrected version of record of the manuscript is available on our site with no access controls, subscription charges or pay-per-view fees (http://bmjopen.bmj.com).

If you have any questions on BMJ Open's open peer review process please email editorial.bmjopen@bmj.com

BMJ Open

Cross-sectional surveys of the amount of sugars, calories and caffeine in energy drinks in the UK between 2015 and 2017

Journal:	*BMJ Open*
Manuscript ID	bmjopen-2017-018136
Article Type:	Research
Date Submitted by the Author:	08-Jun-2017
Complete List of Authors:	Hashem, Kawther; Wolfson Institute of Preventive Medicine, Queen Mary University of London<br>He, Feng; Queen Mary University of London, Wolfson Institute of Preventive Medicine<br>MacGregor, Graham; Barts and the London School of Medicine and Dentistry, Wolfson Institute of Preventive Medicine
**Primary Subject Heading**:	Public health
Secondary Subject Heading:	Health policy, Public health
Keywords:	sugar, calories, energy drinks, caffiene

SCHOLARONE™
Manuscripts

Title: Cross-sectional surveys of the amount of sugars, calories and caffeine in energy drinks in the UK between 2015 and 2017

Kawther M Hashem, Feng J He, Graham A MacGregor

Wolfson Institute of Preventive Medicine, Barts and The London School of Medicine & Dentistry, Queen Mary University of London, UK

Running title: Sugars content of energy drinks

Correspondence to:

Kawther Hashem

Wolfson Institute of Preventive Medicine, Barts and The London School of Medicine & Dentistry,

Queen Mary University of London, Charterhouse Square, London, EC1M 6BQ.

Tel: 44 (0)20 7882 5941

Fax: 44 (0)20 7882 6270

E-mail: k.hashem@qmul.ac.uk

Keywords: sugars, calories, caffeine, energy drinks

For peer review only - http://bmjopen.bmj.com/site/about/guidelines.xhtml

ABSTRACT

Objectives: To investigate the free sugars and calorie content of energy drinks available in the main UK supermarkets.

Study design: We carried out a cross-sectional survey in 2015 (102) and 2017 (65) of energy drinks available in UK.

Methods: The free sugars (sugars g/100ml), calorie (kcals/100ml), caffeine (mg/100ml) and serving size was collected from product packaging and nutrient information panels of energy drinks available in nine main UK supermarkets and three beauty and health food retailers.

Results: Energy drinks consistently surveyed (n = 30) showed significant reduction of sugar and calorie content by 10% (p=0·014) and 6% (p=0·007) respectively, 53% of products have reduced their sugar content. Despite this, sugar content was still high. In 2017, average sugar and calorie content in energy drinks was (±SD) 9·7±3·0g/100ml and 43±13 kcal/100ml respectively. There was a large variation in sugar and calorie content between different energy drinks ranging from 2·1 to 16·0g/100ml and 10 to 70 kcal/100ml. Among the 65 products surveyed, 74% exceeded the maximum daily recommendation for sugar intake for a child. The average caffeine content in energy drinks was 126·3±39·2 mg/serving among the 41 products labelled.

Conclusions: There has been a reduction in sugar content of energy drinks in the UK since 2015. However, sugar content remains high. Sugar content of energy drinks is of concern, with a typical serving exceeding an adult's maximum daily recommendation. The benefits of reducing sugar will be large in reducing both sugar and calorie consumption and therefore a reduction in obesity, type 2 diabetes and dental caries.

Strengths and limitations of this study

- This paper for the first time investigates the sugars, calories and caffeine content of energy drinks sold in the UK supermarkets.

For peer review only - http://bmjopen.bmj.com/site/about/guidelines.xhtml

- This paper shows that the sugar content of energy drinks is high and there is a wide variation in the sugar content of energy drinks.
- These findings demonstrate that the amount of free sugars added to energy drinks could be reduced without technical issues.
- The study was based on the sugars content data provided on energy drinks packaging labels in store; hence we relied on the accuracy of the data provided on the label.

INTRODUCTION

Energy drinks are typically non-alcoholic drinks containing caffeine and sugar in combination with other ingredients known to have stimulant properties and are distinct from sports drinks and vitamin waters. Worldwide consumption of energy drinks has been increasing dramatically in recent years. The $50 billion global energy drinks market is projected to grow at an annual rate of 3·5% between 2015 and 2020, even though the soft drinks market is declining overall.[1] The increase in the popularity of energy drinks has raised concerns among the scientific community, governments and the general public about the health effects of these products, particularly among children and adolescents.[2-4]

In 2011, a study by the European Food Safety Authority (EFSA) on consumption data of energy drinks in 16 countries of the European Union found that 68% of adolescents (aged 10–18 years old), 30% of adults, and 18% of children (<10 years old) consumed energy drinks.[2] Another study by EFSA found that young people in the UK consumed more energy drinks than their counterparts across the other EU countries (3.1 litres per person per month, compared with 2 litres).[5] Market research reports potential increase in purchase of energy drinks among 10-14 year olds.[6]

The rise in their popularity and their high sugar content suggests it is contributing to obesity, type 2 diabetes and dental caries,[7-15] all are major public health problems in the UK,[16-23] with significant costs.[24]

In July 2015, the Scientific Advisory Committee on Nutrition (SACN) recommended average intake, across the UK population, of free sugars should not exceed 5% of total energy

For peer review only

For peer review only - http://bmjopen.bmj.com/site/about/guidelines.xhtml

intake.[25] This is in line with the World Health Organisation's new guidelines on free sugars intake. [15, 26] 'Free sugars' includes all monosaccharides and disaccharides added to foods by the manufacturer, cook or consumer, plus sugars naturally present in honey, syrups and unsweetened fruit juices. Under this definition lactose (milk sugar) when naturally present in milk and milk products and sugars contained within the cellular structure of foods (particularly fruits and vegetables) are excluded. Furthermore, SACN advised that consumption of sugars-sweetened drinks, including energy drinks, should be minimised in children and adults.

Current average intakes of free sugars exceed recommendations in all age groups. The average sugars intake in adults was 59g per day and contributes to 12·1% of energy intake. Children have a higher sugars intake. The average sugars intake was 61 and 74g per day in 4-10 and 11-18 year olds respectively, about three times the new recommendation in 11 to 18-year olds. [27] This is likely to be an underestimate of how much free sugars are consumed[28] because underreporting is highly prevalent in these types of surveys. [29-32]

Soft drinks are the main contributor of free sugars intake in children (4-10 years), teenagers (11-18 years) and the second main contributor in adults (18-64 years), contributing to 30%, 40% and 25% of free sugars intake respectively.[27] Within soft drinks, energy drinks are an important contributor of free sugars intake. Sales of energy drinks in the UK have increased by 155% between 2006 and 2014, from 235 to 600 million litres, an average per capita consumption of 9·4 litres in 2014, and a total value of £1·48 million.[33] The leading brands' shares in the UK energy drinks market, by value in 2013/14, were Red Bull (25%), own-label (12%), Monster (10%), Relentless (6%), Rockstar (5%), Mountain Dew (2%), Boost (2%) and Emerge (2%).[6]

In the UK, the government plans in 2018 to implement its own levy on sugary drinks with more than 5g of sugar per 100ml, with a higher rate for drinks (including energy drinks) with more than 8g per 100ml. The impact of these types of levies on sales of energy drinks remains to be seen.[34]

While the potential impact of energy drinks in terms of their caffeine have been discussed in recent years[2, 35] little research has looked at the levels of sugars, calories and caffeine in

For peer review only

For peer review only - http://bmjopen.bmj.com/site/about/guidelines.xhtml

energy drinks which can impact obesity, type 2 diabetes and dental caries. Considering this, we carried two cross-sectional surveys to evaluate the sugars, calorie and caffeine content of energy drinks sold in the UK between 2015 and 2017, report the variability in sugar level, assess the sugar content in relation to the UK's new daily recommendation for sugar intake and assess the number of products that will be taxed based on the criteria suggested in the Soft Drinks Industry Levy.

METHODS

Data Collection

The data was collected from product packaging and nutrient information panels in January 2015 and January 2017. The survey was designed as a comprehensive survey of all energy drinks available in a snapshot in time, using one large outlet per each of the nine main grocery retailers, three beauty and health food retailers and one convenience store.

For each energy drink, the data collected included the company name, product name, pack weight, serving size, sugars (g), energy (kcal) and caffeine (mg) per 100ml and sugars, energy and caffeine per serving. All data was double checked after entry, and a further 5% of entries were checked against the original source in a random selection of products. This study used the same study design and procedures as reported in our previous work.[36]

Inclusion/exclusion criteria: Data were collected from each of the major UK supermarkets (Aldi, Asda, Lidl, Marks and Spencer, Morrisons, Sainsbury's, Tesco, The Co-operative and Waitrose) and three beauty, health food retailers (Boots, Superdrug and Holland and Barrett) and a Costcutter store to represent the amount of free sugars in energy drinks in the UK. We included supermarket own brand and branded products. Energy drinks products with sugars and calories information labeled were included. As the study focus is on product reformulation, products labelled called 'zero' or 'light' or 'no added sugar' etc. were excluded.

Energy drinks were defined as any drinks products with 'energy', 'energise', 'energising', 'caffeine' and 'stimulation' in the product name. We also excluded isotonic or sports drinks, which consist primarily of carbohydrates and electrolytes and are intended to help athletes

For peer review only - http://bmjopen.bmj.com/site/about/guidelines.xhtml

rehydrate after exercise.

The caffeine content of a small selection of products were collected in 2015 (n =11) so comparison over the two surveys of caffeine content were not made, as the sample size from 2015 was too small.

Serving size: The serving size was calculated from per 100ml nutrition information provided on packaging. Any products with up to 500ml can or bottle size was considered as one serving, regardless of what is stated on the packaging as a serving size e.g. often a 500ml bottle is split into two serving but we consider that most consumers consume these drinks as one serving. In 1 litre products, the serving size was standardised to 250ml. If a 250ml can of the same product exists then, the litre product was excluded.

High, Medium and low criteria for sugars content: The free sugars content will be compared to the UK front of pack colour-coded labelling for drinks. Portion size criteria applies to portion/serving sizes greater than 150ml. Colour coding is based on the following front of pack colour-coded nutrition labelling criteria (Sugars – Red/High >13·5g/portion or >11·25g/100ml, Amber/Medium >2·5 to ≤11·25/100ml, Green/Low ≤2·25g/100ml).[37]

Per 100ml verses per serving: Some brands sell the same formulation in different serving sizes. Therefore, the 100ml data only included an example of one formulation regardless of the different serving sizes, while the serving data included all the different serving sizes available, even if it is the same formulation.

Statistical analysis

Comparison among products within each survey

Independent-Samples Mann-Whitney U Test was used to compare the levels of sugars between supermarket own brand and branded products.

Comparison of the same products between the two years

For peer review only - http://bmjopen.bmj.com/site/about/guidelines.xhtml

For the purpose of this comparison, only the products with data available in both surveys were included in the analysis. Related-Samples Wilcoxon Signed Rank Test was used to examine whether there was a significant change in the sugar and calorie content of energy drinks between 2015 and 2017.

Data are reported as mean, SD, range as indicated. Significance in all tests carried out was deemed significant as being p<0·05. All data were analysed using SPSS Version 22.
In addition to the above analyses, the sugar content was compared to the UK's new recommendation for sugar intake for adults (30g/day) and 7-10 year old children (24g/day) and to the colour-coded labelling for sugars content.

RESULTS

Sugar, calorie and caffeine content per 100 ml in the 2015 survey

A total of 79 energy drinks met the inclusion criteria and were included in our analysis. The average sugar content in energy drinks was (±SD) 10·8±2·8g/100ml. There was a large variation in sugars content between different energy drinks ranging from 1·9 to 15·9g/100ml.
A total of 68 products were branded and 11 were supermarket own-brand energy drinks (Table 1). There were no significant differences in sugar content between supermarket own brand and branded products (11·0±1·7 vs 10·7±3·0g/100ml, P=0·444).

The average calorie content in energy drinks was 48±13 kcal/100ml. There was a large variation in calorie content between different energy drinks ranging from 10 to 72 kcal/100ml. There were no significant differences in sugar content between supermarket own brand and branded products (48±6 vs 48±14 kcal/100ml, P=0·938).

A total of 8 energy drinks formulations (per 100ml) had caffeine content labelled on pack (there may have been more that were labelled but not all were collected in 2015). The average caffeine content in energy drinks was (±SD) 30·0±4·9 mg/100ml.

Sugar, calorie and caffeine content per serving in the 2015 survey

For peer review only - http://bmjopen.bmj.com/site/about/guidelines.xhtml

A total of 102 energy drinks products met the inclusion criteria and were included in our analysis, 89 products were branded and 13 were supermarket own-brand energy drinks (Table 2). The serving size varied from 150 to 500ml. Among the 102 products 39% were sold in 500ml cans/bottles.

The average sugar content in energy drinks was high (±SD) 40·8±17.6g/serving. Among the 102 products 96% would receive a 'red' (high) label for sugars per serving (>13·5g/serving). There was a large variation in sugars content between different energy drinks ranging from 9·5 to 78·0g/serving.

The serving size was significantly larger in branded verses supermarket own brand products (P=0·004), as a result the branded contained on average higher levels of sugars compared to supermarket products per serving (42·2±17·9 vs 31·6±10·8g/serving, P=0·036).

On average, a serving of energy drink (40·8g/serving) contains more than the entire maximum daily recommendation for sugar intake in the UK for an adult (30g).

Among the 102 products 62% exceeded the maximum UK's recommendation for sugar intake per serving for an adult (30g/d). Additionally, 87% of the products exceeded the maximum daily recommendation for sugar intake for a child aged 7-10 years (24g/d).

The average calorie content in energy drinks was 184±79 kcal/serving. There was a large variation in calorie content between different energy drinks ranging from 50 to 350kcal/serving. Supermarket own brand (140±48kcal/serving) contained fewer calories than branded (190±82kcal/serving) products, but this was not statistically significant (p=0·069).

The energy drink product with the highest calorie content (350 kcal) can contribute up to 17·5% of a woman's daily energy intake (2000 kcal).

A total of 11 energy drinks formulations (per serving) had caffeine content labelled on pack and were collected in 2015. The average caffeine content was 133·4±50·7 mg/serving.

Sugar, calorie and caffeine content per 100ml in the 2017 survey

A total of 48 energy drinks products met the inclusion criteria and were included in our analysis, 39 products were branded and 9 were supermarket own-brand energy drinks

For peer review only - http://bmjopen.bmj.com/site/about/guidelines.xhtml

For peer review only

(Table 1). The average sugar content in energy drinks was 9·7±3·0g/100ml, ranging from 2·1 to 16·0g/100ml. There was no significant differences in sugar content between supermarket own brand and branded products (9·1±2·3 vs 9·8±3·2g/100ml, P=0·261). The average calorie content in energy drinks was 45±13 kcal/100ml, ranging from 10 to 70 kcal/100ml. There was no significant differences in sugar content between supermarket own brand and branded products (39±8 vs 44±13 kcal/100ml, P=0·114).

A total of 36 out of 48 energy drinks formulations (per 100ml) had caffeine content labelled on pack, 27 products were branded and 9 were supermarket own-brand energy drinks. The average caffeine content in energy drinks was 31·5±0·9 mg/100ml. The caffeine content ranged from 30·0 to 32·0g/100ml. There were no significant differences in caffeine content between supermarket own brand and branded products (30·9±1·1 vs 31·7±0·7 mg/100ml, P=0·067). Some of products with labelled caffeine content also stated the following *'high caffeine content – not recommended for children or pregnant or breast-feeding women. Please consume responsibly',* which is an important warning to have on pack. However, these products can be easily purchased and consumed by children.

Compared to proposed Soft Drink Industry Levy

A total of 35 products (73%) would be taxed at the higher sugar levy rate (>8g /100ml), 9 products (19%) at the lower rate (8-5g /100ml) and 5 products (10%) won't be taxed (<5g /100ml).

Sugar, calorie and caffeine content per serving in the 2017 survey

A total of 65 energy drinks products met the inclusion criteria and were included in our analysis, 9 products were branded and 56 were supermarket own-brand energy drinks (Table 2).

The serving size varied from 250 to 500ml. Among the 65 products 45% were sold in 500ml cans or bottles. The average sugar content in energy drinks was high 37·7±18·3g/serving. Among the 65 products, 61 products (94%) would receive a 'red' (high) label for sugars per serving (>13·5g/serving). There was a large variation in sugars content between different energy drinks ranging from 9·9 to 80·0g/serving.

For peer review only - http://bmjopen.bmj.com/site/about/guidelines.xhtml

The serving size was significantly larger in branded verses supermarket own brand products (P=0·002), thus supermarket own brand contained on average lower levels of sugars compared to branded products per serving (26·3±14·9 vs 39·5±18·3g/serving, P=0·024) (Figure 1).

The average calorie content in energy drinks was 173±75kcal/serving. There was a large variation in calorie content between different energy drinks ranging from 50 to 335kcal/serving. Supermarket own brand (112±58kcal/serving) contained significantly fewer calories than branded (183±73kcal/serving) products, (p=0·005) (Figure 2). The energy drink product with the highest calorie content (335 kcal) can contribute up to 17% of a woman's daily energy intake (2000 kcal).

A total of 41 out of 65 energy drinks products provided caffeine content per serving or we could calculate caffeine content per serving, 9 products were branded and 32 were supermarket own-brand energy drinks. The average caffeine content in energy drinks was 126·3±39·2 mg/serving. There was a large variation in caffeine content between different energy drinks ranging from 75·0 to 160·0 mg/serving. There was a significant difference in caffeine content between supermarket own brand and branded products (86·1±27·8 vs 137·6±34·3 mg/serving, P=0·001).

The total number of products with labelled caffeine content here is different to the number of products included per 100ml analysis because the 100ml data only included an example of one formulation regardless of the different serving sizes, while the serving data included all the different serving sizes available, even if it is the same formulation. The caffeine content per serving differed between products with the same formulation; therefore, it was necessary to include all the products regardless if they are the same formulation.

Comparing to the maximum daily recommendation for sugar intake

On average, a serving of energy drink (37·7g/serving) contains more than the entire maximum daily recommendation for sugar intake in the UK for an adult (30g).

Among the 65 products, 36 (55%) exceeded the maximum UK's recommendation for sugar intake per serving for an adult (30g/d). Additionally, 48 products (74%) exceeded the

For peer review only - http://bmjopen.bmj.com/site/about/guidelines.xhtml

maximum daily recommendation for sugar intake for a child aged 7-10 years (24g/d).

Comparison of the same products between the two years

The overall number of branded and supermarket own brand energy drinks has decreased from 2015 to 2017 (102 vs 65 products).

Per 100ml

There were 30 products (26 branded and 4 supermarkets own brand) surveyed repeatedly over the 2 years. The average sugars content per 100 ml for these 30 products were 10·7±3·1g in 2015 and 9·6±3·4g in 2017. This represents a reduction of 10% from 2015 to 2017 (p=0·014). The average calorie content per 100ml for these 31 products were 47±14 kcal in 2015 and 44±15 kcal in 2017, representing a 6% reduction (p=0·007). A total of 16 products (53%) have reduced, 9 have stayed the same and 5 have increased in sugar content.

Per serving

The average sugars content per serving for these 41 products were 43·5±18·5 g in 2015 and 38·1±19·4 g in 2017, i.e. a 13% reduction (p=0·003). The average calorie content per serving for these 41 products were 193±79 kcal in 2015 and 178±82 kcal in 2017, i.e. a 8% reduction (p=0·001).

Note that these averages are slightly different from those when all products were included in each year and this trend analysis reflects reductions made in the same products rather than the overall products available.

DISCUSSION

Energy drinks consistently surveyed showed significant reduction of sugar content. Despite this, sugar content was still high. In 2017, the average sugar content in energy drinks was (±SD) 9·7±3·0g/100ml. There was a large variation in sugar content between different energy drinks ranging from 2·1 to 16·0g/100ml. The average sugar content in energy drink in both surveys per serving was more than an adult's entire maximum daily recommendation for sugar intake in the UK (40·8±17·6g vs 37·7 ±18·3g/serving). Furthermore, among the 65

For peer review only - http://bmjopen.bmj.com/site/about/guidelines.xhtml

products surveyed, 74% exceeded the maximum daily recommendation for sugar intake for a child aged 7-10 years. Therefore the claim that energy drinks can be consumed as part of a 'healthy balanced diet' is misleading.[25]

Due to high sugar level, the calorie content in energy drinks is high with an average of 184±80kcal in 2015 and 173±74kcal/serving in 2017. The products with the highest sugars and calorie content could contribute up 17% of a woman's total daily energy intake. Whilst certain drinks manufacturers claim their products are a good source of energy, this is also misleading. The body generates energy from any food, such as fruits, vegetables, breads, pasta and rice and there is no need whatsoever for added sugar that are in energy drinks. The term 'energy drinks' is chosen to communicate a falsehood in disguise, i.e. that these drinks are full of energy giving nutrients rather than sugar that can contribute to weight gain, if not burnt off. Therefore, the levels of sugar and calories in energy drinks can be an important contributor to obesity, type 2 diabetes and dental caries and therefore substantial healthcare costs [38-40].

Besides, the concerns around the sugar and calorie content of energy drinks, there is the concern around the levels of caffeine in them. In 2017, average caffeine content in energy drinks was 126·3±39·2 mg/serving among the 41 products labelled, almost equivalent to two cups of coffee. There is also some evidence to suggest that the caffeine content increases sugar-sweetened beverage consumption in a free-living population.[41] It important to note that not all companies label the amount of caffeine in their energy drinks but among the companies that do, they also state a warning on pack, acknowledging the potential danger these products can possess to children. However, these products can be easily purchased and consumed by children.

Energy drinks also contribute to sugar intake in many other countries around the world,[2] showing that high levels of sugars as well as caffeine in energy drinks is a global challenge.[2, 35, 42] Besides the warning label on the back of packs in the UK, a few countries have taken other measures to regulate sales of energy drinks, mainly due to their high caffeine content. A law banning the sale of energy drinks to under-18s in Lithuania was

For peer review only - http://bmjopen.bmj.com/site/about/guidelines.xhtml

introduced in November 2014. The law applies to non-alcoholic drinks that contain at least 150mg of caffeine per litre.[43] In Sweden, sales of some types of energy drinks are restricted to pharmacies and sales to children are also banned.[2]

Aside from bans on sale of energy drinks many other countries have implemented, or are in the process of implementing, taxes and levies on high sugar food and/or drinks, including energy drinks.[44] For example, in Hungary, a 'public health tax' adopted in 2012 was applied on the salt, sugar and caffeine content of various categories of ready-to-eat foods and drinks. In Mexico, a 10% tax has been applied to sugary drinks since January 2014. In the 12 months following the introduction of this tax, an average of 4.2 litres fewer sugary drinks were purchased per person.[45] Purchases decreased by 12% by December 2014. This was coupled with a 4% rise in sales of untaxed drinks, primarily bottled water. In Dominican Republic, a 10% excise tax has been applied since September 2015, onto food and drinks with high sugar content, which includes soft drinks. Revenues contribute to a national 'Get Healthy' campaign. In the UK, the government plans in 2018 to implement its own levy on sugary drinks with more than 5g of sugar per 100ml, with a higher rate for drinks (including energy drinks) with more than 8g per 100ml. The impact of these types of levies on sales of energy drinks remains to be seen.[34]

Another option a country can adopt, to protect children and adolescents from energy drinks is implement a systematic, unobtrusive and gradual reformulation programme for manufacturers to reduce the amount of sugar and caffeine in energy drinks.[46] The reductions seen so far suggest that the UK market may be adopting this approach.

A similar successful reformulation programme has been implemented in the UK to reduce salt intake in the whole population. The key to the success of the UK salt reduction programme is setting incremental targets for each food group with a specified timeframe to be achieved using maximum and average or sales-weighted average targets [47]. Since there has been a gradual, progressive reduction in salt, the UK population has adjusted to the taste of lower salt levels. There has been no loss of sales or switching between products as a result of salt reduction, or addition of salt at the table. As this programme targets all foods, and does not rely on consumer behaviour change, it particularly benefits people from lower

For peer review only - http://bmjopen.bmj.com/site/about/guidelines.xhtml

income households who consume more unhealthy diets than people in higher income households.[48]

A similar programme for manufacturers can be implemented to reduce sugar content. This would be achieved by setting progressive targets for each food and drink category, which would allow for an incremental reduction of sugar and provide a level playing field to industry, which is vital if it is implemented on a voluntary basis. Importantly, there would be no substitution with non-caloric sweeteners, so that the taste receptors would adjust.[49, 50] Given the volume consumed,[51] even small reductions in sugar content of energy drinks would have a significant impact on sugar and calorie intake of the population. Governments can also restrict the amount of caffeine added to products. Indeed in a recent study it was shown that that removing caffeine from sugar-sweetened drinks, along with 10·3% of sugar, has the potential to reduce body weight of adults by 600g, without any change in sugar-sweetened drinks consumption.[52]

Alongside reducing the free sugars and caffeine content and governments can introduce regulation to restrict larger serving size. Recently, the Grocer has reported an increase in sales of larger cans of energy drinks on the UK market, which must be tackled to reduce intake of sugar, calories and caffeine from these products.

Our study was based on sugars, calorie and caffeine content data provided on the available energy drinks packaging labels in store on the dates of collection; hence we relied on the accuracy of the data provided on the label. Therefore, it is assumed that the manufacturers provide accurate and up to date information in line with EU Regulations. However, future studies should include free sugars and calorie content determined through laboratory analysis to achieve a better understanding of the true free sugars and calorie content, and breakdown of free sugars composition. Still, the results of this study are relevant and serve to record the sugars, calorie and caffeine content of energy drinks sold the in UK, underpinning future studies and providing evidence for the Treasury that are proposing the Soft Drink Industry Levy and the soft drink industry.

For peer review only

CONCLUSION

The full impact of the rise in popularity of energy drinks has not yet been measured, but the increase in sales,[33, 53, 54] the high amount of caffeine and free sugars in energy drinks (measured in this study), the pervasive and misleading marketing targeted at young people,[55, 56] the ease of access,[3] combined with limited regulation have created an environment where energy drinks could pose a major public health threat.

This study indicates there has been a reduction in sugar content of energy drinks in the UK since 2015. Sugars content however remains high. This research demonstrates that sugars content of energy drinks in the UK is of concern, with a typical serving (500ml) exceeding an adult's entire maximum daily recommendation of sugar intake. More can and should be done. The benefits will be very large in reducing both sugars and calorie consumption and therefore a reduction in obesity, type 2 diabetes and dental caries.

COPYRIGHT

The Corresponding Author has the right to grant on behalf of all authors and does grant on behalf of all authors, a worldwide licence to the Publishers and its licensees in perpetuity, in all forms, formats and media (whether known now or created in the future), to i) publish, reproduce, distribute, display and store the Contribution, ii) translate the Contribution into other languages, create adaptations, reprints, include within collections and create summaries, extracts and/or, abstracts of the Contribution, iii) create any other derivative work(s) based on the Contribution, iv) to exploit all subsidiary rights in the Contribution, v) the inclusion of electronic links from the Contribution to third party material where-ever it may be located; and, vi) licence any third party to do any or all of the above.

COMPETING INTERESTS

All authors have completed the ICMJE uniform disclosure form at www.icmje.org/coi_disclosure.pdf (available on request from the corresponding author) and declare: no support from any organisation for the submitted work; no financial relationships with any organisations that might have an interest in the submitted work in the previous

For peer review only - http://bmjopen.bmj.com/site/about/guidelines.xhtml

three years. KH is an employee of Consensus Action on Salt & Health (CASH), a nonprofit charitable organisation. FH is a member of CASH and its international branch World Action on Salt & Health (WASH) and does not receive any financial support from CASH or WASH. MacGregor GA is Chairman of Blood Pressure UK (BPUK), Chairman of CASH, Chairman of WASH and Chairman of Action on Sugar. BPUK, CASH , WASH and Action on Sugar are nonprofit charitable organisations. GM does not receive any financial support from any of these organisations.

CONTRUBUTORSHIP

KH conducted research; KH and FH analysed data; KH wrote the first draft of the manuscript and all authors contributed to the interpretation of the results and revision of the manuscript, and approved the final manuscript. GM had primary responsibility for final content.

TRANSPARENCY DECLARATION

The manuscript is an honest, accurate, and transparent account of the study being reported; that no important aspects of the study have been omitted; and that any discrepancies from the study as planned (and, if relevant) have been explained.

FUNDING

This research received no specific grant from any funding agency in the public, commercial or not-for-profit sectors.

ETHICS

Not applicable.

ROLE OF SPONSOR

Not applicable.

DATA SHARING STATEMENT

Extra data is available by emailing Kawther Hashem on k.hashem@qmul.ac.uk.

For peer review only - http://bmjopen.bmj.com/site/about/guidelines.xhtml

REFERENCE

1. Mordor Intelligence LLP. Global Energy Drinks Market Forecasted for 2015-2020 2016 [25 August 2016]. Available from: https://www.reportbuyer.com/product/3682281/global-energy-drinks-market-forecasted-for-2015-2020.html.
2. Breda JJ, Whiting SH, Encarnação R, et al. Energy drink consumption in Europe: A review of the risks, adverse health effects and policy options to respond. *Frontiers in Public Health*. 2014;2.
3. Visram S, Hashem, K,. Energy drinks: what's the evidence? *Food Research Collaboration* 2016.
4. European-Parliament. Schaldemose: "Energy drinks shouldn't have any kind of health claims on them" 2016 [1 September]. Available from: http://www.europarl.europa.eu/news/en/news-room/20160613STO31976/schaldemose-energy-drinks-shouldn't-have-any-kind-of-health-claims-on-them.
5. Consortium N-A. Gathering consumption data on specific consumer groups of energy drinks2013 20 March 2017. Available from: http://onlinelibrary.wiley.com/doi/10.2903/sp.efsa.2013.EN-394/pdf.
6. Mintel. Sports and Energy Drinks - UK - July 2014 2014.
7. SACN. Draft Carbohydrates and Health report Scientific consultation: 26 June to 1 September 20142014 1 December 2015. Available from: https://www.gov.uk/government/uploads/system/uploads/attachment_data/file/339771/Draft_SACN_Carbohydrates_and_Health_report_consultation.pdf.
8. Romaguera D, Norat T, Wark PA, et al. Consumption of sweet beverages and type 2 diabetes incidence in European adults: results from EPIC-InterAct. *Diabetologia*. 2013;56:1520-30.
9. de Koning L, Malik VS, Rimm EB, et al. Sugar-sweetened and artificially sweetened beverage consumption and risk of type 2 diabetes in men. *The American journal of clinical nutrition*. 2011;93:1321-7.
10. Maki KC, Phillips AK. Dietary substitutions for refined carbohydrate that show promise for reducing risk of type 2 diabetes in men and women. *J Nutr*. 2015;145:159S-63S.
11. Feinman RD, Pogozelski WK, Astrup A, et al. Dietary carbohydrate restriction as the first approach in diabetes management: Critical review and evidence base. *Nutrition*. 2015;31:1-13.
12. Te Morenga L, Mallard S, Mann J. Dietary sugars and body weight: systematic review and meta-analyses of randomised controlled trials and cohort studies. *Bmj*. 2013;346:e7492.
13. Johnson RK, Appel LJ, Brands M, et al. Dietary sugars intake and cardiovascular health: a scientific statement from the American Heart Association. *Circulation*. 2009;120:1011-20.
14. Xi B, Li SS, Liu ZL, et al. Intake of Fruit Juice and Incidence of Type 2 Diabetes: A Systematic Review and Meta-Analysis. *PloS one*. 2014;9:e93471.
15. Moynihan PJ, Kelly SA. Effect on Caries of Restricting Sugars Intake: Systematic Review to Inform WHO Guidelines. *J Dent Res*. 2014;93:8-18.
16. PHE. UK and Ireland prevalence and trends.2013 2 July 2015. Available from: https://www.noo.org.uk/NOO_about_obesity/adult_obesity/UK_prevalence_and_trends.
17. HSCIC. Statistics on Obesity, Physical Activity and Diet: England 20142014 July 2015. Available from: http://www.hscic.gov.uk/catalogue/PUB13648/Obes-phys-acti-diet-eng-2014-rep.pdf.
18. PHE. Adult obesity and type 2 diabetes2014 2 July 2015. Available from: https://www.gov.uk/government/uploads/system/uploads/attachment_data/file/338934/Adult_obesity_and_type_2_diabetes_.pdf.
19. HSCIC. National Diabetes Audit2008 July 2015. Available from: http://www.hscic.gov.uk/catalogue/PUB02580/nati-diab-audi-07-08-exec-summ.pdf.

For peer review only - http://bmjopen.bmj.com/site/about/guidelines.xhtml

20. Houses-of-Parliament. Sugar and Health. POSTNOTE: 4932015 2 December 2015. Available from: http://www.actiononsalt.org.uk/actiononsugar/Sugar%20in%20the%20news/2015/159388.pdf.
21. PHE. National Dental Epidemiology Programme for England: oral health survey of five-year-old children 2012. A report on the prevalence and severity of dental decay2012 2 December 2015. Available from: http://www.nwph.net/dentalhealth/Oral%20Health%205yr%20old%20children%202012%20final%20report%20gateway%20approved.pdf.
22. PHE. Dental public health epidemiology programme. Oral health survey of three-year-old children 2013. A report on the prevalence and severity of dental decay2014 2 December 2015. Available from: http://www.nwph.net/dentalhealth/reports/DPHEP%20for%20England%20OH%20Survey%203yr%202013%20Report.pdf.
23. NHS. Executive Summary: Adult Dental Health Survey2009 2 December 2015. Available from: http://www.hscic.gov.uk/catalogue/PUB01086/adul-dent-heal-surv-summ-them-exec-2009-rep2.pdf.
24. PHE. Sugar reduction Responding to the challenge2014 1 June 2014. Available from: https://www.gov.uk/government/uploads/system/uploads/attachment_data/file/324043/Sugar_Reduction_Responding_to_the_Challenge_26_June.pdf.
25. PHE. Why 5%?2015 Nov 2015. Available from: https://www.gov.uk/government/uploads/system/uploads/attachment_data/file/446010/Why_5___-_The_Science_Behind_SACN.pdf.
26. WHO. Guideline: Sugars intake for adults and children2015 1 July 2015. Available from: http://apps.who.int/iris/bitstream/10665/149782/1/9789241549028_eng.pdf?ua=1.
27. PHE. National Diet and Nutrition Survey: results from Years 1 to 4 (combined) of the rolling programme for 2008 and 2009 to 2011 and 20122014 2 June 2014. Available from: https://www.gov.uk/government/publications/national-diet-and-nutrition-survey-results-from-years-1-to-4-combined-of-the-rolling-programme-for-2008-and-2009-to-2011-and-2012.
28. Rennie KL, Jebb SA, Wright A, et al. Secular trends in under-reporting in young people. *Br J Nutr*. 2005;93:241-7.
29. Hebert JR, Ebbeling CB, Matthews CE, et al. Systematic errors in middle-aged women's estimates of energy intake: comparing three self-report measures to total energy expenditure from doubly labeled water. *Ann Epidemiol*. 2002;12:577-86.
30. Lara JJ, Scott JA, Lean MEJ. Intentional mis-reporting of food consumption and its relationship with body mass index and psychological scores in women. *Journal of Human Nutrition and Dietetics*. 2004;17:209-18.
31. Rennie KL, Coward A, Jebb SA. Estimating under-reporting of energy intake in dietary surveys using an individualised method. *Br J Nutr*. 2007;97:1169-76.
32. Archer E, Hand GA, Blair SN. Correction: Validity of U.S. Nutritional Surveillance: National Health and Nutrition Examination Survey Caloric Energy Intake Data, 1971–2010. *PloS one*. 2013;8:e76632.
33. BSDA. Changing Tastes. The UK Soft Drinks Annual Report 2015. In: Association BSD, editor. 2015.
34. Briggs ADM, Mytton OT, Kehlbacher A, et al. Health impact assessment of the UK soft drinks industry levy: a comparative risk assessment modelling study. *The Lancet Public Health*.2:e15-e22.
35. Seifert SM, Schaechter JL, Hershorin ER, et al. Health Effects of Energy Drinks on Children, Adolescents, and Young Adults. *Pediatrics*. 2011.
36. Hashem KM, He FJ, Jenner KH, et al. Cross-sectional survey of the amount of free sugars and calories in carbonated sugar-sweetened beverages on sale in the UK. *BMJ open*. 2016;6.

For peer review only - http://bmjopen.bmj.com/site/about/guidelines.xhtml

37. Department-of-Health. Guide to creating a front of pack (FoP) nutrition label for pre-packed products sold through retail outlets.2013. Available from: https://www.gov.uk/government/uploads/system/uploads/attachment_data/file/300886/2902158_FoP_Nutrition_2014.pdf.
38. NICE. Preventing obesity and helping people to manage their weight2013 July 2015. Available from: https://www.nice.org.uk/advice/lgb9/chapter/economic-impact.
39. Hex N, Bartlett C, Wright D, et al. Estimating the current and future costs of Type 1 and Type 2 diabetes in the UK, including direct health costs and indirect societal and productivity costs. *Diabetic Medicine*. 2012;29:855-62.
40. Diabetes-UK. Diabetes: Facts and Stats. 2014. Available from: https://www.diabetes.org.uk/Documents/About%20Us/Statistics/Diabetes-key-stats-guidelines-April2014.pdf.
41. Keast RSJ, Swinburn BA, Sayompark D, et al. Caffeine increases sugar-sweetened beverage consumption in a free-living population: a randomised controlled trial. *The British Journal of Nutrition*. 2015;113:366-71.
42. Pinto SC, Bandeca MC, Silva CN, et al. Erosive potential of energy drinks on the dentine surface. *BMC Research Notes*. 2013;6:1-6.
43. Law. EF. Lithuania bans sale of energy drinks to children 2014. Available from: http://www.eurofoodlaw.com/country-reports/eu-member-states/lithuania/lithuania-bans-sale-of-energy-drinks-to-children-104098.htm.
44. WCRF. Use economic tools to address food affordability and purchase incentives 2016 [11 August]. Available from: http://www.wcrf.org/int/policy/nourishing-framework/use-economic-tools.
45. Colchero MA, Popkin BM, Rivera JA, et al. Beverage purchases from stores in Mexico under the excise tax on sugar sweetened beverages: observational study. *Bmj*. 2016;352.
46. Ma Y, He FJ, Yin Y, et al. Gradual reduction of sugar in soft drinks without substitution as a strategy to reduce overweight, obesity, and type 2 diabetes: a modelling study. *The Lancet Diabetes & Endocrinology*. 2016;4:105-14.
47. He FJ, Brinsden HC, Macgregor GA. Salt reduction in the United Kingdom: a successful experiment in public health. *Journal of human hypertension*. 2013.
48. FSA. Low income diet and nutrition survey. Summary of key findings2007 2 December 2015. Available from: http://www.food.gov.uk/multimedia/pdfs/lidnssummary.pdf.
49. Bartolotto C. Does Consuming Sugar and Artificial Sweeteners Change Taste Preferences? *The Permanente journal*. 2015;19:81-4.
50. Hallowell ES, Parikh R, Veldhuizen MG, et al. Flavor Identification and Intensity: Effects of Stimulus Context. *Chem Senses*. 2016;41:249-59.
51. BSDA. Creating New Choices. The 2014 UK Soft Drinks Report2014 23 June 2015. Available from: http://www.britishsoftdrinks.com/write/MediaUploads/Publications/Revised_BSDA_Annual_Report_2014.pdf.
52. Keast RS, Sayompark D, Sacks G, et al. The influence of caffeine on energy content of sugar-sweetened beverages: 'the caffeine-calorie effect'. *European journal of clinical nutrition*. 2011;65:1338-44.
53. Kleiman S, Ng SW, Popkin B. Drinking to our health: can beverage companies cut calories while maintaining profits? *Obesity reviews : an official journal of the International Association for the Study of Obesity*. 2012;13:258-74.
54. Grocer. T. Focus on energy drinks 2015 2015 [25 August 2016]. Available from: http://www.thegrocer.co.uk/reports/category-reports/focus-on-energy-drinks-2015/520323.article.
55. Reissig CJ, Strain EC, Griffiths RR. Caffeinated Energy Drinks -- A Growing Problem. *Drug and alcohol dependence*. 2009;99:1-10.

For peer review only

56. Malinauskas BM, Aeby VG, Overton RF, et al. A survey of energy drink consumption patterns among college students. *Nutrition Journal*. 2007;6:35-.

Legend for figure 1: Free sugars content in supermarket and branded energy drinks (g/serving) in 2017

Legend for figure 2: Calorie content in supermarket and branded energy drinks (g/serving) in 2017

For peer review only

For peer review only - http://bmjopen.bmj.com/site/about/guidelines.xhtml

Table 1: Calorie and sugars content in energy drinks (g/100ml)

	2015			2017		
	N	Calorie (kcal) mean±SD	Free sugars (g) mean±SD	N	Calorie (kcal) mean±SD	Free sugars (g) mean±SD
**Supermarket**	11	48 ± 6 (44-67)	11.0±1.7 (9.8-15.9)	9	39 ± 8 (24-52)	9.1±2.3 (4.9-12.8)
**Branded**	68	48 ± 14 (10-72)	10.7±3.0 (1.9-15.9)	39	45 ± 13 (10-70)	9.8±3.2 (2.1-16.0)
**Total**	79	48± 13 (10 - 72)	10.7±2.8 (1.9 - 15.9)	48	44 ± 13 (10 - 70)	9.7±3.0 (2.1 - 16.0)

Table 2: Calorie and free sugars content in energy drinks (g/serving)

	2015			2017		
	N	Calorie (kcal) mean±SD (range)	Free sugars (g) mean±SD (range)	N	Calorie (kcal) mean±SD	Free sugars (g) mean±SD
**Supermarket**	13	140±48 (110-245)	31.6±10.8 (24.5-54.5)	9	112±58 (60-260)	26.3±14.9 (12.3-64.0)
**Branded**	89	191±81 (50-350)	42.2±18.0 (9.5-78.0)	56	183±73 (50-335)	39.5±18.3 (9.9-80.0)
**Total**	102	184±79 (50 - 350)	40.8±17.6 (9.5 - 78.0)	65	173±75 (50 - 335)	37.7±18.3 (9.9 - 80.0)

For peer review only - http://bmjopen.bmj.com/site/about/guidelines.xhtml

For peer review only

For peer review only

For peer review only - http://bmjopen.bmj.com/site/about/guidelines.xhtml

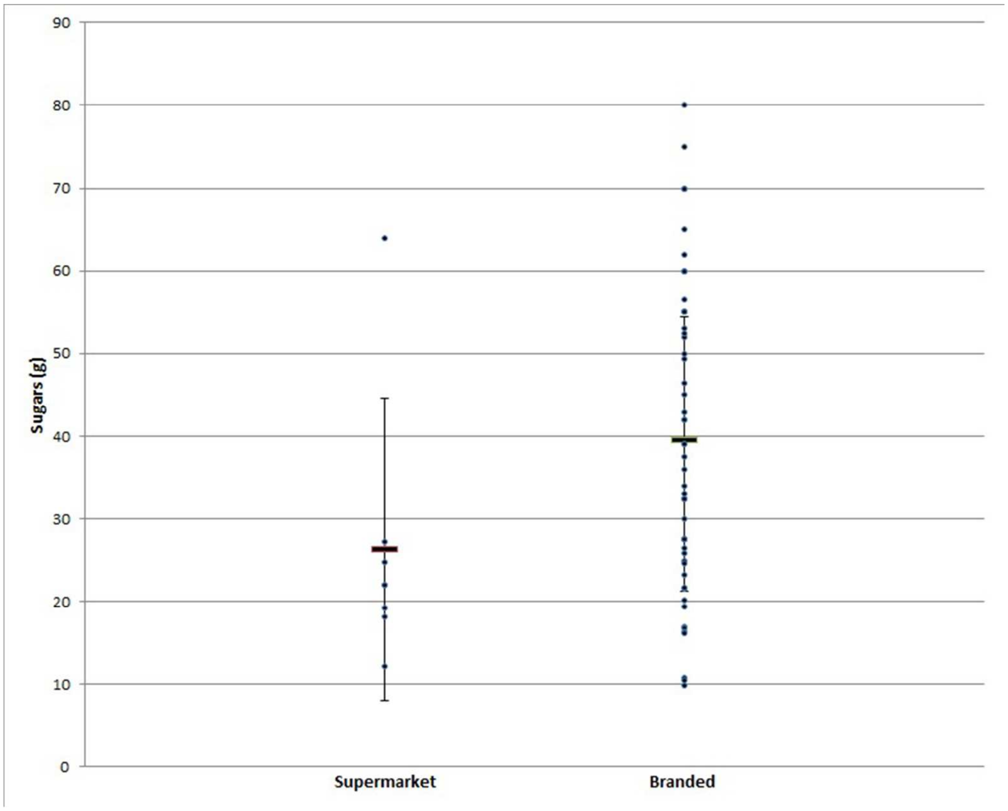

figure 1: Free sugars content in supermarket and branded energy drinks (g/serving) in 2017

66x53mm (300 x 300 DPI)

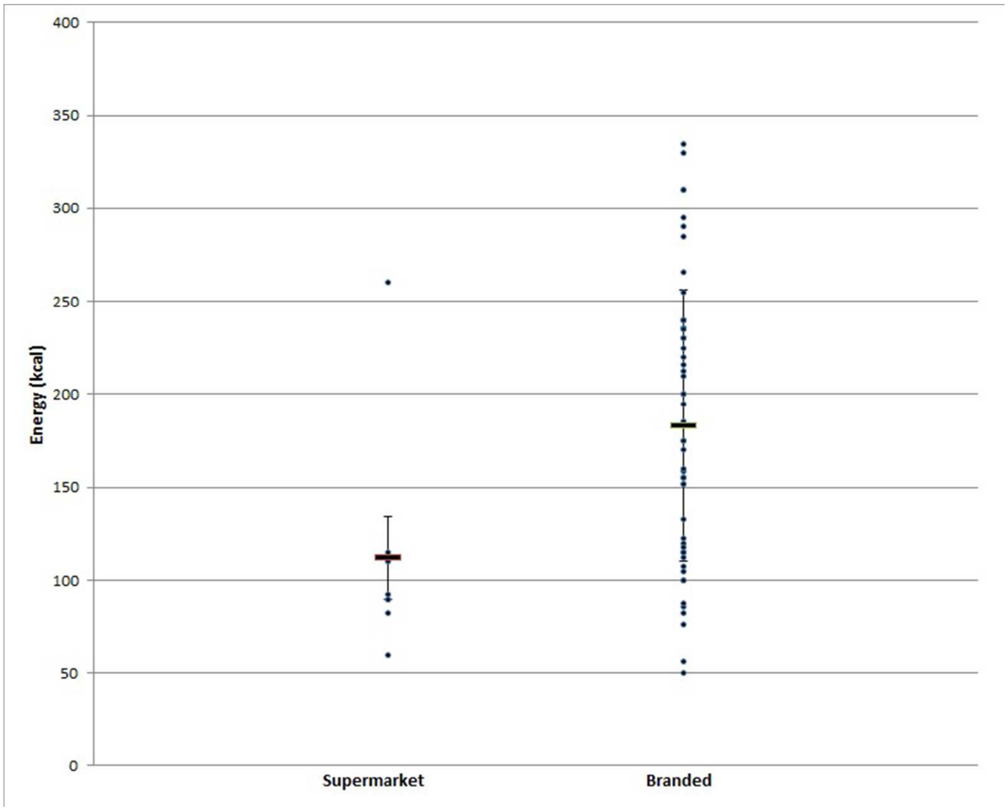

figure 2: Calorie content in supermarket and branded energy drinks (g/serving) in 2017

66x53mm (300 x 300 DPI)

STROBE Statement—checklist of items that should be included in reports of observational studies

	Item No	Recommendation	Page Number Reported
**Title and abstract**	1	(*a*) Indicate the study's design with a commonly used term in the title or the abstract	1-2
		(*b*) Provide in the abstract an informative and balanced summary of what was done and what was found	2
**Introduction**			
Background/rationale	2	Explain the scientific background and rationale for the investigation being reported	3-4
Objectives	3	State specific objectives, including any prespecified hypotheses	2
**Methods**			
Study design	4	Present key elements of study design early in the paper	5-6
Setting	5	Describe the setting, locations, and relevant dates, including periods of recruitment, exposure, follow-up, and data collection	5-6
Participants	6	(*a*) *Cohort study*—Give the eligibility criteria, and the sources and methods of selection of participants. Describe methods of follow-up<br>*Case-control study*—Give the eligibility criteria, and the sources and methods of case ascertainment and control selection. Give the rationale for the choice of cases and controls<br>*Cross-sectional study*—Give the eligibility criteria, and the sources and methods of selection of participants	Not applicable
		(*b*) *Cohort study*—For matched studies, give matching criteria and number of exposed and unexposed<br>*Case-control study*—For matched studies, give matching criteria and the number of controls per case	Not applicable
Variables	7	Clearly define all outcomes, exposures, predictors, potential confounders, and effect modifiers. Give diagnostic criteria, if applicable	5-7
Data sources/ measurement	8*	For each variable of interest, give sources of data and details of methods of assessment (measurement). Describe comparability of assessment methods if there is more than one group	4-7
Bias	9	Describe any efforts to address potential sources of bias	Not applicable
Study size	10	Explain how the study size was arrived at	Not applicable
Quantitative variables	11	Explain how quantitative variables were handled in the analyses. If applicable, describe which groupings were chosen and why	4-7
Statistical methods	12	(*a*) Describe all statistical methods, including those used to control for confounding	7
		(*b*) Describe any methods used to examine subgroups and interactions	7
		(*c*) Explain how missing data were addressed	4-7
		(*d*) *Cohort study*—If applicable, explain how loss to follow-up was addressed<br>*Case-control study*—If applicable, explain how matching of cases and controls was addressed	Not applicable

For peer review only

		Cross-sectional study—If applicable, describe analytical methods taking account of sampling strategy	
		(*e*) Describe any sensitivity analyses	4-7
**Results**			
Participants	13*	(a) Report numbers of individuals at each stage of study—eg numbers potentially eligible, examined for eligibility, confirmed eligible, included in the study, completing follow-up, and analysed	Not applicable
		(b) Give reasons for non-participation at each stage	Not applicable
		(c) Consider use of a flow diagram	Not applicable
Descriptive data	14*	(a) Give characteristics of study participants (eg demographic, clinical, social) and information on exposures and potential confounders	7-8
		(b) Indicate number of participants with missing data for each variable of interest	7-8
		(c) *Cohort study*—Summarise follow-up time (eg, average and total amount)	Not applicable
Outcome data	15*	*Cohort study*—Report numbers of outcome events or summary measures over time	Not applicable
		Case-control study—Report numbers in each exposure category, or summary measures of exposure	7-8
		Cross-sectional study—Report numbers of outcome events or summary measures	Not applicable
Main results	16	(*a*) Give unadjusted estimates and, if applicable, confounder-adjusted estimates and their precision (eg, 95% confidence interval). Make clear which confounders were adjusted for and why they were included	7-11
		(*b*) Report category boundaries when continuous variables were categorized	Fig 1 & 2
		(*c*) If relevant, consider translating estimates of relative risk into absolute risk for a meaningful time period	Not applicable
Other analyses	17	Report other analyses done—eg analyses of subgroups and interactions, and sensitivity analyses	7-8 & Tab 1&2
**Discussion**			
Key results	18	Summarise key results with reference to study objectives	8
Limitations	19	Discuss limitations of the study, taking into account sources of potential bias or imprecision. Discuss both direction and magnitude of any potential bias	14
Interpretation	20	Give a cautious overall interpretation of results considering objectives, limitations, multiplicity of analyses, results from similar studies, and other relevant evidence	8-14
Generalisability	21	Discuss the generalisability (external validity) of the study results	9-14
**Other information**			
Funding	22	Give the source of funding and the role of the funders for the present study and, if applicable, for the original study on which the present article is based	16

*Give information separately for cases and controls in case-control studies and, if applicable, for exposed and unexposed groups in cohort and cross-sectional studies.

For peer review only

Note: An Explanation and Elaboration article discusses each checklist item and gives methodological background and published examples of transparent reporting. The STROBE checklist is best used in conjunction with this article (freely available on the Web sites of PLoS Medicine at http://www.plosmedicine.org/, Annals of Internal Medicine at http://www.annals.org/, and Epidemiology at http://www.epidem.com/). Information on the STROBE Initiative is available at www.strobe-statement.org.

For peer review only

For peer review only - http://bmjopen.bmj.com/site/about/guidelines.xhtml

BMJ Open

Cross-sectional surveys of the amount of sugar, energy and caffeine in sugary energy drinks in the UK between 2015 and 2017

Journal:	*BMJ Open*
Manuscript ID	bmjopen-2017-018136.R1
Article Type:	Research
Date Submitted by the Author:	11-Aug-2017

Complete List of Authors:	Hashem, Kawther; Wolfson Institute of Preventive Medicine, Queen Mary University of London<br>He, Feng; Queen Mary University of London, Wolfson Institute of Preventive Medicine<br>MacGregor, Graham; Barts and the London School of Medicine and Dentistry, Wolfson Institute of Preventive Medicine

**Primary Subject Heading**:	Public health
Secondary Subject Heading:	Health policy, Public health
Keywords:	sugar, calories, energy drinks, caffiene

SCHOLARONE™
Manuscripts

For peer review only

Title: Cross-sectional surveys of the amount of sugar, energy and caffeine in sugary energy drinks in the UK between 2015 and 2017

Kawther M Hashem, Feng J He, Graham A MacGregor

Wolfson Institute of Preventive Medicine, Barts and The London School of Medicine & Dentistry, Queen Mary University of London, UK

Running title: Sugar, energy and caffeine in sugary energy drinks

Correspondence to:

Kawther Hashem

Wolfson Institute of Preventive Medicine, Barts and The London School of Medicine & Dentistry,

Queen Mary University of London, Charterhouse Square, London, EC1M 6BQ.

Tel: 44 (0)20 7882 5941

Fax: 44 (0)20 7882 6270

E-mail: k.hashem@qmul.ac.uk

Keywords: sugar, energy, caffeine, energy drinks

For peer review only - http://bmjopen.bmj.com/site/about/guidelines.xhtml

ABSTRACT

Objectives: To investigate the sugar, energy and caffeine content of sugary energy drinks available in the UK.

Study design: We carried out a cross-sectional survey in 2015 and 2017 of sugary energy drinks available in the main UK retailers.

Methods: The sugar (sugars g/100ml), energy (kcals/100ml), caffeine (mg/100ml) and serving size was collected from product packaging and nutrition information panels of energy drinks available in the nine main UK grocery retailers and three beauty and health food retailers.

Results: The number of formulations (per 100ml) and number of products (per serving) have fallen (from 74 to 49 and from 89 to 59) between 2015 and 2017 respectively. Energy drinks surveyed showed a 10% reduction in sugar, from 10.6 to 9.5 g/100ml (p=0·014) and a 6% reduction in energy content (p=0·007) per 100ml between 2015 and 2017. The average caffeine content of sugary energy drinks, with a warning label, has remained high at 30·8±3.2 in 2015 and 31·6±0·8 mg/100ml in 2017. Despite there being reductions, sugar, energy and caffeine content remain at concerning levels in 2017.

Conclusions: In order to reduce the harmful impact of energy drinks, further reduction in sugar and a reduction in caffeine by reformulation are urgently needed. Other measures such as ban on the sale of energy drinks to children and smaller product sizes should also be explored, while warning labels should be kept. A reduction in sugar, energy and caffeine content and overall energy drinks consumption could be beneficial in reducing sugar, energy and caffeine intake of consumers of energy drinks.

Strengths and limitations of this study

- This paper for the first time investigates and documents the sugar, energy and caffeine content of energy drinks sold in the UK.

For peer review only - http://bmjopen.bmj.com/site/about/guidelines.xhtml

- These results demonstrate that the amount of sugar and energy has fallen, without technical issues, while caffeine levels remain high.
- This paper shows that serving sizes of sugary energy drinks are large and should be reduced.
- The study was based on the levels provided on energy drinks packaging labels in store; hence we relied on the accuracy of the data provided on the label.

INTRODUCTION

In July 2015, the Scientific Advisory Committee on Nutrition (SACN) recommended that average free sugars (sugar) intake, across the UK population, should not exceed 5% of total energy intake. [1] This is in line with the World Health Organisation's new guidelines on sugar intake. [2, 3] SACN also advised that consumption of sugar-sweetened drinks, including energy drinks, should be minimised in children and adults, [1] because high intake of sugar is contributing to obesity, type 2 diabetes and dental caries, [3-11] major public health problems in the UK, [12-19] with significant healthcare costs. [20]

Current average intakes of sugar exceed recommendations in all age groups. [21] The average sugar intake in adults was 59g per day and contributes to 12·1% of energy intake. Children have a higher sugar intake with an average of 61 and 74g per day in 4-10 and 11-18 year olds respectively, which is about three times the new recommendation in 11 to 18-year olds (24g/day). [21]

Soft drinks are the main contributor of sugar intake in children (4-10 years) and teenagers (11-18 years) as well as the second main contributor in adults (18-64 years), contributing to 30%, 40% and 25% of sugar intake respectively. [21] Within soft drinks, energy drinks are a booming sub-category. [22-25]

Products described as 'Energy drinks' are typically non-alcoholic drinks containing a combination of ingredients such as caffeine and/or sugar and/or other ingredients such as guarana, taurine and ginseng, which are known to have stimulant properties and are distinct from sports drinks. [22, 26] Besides the health concerns around sugar content, the high

For peer review only - http://bmjopen.bmj.com/site/about/guidelines.xhtml

levels of caffeine in some energy drinks are associated with chronic sleep loss, addiction/dependence, withdrawal and intoxication. [26-29]

In the UK, sales of energy drinks have increased by 155% between 2006 and 2014, from 235 to 600 million litres, with an average per capita consumption of 9.4 litres in 2014. [30] The leading brands' shares in the UK energy drinks market, by value in 2013/14, were Red Bull (25%), own-label (12%), Monster (10%), Relentless (6%), Rockstar (5%), Mountain Dew (2%), Boost (2%) and Emerge (2%). [31]

Energy drinks consumption in the UK is a growing problem, particularly among children and teenagers. In 2011, a study by the European Food Safety Authority (EFSA) found young people in the UK consumed more energy drinks than in other EU countries (3.1 litres per person per month, compared to 2 litres) [32] and market research data suggest a potential increase in purchase among 10-14 year olds. [31]

The increase in consumption and popularity of energy drinks has raised concerns among the scientific community, governments and the general public about the health effects of these products, particularly among children and adolescents. [26, 33, 34] There have been two policies to tackle sugar and caffeine content of energy drinks. In terms of sugar content, a two-tiered Soft Drinks Industry Levy (SDIL) on the production and importation of sugary drinks will be introduced in 2018. [35] The policy is intended to drive product reformulation and lower sugar consumption; sugary drinks with more than 5g of sugar per 100ml are taxed at a lower rate, with a higher rate for drinks (including energy drinks) exceeding 8g per 100ml. [35] The impact of the levy on sales of energy drinks remains to be seen. [36] In terms of caffeine content, the EU Food Information Regulation requires specific labelling for high caffeine drinks (over 150mg per litre (mg/l). [37] The warning states that the product is not recommended for children. However, these products can be easily purchased and consumed by children.

This cross-sectional survey has been conducted to document levels of sugar and energy in energy drinks, as well as caffeine in the UK. The data available to monitor the energy drinks market, including whether reformulation is taking place, is generally the preserve of

For peer review only - http://bmjopen.bmj.com/site/about/guidelines.xhtml

companies and not in the public domain. The main purpose of this research was to (a) evaluate reformulation of energy drinks to reduce sugar and calorie content between 2015 and 2017, (b) report the variability in sugar and energy content, (c) assess the sugar content in relation to the UK's new daily recommendation for sugar intake and by energy drinks brands and (d) assess the number of products that will be taxed based on the criteria suggested in the SDIL. [35] This study also seeks to quantify the levels of caffeine in sugary energy drinks and determine the products on the market with the back of pack EU caffeine warning.

METHODS

The data was collected from product packaging and nutrient information panels in January 2015 and January 2017. The survey was designed as a comprehensive survey of all energy drinks available in a snapshot in time, using one large outlet for each of the nine main grocery retailers, three beauty and health food retailers and one convenience store. This study used the same study design and procedures as reported in our previous work on carbonated sugar sweetened drinks, which excludes 'energy' drinks). [38]

Definition

'Energy' drinks were defined as any drink products with 'energy', 'energise', 'energiser', 'caffeine' and 'stimulation' in the product name or description e.g. Red Bull Energy Drink, Monster Energy Drink, Relentless Origin Energy Drink and Tropicana Energy Mango and Guava with Passion fruit. Additionally, products that are known to contain high caffeine content but are not described as 'energy drinks' (such as Mountain Dew) were also included.

Since the focus of this study is on product reformulation, products labelled called 'zero' or 'light' or 'no added sugar' etc. were excluded. We also excluded products described solely as 'sports' drinks, which consist primarily of carbohydrates and electrolytes and are intended for athletes to rehydrate after exercise e.g. 'Lucozade Sports'.[22] However, 'Lucozade energy' was included.

For peer review only - http://bmjopen.bmj.com/site/about/guidelines.xhtml

Data Collection

For each ‘energy’ drink the data collected included the company name, product name, pack weight, serving size, sugars (g), energy (kcal) and caffeine (mg) per 100ml and sugars, energy and caffeine per serving. Where data was not available per serving, it was calculated from pack size and per 100ml data. Caffeine content was only collected in a sub-sample of the 2015 products.

Where products only labelled carbohydrate content in 2015 but labelled sugar content in 2017 and the content of all nutrients and ingredients were the same, it was assumed that the sugar content from 2015 was the same as 2017.

All data was double checked after entry, and a further 5% of entries were checked against the original source in a random selection of products.

Stores

Data was collected from each of the major UK supermarkets in store (Aldi, Asda, Lidl, Marks and Spencer, Morrisons, Sainsbury’s, Tesco, The Co-operative and Waitrose), which altogether hold 93.2% of the grocery market share. [39] Three beauty, health and food retailers (Boots, Superdrug and Holland and Barrett) and a Costcutter (convenience store) were also included since they sell a wide variety of branded energy drinks.

Analysis

Per 100ml: Some brands sell the same formulation in different serving sizes. Therefore, the 100ml data only included an example of one formulation regardless of the different serving sizes.

Per serving: The per serving data included all the different serving sizes available apart from 1 litre bottles. Any product with up to 500ml can or bottle size was considered as one serving, regardless of what is stated on the packaging as a serving size e.g. often a 500ml bottle is split into two servings but we consider that most consumers drink these drinks as one serving. 1 litre products were excluded from the per serving analysis since it was

For peer review only

For peer review only - http://bmjopen.bmj.com/site/about/guidelines.xhtml

deemed that the industry standardised serving of 250ml was too little and that consumers are likely to overconsume.

Caffeine: Separate analysis was conducted on the products with the high caffeine warning label (over 150mg per litre (mg/l).

High, Medium and Low criteria for sugars content: The sugar content was compared to the UK front of pack colour-coded labelling for drinks. Portion size criteria applied to portion/serving sizes greater than 150ml. Colour coding is based on the following front of pack colour-coded nutrition labelling criteria (Sugars – Red/High >13·5g/portion or >11·25g/100ml, Amber/Medium >2·5 to ≤11·25/100ml, Green/Low ≤2·25g/100ml). [40]

In addition to the above analyses, the sugar content was compared to the UK's recommendation for sugar intake for adults (30g/day) and 7-10 year old children (24g/day) and to the colour-coded labelling for sugar content. [40] Also, the highest energy containing products were compared to women's daily energy intake (2000 kcal). [41]

Statistical analysis

Comparison among products within each survey

Independent-Samples Mann-Whitney U Test was used to compare the levels of sugar between supermarket own label and branded products.

Comparison of the same products between the two years

For the purpose of this comparison, only the products with data available in both surveys were included in the analysis. Related-Samples Wilcoxon Signed Rank Test was used to examine whether there was a significant change in the sugar, energy and caffeine content of energy drinks from 2015 to 2017.

Data are reported as mean, SD, range as indicated. Significance in all tests carried out was deemed significant as being p<0·05. All data were analysed using SPSS Version 22.

For peer review only - http://bmjopen.bmj.com/site/about/guidelines.xhtml

RESULTS

Sugar, energy and caffeine content per 100 ml in 2015 and 2017

A total of 74 and 49 energy drinks met the inclusion criteria in 2015 and 2017 respectively. The average sugar content was 10.6±2.9 g and 9.7±3.0 g/100ml, with a large variation in sugar content between different energy drinks ranging from 1·9 to 15·9g/100ml and 2·1 to 16·0g/100ml in 2015 and 2017 respectively (Table 1). There were no significant differences in sugar content between supermarket own label and branded products in 2015 (P=0·523) and 2017 P=0·245). The product with the highest and lowest sugar content per 100ml in 2015 and 2017 are highlighted in Table 2.

The average energy content in energy drinks was 47±13 kcal/100ml ranging from 10 to 72 kcal/100ml in 2015 and 44±13 kcal/100ml, ranging from 10 to 70 kcal/100ml in 2017 (Table 1). There were no significant differences in calorie content between supermarket own label and branded products in 2015 (P=0·959) and 2017 (P=0·113).

Among all of the manufacturers with three or more formulations of energy drinks in 2015, Lucozade and Rockstar product ranges contained the highest average sugar and energy per 100ml respectively (Table 3). However, in 2017 the Rockstar product range contained the highest average sugar and energy content per 100ml (Table 3).

Since not all 'energy drinks' have a high caffeine content, we analysed the caffeine, sugar and energy content of the products with the warning label separately . A total of 19 and 41 energy drinks formulations had caffeine content labelled on pack (there may have been more that were labelled but not all were collected in 2015). The average caffeine content in energy drinks was (±SD) 30·8±3.2 in 2015 and 31·6±0·8 mg/100ml in 2017 (Table 1). The levels of sugar, energy and caffeine were similar to the levels in the full sample of energy drinks.

Sugar, energy and caffeine content per serving in 2015 and 2017

A total of 89 and 59 energy drinks products met the inclusion criteria in 2015 and 2017 respectively (Table 4). The total number of products available has fallen between 2015 and

For peer review only - http://bmjopen.bmj.com/site/about/guidelines.xhtml

2017. The total number of products analysed per serving is different to the per 100ml data because some brands sell the same formulation in different serving sizes. The serving data included all the different serving sizes available apart from 1 litre bottles. The serving size varied from 150 to 500ml in 2015 and 250 to 500ml in 2017. 43% and 48% were sold in 500ml cans/bottles in 2015 and 2017 respectively.

The average sugar content in energy drinks was high (±SD) 41·3±17.6 in 2015 and 38.4±18.0g/serving in 2017 (Table 4), more than the entire maximum daily recommendation for sugar intake in the UK for an adult (30g). Indeed, 60% of products in 2015 and 54% in 2017 exceeded the maximum UK's recommendation for sugar intake per serving for an adult (30g/d). Additionally, 87% in 2015 and 78% in 2017 of products exceeded the maximum daily recommendation for sugar intake for a child aged 7-10 years (24g/d). 96% and 95% would receive a 'red' (high) label for sugars per serving (>13·5g/serving) in 2015 and 2017 respectively.

The serving size was significantly larger in branded verses supermarket own label products in 2015 (P=0·007) and 2017 (P=0·003), and as a result the branded products contained on average higher levels of sugar compared to supermarket products per serving in 2015 (P=0·048) and 2017 (P=0·027) (Figure 1 and Table 4).

The average energy content in energy drinks was 186±81kcal and 176±76/serving in 2015 and 2017 respectively. There was a large variation in energy content between different energy drinks ranging from 50 to 350 and 50 to 335kcal/serving in 2015 and 2017 respectively (Figure 2 and Table 4).

The energy drink products with the highest energy content (Lucozade Energy Original 500ml and Rockstar Punched Energy + Punch Fruit Punch Flavour 500ml) in 2015 and 2017 can contribute up to 17-17.5% of a woman's daily energy intake (2000 kcal).[41]

A total of 19 and 41 energy drinks formulations had high caffeine labels on pack (Table 4) in

For peer review only - http://bmjopen.bmj.com/site/about/guidelines.xhtml

2015 and 2017 respectively. The levels of sugar, energy and caffeine were similar to the full sample of energy drinks.

Soft Drink Industry Levy in 2017

A total of 36 products (69%) would be taxed at the higher sugar levy rate (>8g /100ml), 9 products (22%) at the lower rate (8-5g /100ml) and 4 products (9%) wouldn't be taxed (<5g /100ml). [35]

Comparison of the same products between the two years

The overall number of energy drinks formulations has decreased between 2015 and 2017, from 74 to 49 products.

Per 100ml

There were 30 products surveyed repeatedly over the 2 years. The average sugars content per 100ml for these 30 products was 10·6±3·1g in 2015 and 9·5±3·3g in 2017. This represents a reduction of 10% from 2015 to 2017 (p=0·014). The sugar content has reduced in 12 products , remained the same in 16 and has increased in 2. The average calorie content per 100ml was 47±14 kcal in 2015 and 44±15 kcal in 2017, representing a 6% reduction (p=0·007).

There were 12 products surveyed repeatedly in 2015 and 2017 displaying the high caffeine warning label. The caffeine content of these products stayed the same, which is a better reflection of product reformulation.

Note that these averages are slightly different from those when all products were included in each year and this trend analysis reflects reductions made in the same products rather than the overall products available, for a full list of products refer to supplementary file 1.

DISCUSSION

This survey has documented the levels of sugar, energy and caffeine in energy drinks in the UK between 2015 and 2017, a topic which hasn't been previously documented for easy

For peer review only

For peer review only - http://bmjopen.bmj.com/site/about/guidelines.xhtml

access by researchers and policy-makers. It will allow for evaluation of trends in the energy drinks market in the future.

The surveys have shown early changes in the energy drinks market. Formulations (per 100ml) and number of products (per serving) have fallen between 2015 and 2017. This may be due to the pressure on the soft drinks industry to reduce sugary drinks offerings. [42] Furthermore, the energy drinks surveyed showed a small but statistically significant reduction in sugar content; this is likely due to the 12 products which have reduced sugar content between 2015 and 2017, probably as a result of reformulation due to the SDIL.[35] Manufactures of the reduced products have either only reduced sugar or have alternatively reduced sugar and replaced it with non-caloric sweeteners without changing the product name e.g. by calling the product 'light' etc. Given the volume consumed, [22] even small reductions in sugar content of energy drinks would have a significant impact on sugar and energy intake among regular consumers of energy drinks. [43]

This survey may suggest the early success of the SDIL on bringing down sugar levels in energy drinks but we are yet to see if the SDIL will have an impact on overall consumption of energy drinks by reducing sales. [36] Other countries have imposed taxes on soft drinks, including energy drinks, and have seen reductions in sales.[44] [45]

Despite this, sugar content remains at concerning levels, with 95% would receive a 'red' (high) label for sugars per serving in 2017 respectively. There was also a large variation in sugar content between different energy drinks ranging from 2·1 to 16.0g/100ml, which suggests further reductions are possible.

As well as the concerns around the sugar and energy content of energy drinks, there are also concerns about caffeine levels, particularly in the products labelled as high caffeine. In 2017, average caffeine content in energy drinks was 125.3 ± 39.2 mg/serving among the 42 products labelled, almost equivalent to two cups of coffee or four cans of cola.[37] There is also some evidence to suggest that the caffeine content increases sugar-sweetened drinks consumption further and therefore sugar intake too.[46] Since children and teenagers are the main consumers of energy drinks, manufacturers should consider reducing levels of

For peer review only - http://bmjopen.bmj.com/site/about/guidelines.xhtml

For peer review only

caffeine - again through reformulation. Indeed a recent study showed that removing caffeine from sugar-sweetened drinks, along with 10·3% of sugar, has the potential to reduce body weight of adults by 0.6kg, without any change in sugar-sweetened drinks consumption. [47]

It important to note that not all manufacturers label the amount of caffeine in their energy drinks and this is likely because their products do not exceed the cut-off for adding the warning label.[37] Among the companies that do, a warning is stated on the pack, acknowledging the potential danger these products can pose to children. However, these products can be easily purchased and consumed by children. Perhaps the UK should consider restrictions like other countries have. In Lithuania, sales of energy drinks (containing at least 150mg of caffeine per litre) are banned to under-18s. [48] A similar law applies in Sweden and sales of some types of energy drinks are restricted too.[26]

Furthermore, serving sizes of energy drinks matter since the larger the serving size, the more sugar, energy and caffeine is consumed. Indeed, typical serving sizes of energy drinks are larger than other sugar-sweetened drinks; [38] this survey showed that the most common serving size was 500ml. The average sugar content in energy drink in both surveys per serving was more than an adult's entire maximum daily recommendation for sugar intake in the UK. Likewise, 87% in 2015 and 78% in 2017 of the products exceeded the maximum daily recommendation for sugar intake for a child aged 7-10 years (24g/d). [1] The products with the highest sugar and energy content per serving could contribute up 17.5% of a woman's total daily energy intake. Therefore, in order to reduce the amount of sugar, energy and caffeine consumed from energy drinks, there is scope for introducing restrictions on larger serving sizes (500ml bottles and cans). This is a growing problem since the Grocer recently reported an increase in sales of larger cans of energy drinks on the UK market. [23]

Our study was based on sugars, energy and caffeine content data provided on the available energy drinks packaging labels in store on the dates of collection; hence we relied on the accuracy of the data provided on the label. Therefore, it is assumed that the manufacturers

For peer review only - http://bmjopen.bmj.com/site/about/guidelines.xhtml

provide accurate and up to date information in line with EU Regulations. However, future studies should include sugar and energy content determined through laboratory analysis to achieve a better understanding of the true sugar and energy content, and breakdown of the types of sugars. Still, the results of this study are relevant and serve to record the sugar, energy and caffeine content of energy drinks sold the in UK, underpinning future studies and providing evidence for the Treasury and the soft drinks industry, who are reformulating. [49, 50]

CONCLUSION

Factors such as an increase in sales, [24, 25, 30] concerning levels of sugar, energy and caffeine (assessed in this study), ease of access [33] as well as limited regulation have created an environment where energy drinks could pose a major threat to public health. In order to reduce the impact of energy drinks, sugar (and therefore energy) reformulation should continue and begin regarding caffeine content. Other measures such as a ban on the sale of energy drinks to children should be explored, while warning labels should be kept. Can and bottle sizes of energy drinks should also be reduced to reduce overall consumption of sugar, energy and caffeine. A reduction in sugar, energy and caffeine content and overall energy drinks consumption could be beneficial in reducing sugar, energy and caffeine intake of consumers of energy drinks.

COMPETING INTERESTS

All authors have completed the ICMJE uniform disclosure form at www.icmje.org/coi_disclosure.pdf (available on request from the corresponding author) and declare: no support from any organisation for the submitted work; no financial relationships with any organisations that might have an interest in the submitted work in the previous three years. KH is an employee of Consensus Action on Salt & Health (CASH), a nonprofit charitable organisation. FH is a member of CASH and its international branch World Action on Salt & Health (WASH) and does not receive any financial support from CASH or WASH. MacGregor GA is Chairman of Blood Pressure UK (BPUK), Chairman of CASH, Chairman of WASH and Chairman of Action on Sugar. BPUK, CASH , WASH and Action on Sugar are

For peer review only

nonprofit charitable organisations. GM does not receive any financial support from any of these organisations.

CONTRUBUTORSHIP

KH conducted research; KH and FH analysed data; KH wrote the first draft of the manuscript and all authors contributed to the interpretation of the results and revision of the manuscript, and approved the final manuscript. GM had primary responsibility for final content.

FUNDING

This research received no specific grant from any funding agency in the public, commercial or not-for-profit sectors.

DATA SHARING STATEMENT

Extra data is available by emailing Kawther Hashem on k.hashem@qmul.ac.uk.

REFERENCE

1. PHE. Why 5%? 2015 [updated Nov 20151st July 2017]. Available from: https://www.gov.uk/government/uploads/system/uploads/attachment_data/file/446010/Why_5___-_The_Science_Behind_SACN.pdf.
2. WHO. Guideline: Sugars intake for adults and children 2015 [updated 1 July 2015]. Available from: http://apps.who.int/iris/bitstream/10665/149782/1/9789241549028_eng.pdf?ua=1.
3. Moynihan PJ, Kelly SA. Effect on Caries of Restricting Sugars Intake: Systematic Review to Inform WHO Guidelines. *J Dent Res*. 2014;93:8-18.
4. SACN. Carbohydrates and Health 2015 [updated 1 December 2015]. Available from: https://www.gov.uk/government/uploads/system/uploads/attachment_data/file/445503/SACN_Carbohydrates_and_Health.pdf.
5. Romaguera D, Norat T, Wark PA, et al. Consumption of sweet beverages and type 2 diabetes incidence in European adults: results from EPIC-InterAct. *Diabetologia*. 2013;56:1520-30.
6. de Koning L, Malik VS, Rimm EB, et al. Sugar-sweetened and artificially sweetened beverage consumption and risk of type 2 diabetes in men. *The American journal of clinical nutrition*. 2011;93:1321-7.
7. Maki KC, Phillips AK. Dietary substitutions for refined carbohydrate that show promise for reducing risk of type 2 diabetes in men and women. *J Nutr*. 2015;145:159S-63S.
8. Feinman RD, Pogozelski WK, Astrup A, et al. Dietary carbohydrate restriction as the first approach in diabetes management: Critical review and evidence base. *Nutrition*. 2015;31:1-13.
9. Te Morenga L, Mallard S, Mann J. Dietary sugars and body weight: systematic review and meta-analyses of randomised controlled trials and cohort studies. *Bmj*. 2013;346:e7492.

For peer review only - http://bmjopen.bmj.com/site/about/guidelines.xhtml

10. Johnson RK, Appel LJ, Brands M, et al. Dietary sugars intake and cardiovascular health: a scientific statement from the American Heart Association. *Circulation*. 2009;120:1011-20.
11. Xi B, Li SS, Liu ZL, et al. Intake of Fruit Juice and Incidence of Type 2 Diabetes: A Systematic Review and Meta-Analysis. *PloS one*. 2014;9:e93471.
12. PHE. UK and Ireland prevalence and trends.2013 2 July 2015. Available from: https://www.noo.org.uk/NOO_about_obesity/adult_obesity/UK_prevalence_and_trends.
13. HSCIC. Statistics on Obesity, Physical Activity and Diet: England 20142014 July 2015. Available from: http://www.hscic.gov.uk/catalogue/PUB13648/Obes-phys-acti-diet-eng-2014-rep.pdf.
14. PHE. Adult obesity and type 2 diabetes2014 2 July 2015. Available from: https://www.gov.uk/government/uploads/system/uploads/attachment_data/file/338934/Adult_obesity_and_type_2_diabetes_.pdf.
15. HSCIC. National Diabetes Audit2008 July 2015. Available from: http://www.hscic.gov.uk/catalogue/PUB02580/nati-diab-audi-07-08-exec-summ.pdf.
16. Houses-of-Parliament. Sugar and Health. POSTNOTE: 4932015 2 December 2015. Available from: http://www.actiononsalt.org.uk/actiononsugar/Sugar%20in%20the%20news/2015/159388.pdf.
17. PHE. National Dental Epidemiology Programme for England: oral health survey of five-year-old children 2012. A report on the prevalence and severity of dental decay2012 2 December 2015. Available from: http://www.nwph.net/dentalhealth/Oral%20Health%205yr%20old%20children%202012%20final%20report%20gateway%20approved.pdf.
18. PHE. Dental public health epidemiology programme. Oral health survey of three-year-old children 2013. A report on the prevalence and severity of dental decay2014 2 December 2015. Available from: http://www.nwph.net/dentalhealth/reports/DPHEP%20for%20England%20OH%20Survey%203yr%202013%20Report.pdf.
19. NHS. Executive Summary: Adult Dental Health Survey2009 2 December 2015. Available from: http://www.hscic.gov.uk/catalogue/PUB01086/adul-dent-heal-surv-summ-them-exec-2009-rep2.pdf.
20. PHE. Sugar reduction Responding to the challenge 2014 [updated 1 June 2014]. Available from: https://www.gov.uk/government/uploads/system/uploads/attachment_data/file/324043/Sugar_Reduction_Responding_to_the_Challenge_26_June.pdf.
21. PHE. National Diet and Nutrition Survey: results from Years 1 to 4 (combined) of the rolling programme for 2008 and 2009 to 2011 and 2012 2014 [updated 2 June 2014]. Available from: https://www.gov.uk/government/publications/national-diet-and-nutrition-survey-results-from-years-1-to-4-combined-of-the-rolling-programme-for-2008-and-2009-to-2011-and-2012.
22. BSDA. Creating New Choices. The 2014 UK Soft Drinks Report2014 23 June 2015. Available from: http://www.britishsoftdrinks.com/write/MediaUploads/Publications/Revised_BSDA_Annual_Report_2014.pdf.
23. The-Grocer. Red Bull flies high as full-sugar sales surge 2017 [30 July 2017]. Available from: https://www.thegrocer.co.uk/buying-and-supplying/health/red-bull-flies-high-as-full-sugar-sales-surge/550194.article.
24. Kleiman S, Ng SW, Popkin B. Drinking to our health: can beverage companies cut calories while maintaining profits? *Obesity reviews : an official journal of the International Association for the Study of Obesity*. 2012;13:258-74.
25. Grocer. T. Focus on energy drinks 2015 2015 [25 August 2016]. Available from: http://www.thegrocer.co.uk/reports/category-reports/focus-on-energy-drinks-2015/520323.article.

For peer review only - http://bmjopen.bmj.com/site/about/guidelines.xhtml

26. Breda JJ, Whiting SH, Encarnação R, et al. Energy drink consumption in Europe: A review of the risks, adverse health effects and policy options to respond. *Frontiers in Public Health*. 2014;2.
27. Owens J. Insufficient Sleep in Adolescents and Young Adults: An Update on Causes and Consequences. *Pediatrics*. 2014;134:e921-e32.
28. Seifert SM, Schaechter JL, Hershorin ER, et al. Health Effects of Energy Drinks on Children, Adolescents, and Young Adults. *Pediatrics*. 2011.
29. Reissig CJ, Strain EC, Griffiths RR. Caffeinated Energy Drinks -- A Growing Problem. *Drug and alcohol dependence*. 2009;99:1-10.
30. BSDA. Changing Tastes. The UK Soft Drinks Annual Report 2015. In: Association BSD, editor. 2015.
31. Mintel. Sports and Energy Drinks - UK - July 2014 2014.
32. Consortium N-A. Gathering consumption data on specific consumer groups of energy drinks2013 20 March 2017. Available from: http://onlinelibrary.wiley.com/doi/10.2903/sp.efsa.2013.EN-394/pdf.
33. Visram S, Hashem, K,. Energy drinks: what's the evidence? *Food Research Collaboration* 2016.
34. European-Parliament. Schaldemose: "Energy drinks shouldn't have any kind of health claims on them" 2016 [1 September]. Available from: http://www.europarl.europa.eu/news/en/news-room/20160613STO31976/schaldemose-energy-drinks-shouldn't-have-any-kind-of-health-claims-on-them.
35. Gov.uk. Policy paper Soft Drinks Industry Levy 2016 [July 2017]. Available from: https://www.gov.uk/government/publications/soft-drinks-industry-levy/soft-drinks-industry-levy.
36. Briggs ADM, Mytton OT, Kehlbacher A, et al. Health impact assessment of the UK soft drinks industry levy: a comparative risk assessment modelling study. *The Lancet Public Health*.2:e15-e22.
37. FSA. High caffeine 'energy' drinks and other foods containing caffeine n.d [1 July 2017]. Available from: https://www.food.gov.uk/science/additives/energydrinks.
38. Hashem KM, He FJ, Jenner KH, et al. Cross-sectional survey of the amount of free sugars and calories in carbonated sugar-sweetened beverages on sale in the UK. *BMJ open*. 2016;6.
39. Kantar-Worldpanel. Grocery Market Share - Kantar Worldpanel2016 30th April 2016. Available from: http://www.kantarworldpanel.com/en/grocery-market-share/great-britain.
40. Department-of-Health. Guide to creating a front of pack (FoP) nutrition label for pre-packed products sold through retail outlets.2016 1 July 2017. Available from: https://www.food.gov.uk/sites/default/files/multimedia/pdfs/pdf-ni/fop-guidance.pdf.
41. NHS. What should my daily intake of calories be? 2016 [1 July 2017]. Available from: http://www.nhs.uk/chq/pages/1126.aspx?categoryid=51.
42. PHE. Sugar Reduction The evidence for action 2015 2 October 2015 Available from: https://www.gov.uk/government/uploads/system/uploads/attachment_data/file/470179/Sugar_reduction_The_evidence_for_action.pdf.
43. Ma Y, He FJ, Yin Y, et al. Gradual reduction of sugar in soft drinks without substitution as a strategy to reduce overweight, obesity, and type 2 diabetes: a modelling study. *The Lancet Diabetes & Endocrinology*. 2016;4:105-14.
44. WCRF. Use economic tools to address food affordability and purchase incentives 2016 [11 August]. Available from: http://www.wcrf.org/int/policy/nourishing-framework/use-economic-tools.
45. Colchero MA, Popkin BM, Rivera JA, et al. Beverage purchases from stores in Mexico under the excise tax on sugar sweetened beverages: observational study. *Bmj*. 2016;352.
46. Keast RSJ, Swinburn BA, Sayompark D, et al. Caffeine increases sugar-sweetened beverage consumption in a free-living population: a randomised controlled trial. *The British Journal of Nutrition*. 2015;113:366-71.

For peer review only - http://bmjopen.bmj.com/site/about/guidelines.xhtml

47. Keast RS, Sayompark D, Sacks G, et al. The influence of caffeine on energy content of sugar-sweetened beverages: 'the caffeine-calorie effect'. *European journal of clinical nutrition*. 2011;65:1338-44.
48. Law. EF. Lithuania bans sale of energy drinks to children 2014. Available from: http://www.eurofoodlaw.com/country-reports/eu-member-states/lithuania/lithuania-bans-sale-of-energy-drinks-to-children-104098.htm.
49. Lucozade-Ribena-Suntory. Health and Wellbeing n.d. [1st July 2017]. Available from: https://www.lrsuntory.com/health-and-wellbeing/.
50. Tesco. Tesco reduces sugar content in all own brand soft drinks 2016 [1st July 2017]. Available from: https://www.tescoplc.com/news/news-releases/2016/tesco-reduces-sugar-content-in-all-own-brand-soft-drinks.

Legend for figure 1: Sugar content in supermarket and branded energy drinks (g/serving) in 2017

Legend for figure 2: Energy content in supermarket and branded energy drinks (g/serving) in 2017

For peer review only

For peer review only - http://bmjopen.bmj.com/site/about/guidelines.xhtml

Table 1: Sugar, energy and caffeine content in sugary energy drinks per 100ml

	2015				2017			
**Mean±SD (range)**	**N (caffeine)**	**Free sugars (g)**	**Calorie (kcal)**	**Caffeine (mg)**	**N (caffeine)**	**Free sugars (g)**	**Calorie (kcal)**	**Caffeine (mg)**
**All**								
**Own label**	12 (4)	11.0 ± 1.6 (9.8 - 15.9)	48 ± 6 (44 - 67)	30.5 ± 1.0 (30.0 - 32.0)	9 (9)	9.1 ± 2.3 (4.9 - 12.8)	39 ± 8 (24 - 52)	30.9 ± 1.1 (30.0 - 32.0)
**Branded**	62 (17)	10.5 ± 3.1 (1.9 - 15.6)	47 ± 14 (10 - 70)	29.1 ± 8.7 (0.0 - 32.0)	40 (29)	9.8 ± 3.1 (2.1 - 16.0)	45 ± 13 (10 - 70)	31.7 ± 0.6 (30.0 - 32.0)
**Total**	74 (21)	10.6 ± 2.9 (1.9 - 15.9)	47 ± 13 (10 - 70)	29.3 ± 7.7 (0.0 - 32.0)	49 (38)	9.7 ± 3.0 (2.1 - 16.0)	44 ± 13 (10 - 70)	31.5 ± 0.8 (30.0 - 32.0)
**Labelled high caffeine**								
**Supermarket**	3	10.4 ± 0.6 (9.8 - 10.9)	46 ± 2 (44 - 48)	30.7 ± 1.2 (30.0 - 32.0)	9	9.1 ± 2.3 (4.9 - 12.8)	39 ± 8 (24 - 52)	30.9 ± 1.1 (30.0 - 32.0)
**Branded**	16	11.1 ± 3.5 (3.0 - 15.6)	48 ± 13 (17 - 67)	30.9 ± 3.5 (18.0 - 32.0)	32	10.6 ± 2.8 (2.1 - 16.0)	45 ± 11 (10 - 67)	31.8 ± 0.6 (30.0 - 32.0)
**Total**	19	11.0 ± 3.2 (3.0 - 15.6)	48 ± 12 (17 - 67)	30.8 ± 3.2 (18.0 - 32.0)	41	10.2 ± 2.8 (2.1 - 16.0)	44 ± 11 (10 - 67)	31.6 ± 0.8 (30.0 - 32.0)

Table 2: Highest and lowest sugar energy drink per 100ml in 2015 and 2017

	2015		2017	
	**Product name**	**Sugars (g)**	**Product name**	**Sugars (g)**
**Highest**	Sainsbury's Orange Energy Drink	15.9	AG Barr - Rockstar Punched Energy + Guava Tropical Guava Flavour	16.0
**Lowest**	Monster Rehab Green Tea Energy	1.9	Monster Rehab Tea + Still Lemonade + Energy	2.7

For peer review only - http://bmjopen.bmj.com/site/about/guidelines.xhtml

Table 3. Sugar and energy content in sugary energy drinks by manufacturer per 100ml in 2017

2015			2017		
**Manufacturer**	**N**	**Sugar (g) mean±SD (range)**	**Manufacturer**	**N**	**Sugar (g) mean±SD (range)**
Rockstar	8	14.1 ± 1.1 (12.1 - 15.6)	Rockstar	5	14.2 ± 1.5 (12.0 - 16.0)
Lucozade	10	12.5 ± 1.5 (8.7 - 14.0)	Relentless	5	11.1 ± 2.4 (7.5 - 14.0)
Sainsbury's	4	12.1 ± 2.6 (10.6 - 15.9)	Red Bull	3	11.0 ± 0.0 (11.0 - 11.0)
Relentless	5	10.6 ± 0.6 (10.1 - 11.6)	Lidl	5	10.2 ± 1.7 (8.8 - 12.8)
Asda	3	10.5 ± 0.2 (10.4 - 10.7)	Lucozade	6	8.6 ± 3.4 (4.3 - 13.0)
KX	3	10.2 ± 0.2 (10.1 - 10.5)	Monster	8	8.5 ± 2.8 (2.1 - 11.0)
Little Miracles	3	6.4 ± 0.2 (6.2 - 6.6)	Little Miracles	3	5.7 ± 0.5 (5.1 - 6.1)
Monster	7	6.4 ± 4.2 (1.9 - 11.0)			
**Manufacturer**	**N**	**Energy (kcal) mean±SD (range)**	**Manufacturer**	**N**	**Energy (kcal) mean±SD (range)**
Lucozade	10	63.0 ± 4.8 (57.0 - 70.0)	Rockstar	5	60.0 ± 6.6 (51.0 - 67.0)
Rockstar	8	59.9 ± 5.4 (52.0 - 67.0)	Lucozade	6	51.5 ± 14.8 (35.0 - 70.0)
Sainsbury's	4	53.0 ± 9.3 (48.0 - 67.0)	Red Bull	3	45.7 ± 0.6 (45.0 - 46.0)
Asda	3	46.7 ± 1.2 (46.0 - 48.0)	Relentless	5	45.6 ± 9.7 (31.0 - 58.0)
Relentless	5	44.4 ± 2.1 (43.0 - 48.0)	Lidl	5	42.4 ± 6.7 (36.0 - 52.0)
KX	3	44.0 ± 2.0 (42.0 - 46.0)	Monster	8	37.0 ± 12.0 (10.0 - 48.0)
Monster	7	28.6 ± 17.7 (10.0 - 48.0)	Little Miracles	3	24.7 ± 1.5 (23.0 - 26.0)
Little Miracles	3	26.7 ± 0.6 (26.0 - 27.0)			

For peer review only

For peer review only - http://bmjopen.bmj.com/site/about/guidelines.xhtml

Table 4: Sugar, energy and caffeine content in sugary energy drinks per serving

Mean±SD (range)	2015 N (caffeine)	2015 Free sugars (g)	2015 Calorie (kcal)	2015 Caffeine (mg)	2017 N (caffeine)	2017 Free sugars (g)	2017 Calorie (kcal)	2017 Caffeine (mg)
**All**								
**Supermarket**	11 (3)	31.5 ± 11.4 (24.5 - 54.5)	140 ± 51 (110 - 245)	76.7 ± 2.9 (75.0 - 80.0)	8 (8)	27.3 ± 15.6 (12.3 - 64.0)	116 ± 61 (60 - 260)	87.5 ± 29.4 (75.0 - 160.0)
**Branded**	78 (17)	42.7 ± 18.3 (9.5 - 78.0)	193 ± 83 (50 - 350)	120.5 ± 47.6 (0.0 - 160.0)	51 (34)	40.2 ± 18.0 (9.9 - 80.0)	185 ± 75 (50 - 335)	134.2 ± 36.0 (75.0 - 160.0)
**Total**	89 (20)	41.3 ± 17.9 (9.5 - 78.0)	186 ± 81 (50 - 350)	113.9 ± 46.6 (0.0 - 160.0)	59 (42)	38.4 ± 18.1 (9.9 - 80.0)	176 ± 76 (50 - 335)	125.3 ± 39.2 (75.0 - 160.0)
**Labelled high caffeine**								
**Supermarket**	3	26.1 ± 1.4 (24.5 - 27.3)	115 ± 5 (110 - 120)	76.7 ± 2.9 (75.0 - 80.0)	8	27.3 ± 15.6 (12.3 - 64.0)	116 ± 61 (60 - 260)	87.5 ± 29.4 (75.0 - 160.0)
**Branded**	16	48.1 ± 23.1 (9.9 - 78.0)	207 ± 91 (56 - 335)	128.0 ± 37.3 (75.0 - 160.0)	34	44.8 ± 18.0 (9.9 - 80.0)	191 ± 75 (50 - 335)	134.2 ± 36.0 (75.0 - 160.0)
**Total**	19	44.6 ± 22.6 (9.9 - 78.0)	193 ± 90 (56 - 335)	119.9 ± 39.1 (75.0 - 160.0)	42	41.4 ± 18.7 (9.9 - 80.0)	177 ± 78 (50 - 335)	125.3 ± 39.2 (75.0 - 160.0)

For peer review only - http://bmjopen.bmj.com/site/about/guidelines.xhtml

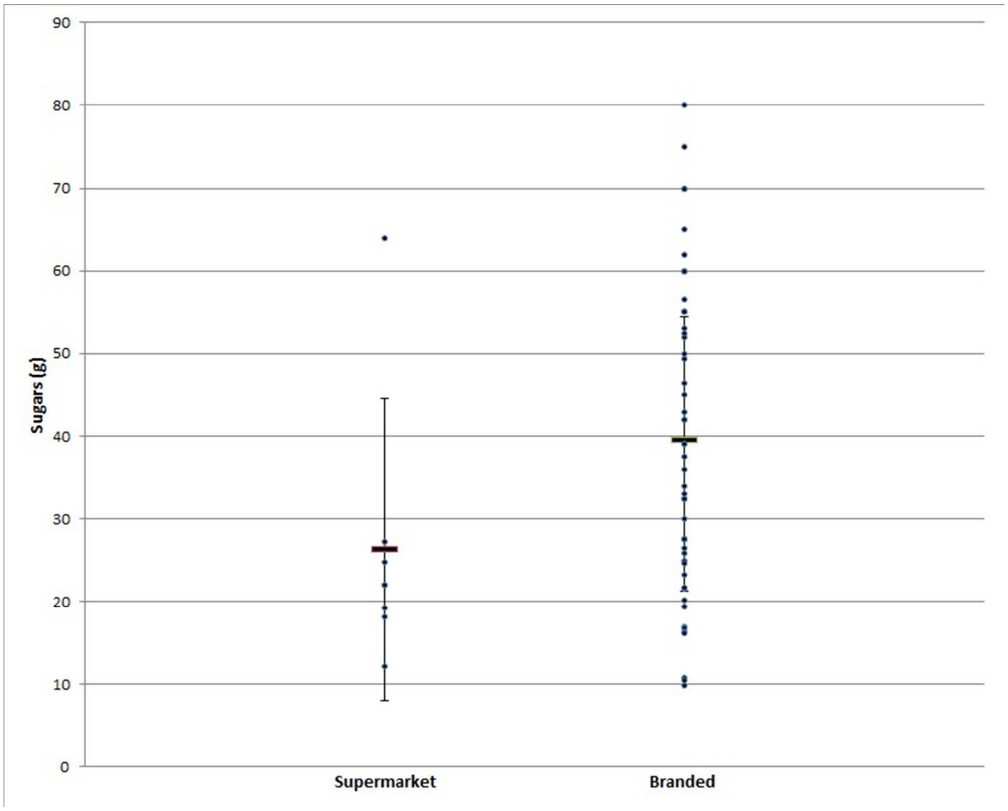

figure 1: Free sugars content in supermarket and branded energy drinks (g/serving) in 2017

66x53mm (300 x 300 DPI)

w only

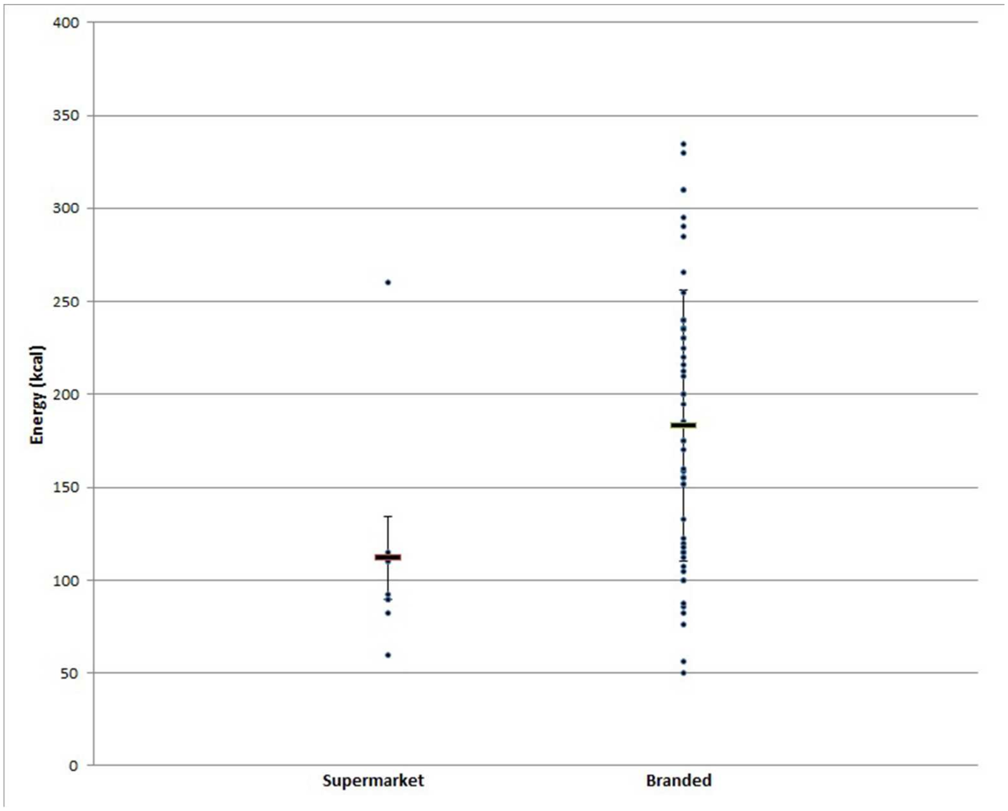

figure 2: Calorie content in supermarket and branded energy drinks (g/serving) in 2017

66x53mm (300 x 300 DPI)

w only

Brand Name	Product Name	Pack Size (ml)	Energy (kcal) per 100ml 2015	Energy (kcal) per 100ml 2017	Sugars (g) per 100ml 2015	Sugars (g) per 100ml 2017	Caffeine (mg) per 100ml 2015	Caffeine (mg) per 100ml 2017
PepsiCo	Tropicana Energy Mango and Guava with Passionfruit 150ml	150	53		10.9			
PepsiCo	Tropicana Energy Pineapple, Mango and Banana 150ml	150	49		9.8			
Tesco	Tesco Blue Spark 6 x 250ml	250	44	24	9.8	4.9	32.0	30.0
Lidl	Lidl Freeway Up Tropical Juiced Energy Drink 250ml	250	46	36	10.5	8.8		32.0
Lidl	Lidl Freeway Up Berry Juiced Energy Drink 250ml	250		36		8.8		32.0
Asda	Asda Chosen by You Original Blue Charge 250ml	250	48	37	10.7	7.7		30.0
Lucozade	Lucozade Energy Pink Lemonade 250ml	250		40		6.8		
Cott	Emerge Energy Drink Original 250ml	250	42	42	9.8	9.3	30.0	30.0
Scheckter's	Scheckter's Organic Energy 250ml	250	47	43	10.8	10		
Lidl	Lidl Freeway Up Lime Energy Drink 250ml	250		44		10.9		32.0
Lidl	Lidl Freeway Up Classic Stimulation Drink 250ml	250		44		9.9		30.0
Red Bull	Red Bull Energy Drink The Orange edition 250ml	250		45		11		32.0
V	V Guarana Energy Drink 250ml	250	45	45	11.2	11		31.0
Morrisons	Morrisons Source Orange and Passion Fruit Energy Drink 250ml	250	46	46	10.6	10.6	30.0	30.0
Red Bull	Red Bull Energy Drink 250ml	250	46	46	11.0	11		32.0
Dico	MTV UP! Energy Drink Classic 250ml	250		46		11		32.0
Red Bull	Red Bull Energy Drink The Tropical Edition 250ml	250		46		11		32.0
Hergesteilt	Seagull Energy Drink 250ml	250		46		11		32.0
Boost	Boost Energy Original 250ml	250	47	47	10.6	10.6		30.0
Sun Mark	Bulldog Power Energy Drink 250ml	250		49		11		31.5
Lucozade	Lucozade Energy Orange 250ml	250	62	62	12.5	13		
Sainsbury's	Sainsbury's Blue Bolt Cherry & Blackcurrant Energy Drink 250ml	250	48		10.9		30.0	
Sainsbury's	Sainsbury's Blue Bolt Energy Drink 250ml	250	49		10.9			
Sainsbury's	Sainsbury's Blue Bolt Mango & Passion Fruit Energy Drink 250ml	250	48		10.6			
Asda	Asda Chosen by You Orange Twist Blue Charge Energy Drink 250ml	250	46		10.4			
Waitrose	Waitrose Love Life Energy Release Drink 250ml	250	47		10.4			
Big Up	Big Up Power Fruit Punch Energy Drink 250ml	250	52		12.2			
Big Up	Big Up Power Wild Ginger Energy Drink 250ml	250	51		12.1			
SoBe	SoBe Pure Rush Energy Drink Appleberry Burst 250ml	250	54		12.0			
SoBe	SoBe Pure Rush Energy Drink Tropical Twist 250ml	250	54		12.0			
Black	Black Mojito Energy Drink 250ml	250	47		11.3			
Tiger	Tiger Energy Drink 250ml	250	46		10.9			
Black	Black Energy Drink 250ml	250	46		10.8			
Singh is King	Singh is King Energy Drink India's Energy Drink 250ml	250	48		10.8		30.0	
KX	KX Energy Stimulation Drink 250ml	250	44		10.1		32.0	
KX	KX Red Berry Stimulation Drink 250ml	250	42		10.1			
Relentless	Relentless Origin Energy Drink 250ml	250	43		10.1			
EQ8	EQ8 Natural Energy Drink Orange & Passion Fruit 250ml	250	40		9.3			
EQ8	EQ8 Natural Energy Drink Cranberry & Apple 250ml	250	29		6.3			
Organiq	Organiq Natural Energy Drink 269ml	269	42		5.6		32.0	
Atlantic Multipower	Multipower Red Kick 330ml	330	17	17	3	3	32.0	32.0
Little Miracles	Little Miracles Organic Energiser Lemongrass, Orange, Ginger, Ginseng, Agave	330		23		5.1		
Little Miracles	Organic Energy Green Tea, Ginseng, Pomegranate, Acai, Acave 330ml	330	26	25	6.2	5.9		
Little Miracles	Organic Energy White Tea, Ginseng, Cherry, Acai, Agave 330ml	330	27	26	6.3	6.1		
Effect	Effect the Mental Energizer 330ml	330	45		10.7		32.0	
Little BigShot	Little BigShot Energy Mixed Berry 330ml - without caffeine!	330	36		9.0		0.0	
Little Miracles	Organic Energy Black Tea, Ginseng, Peach, Acai, Agave 330ml	330	27		6.6			
Red Bull	Red Bull Energy Drink 355ml	355	46	45	11.0	11		32.0
Innocent	Innocent Super Smoothie Energise 360ml	360	62	60	14.0	10		
Powerade	Powerade Energy Sparkling Berry 375ml	375	44		10.5			
Lucozade	Lucozade Energy Tropical Fusion Pineapple & Kiwi Flavour 6 x 380ml	380		35		4.3		
Lucozade	Lucozade Energy Pink Lemonade 380ml	380	57	40	14.0	6.8		
Lucozade	Lucozade Energy Caribbean Crush 380ml	380	57	40	14.0	6.5		
Lucozade	Lucozade Energy Orange 380ml	380	62	62	12.5	13		
Lucozade	Lucozade Energy Original 380ml	380	70	70	8.7	8.7		
Lucozade	Lucozade Energy Apple 380ml	380	68		13.4			
Lucozade	Lucozade Energy Tropical 380ml	380	70		13.0			
Lucozade	Lucozade Energy Limited Edition Melonade 380ml	380	62		12.4			
Lucozade	Lucozade Energy Cherry 380ml	380	61		12.3			
Lucozade	Lucozade Energy Blackcurrant 380ml	380	61		12.2			
Red Bull	Red Bull Energy Drink 473ml	473	46	45	11.0	11	32.0	32.0
No Fear	No Fear Extreme Energy 485ml	485	46		10.1			
Monster	Monster Rehab Tea + Still Lemonade + Energy 500ml	500	10	10	2.1	2.1		32.0
Relentless	Relentless Energy Drink Cherry 500ml	500		31		7.5		32.0
Monster	Monster Khaos Energy + Juice 500ml	500	34	34	7.8	7.8	32.0	32.0
Lucozade	Lucozade Energy Tropical Fusion Pineapple & Kiwi Flavour 500ml	500		35		4.3		
Monster	Monster Ripper Energy + Juice 500ml	500	47	37	10.6	8.4		32.0
Monster	Monster Juiced Energy + Juice 500ml	500		37		8.4		32.0
Monster	Monster Punch Energy 500ml	500		39		9		32.0
Lucozade	Lucozade Energy Pink Lemonade 500ml	500	57	40	14.0	6.8		
Lucozade	Lucozade Energy Caribbean Crush 500ml	500	57	40	14.0	6.5		
Cott	Emerge Energy Drink Original 500ml	500	42	42	9.8	9.3		30.0
Monster	Monster Energy The Doctor 500ml	500		44		10		32.0
Relentless	Relentless Origin Energy Drink 500ml	500	43	45	10.1	11		32.0
Relentless	Relentless Energy Drink Apple Kiwi 500ml	500		46		11		32.0
KX	KX Energy Stimulation Drink 500ml	500	44	46	10.1	10.5		30.0
Monster	Monster Energy 500ml	500	48	47	11.0	11	32.0	32.0
Boost	Boost Energy Original 500ml	500	47	47	10.9	10.6		30.0
Relentless	Relentless Lemon Ice Energy Drink 500ml	500	48	48	11.6	12		32.0
Sun Mark	Bulldog Power Energy Drink 500ml	500		48		11.3		31.5
Monster	Monster Energy Assault 500ml	500		48		11		32.0
Rockstar	Rockstar Energy Drink 500ml	500		51		12		32.0
Lidl	Lidl Freeway Up Colossus Energy Drink 500ml	500		52		12.8		32.0
Rockstar	Rockstar Xdurance Performance Energy Blueberry, Pomegranate and Acai Flavour 500ml	500	57	57	13.8	14	32.0	32.0
Relentless	Relentless Energy Drink Passion Punch 500ml	500		58		14		32.0
Rockstar	Rockstar Super Sours Energy Drink Bubbleburst 500ml	500	59	59	13.8	14	32.0	32.0
Lucozade	Lucozade Energy Orange 500ml	500	62	62	12.5	12.4		
Lucozade	Lucozade Energy The Brazilian Mango Mandarin 500ml	500	62	62	12.4	12		

For peer review only

Brand Name	Product Name	Pack Size (ml)	Energy (kcal) per 100ml 2015	Energy (kcal) per 100ml 2017	Sugars (g) per 100ml 2015	Sugars (g) per 100ml 2017	Caffeine (mg) per 100ml 2015	Caffeine (mg) per 100ml 2017
Rockstar	Rockstar Juiced Energy + Juice Mango Orange Passion Fruit Flavour 500ml	500	66	66	15.0	15	32.0	32.0
Rockstar	Rockstar Punched Energy + Guava Tropical Guava Flavour 500ml	500	67	67	15.6	16	32.0	32.0
Sainsbury's	Sainsbury's Blue Bolt Cherry & Blackcurrant Energy Drink 500ml	500	48		10.9			
Sainsbury's	Sainsbury's Blue Bolt Energy Drink 500ml	500	49		10.9			
Rockstar	Rockstar Punched Energy + Punch Fruit Punch Flavour 500ml	500	64		15.2		32.0	
Rockstar	Rockstar Super Sours Energy Drink Green Apple 500ml	500	59		13.8			
Rockstar	Rockstar Xdurance Performance Energy Orange Flavour 500ml	500	55		13.5			
Lucozade	Lucozade Energy Blackcurrant 500ml	500	67		13.3			
PepsiCo	Mountain Dew 500ml	500	48		13.0		18.0	
Lucozade	Lucozade Energy Limited Edition Melonade 500ml	500	62		12.4			
Lucozade	Lucozade Energy Cherry 500ml	500	61		12.3			
Rockstar	Rockstar Recovery Energy + Hydration Non-Carbonated Lemonade with Added Juice 500ml	500	52		12.1			
Black	Black Energy Drink Pet 500ml	500	46		10.8		32.0	
Relentless	Relentless Tropical Juiced Energy Drink 500ml	500	44		10.7			
Relentless	Relentless Berry Juiced Energy Drink 500ml	500	44		10.6			
KX	KX Special Edition Jason Plato Energy Stimulation Drink 500ml	500	46		10.5			
Powerade	Powerade Energy Sparkling Berry 500ml	500	44		10.5			
Powerade	Powerade Energy Sparkling Orange 500ml	500	45		10.5			
Relentless	Relentless Orange Energy Drink 500ml	500	43		10.2			
Monster	Monster Energy Export 500ml	500	40		9.0			
Lucozade	Lucozade Energy Original 500ml	500	70		8.7			
Monster	Monster Rehab Tea Orangeade Energy 500ml	500	11		2.1			
Monster	Monster Rehab Green Tea Energy 500ml	500	10		1.9			
Morrisons	Morrisons Original Source Energy Drink 1 Litre	1000	44	33	10.3	7.3	30.0	30.0
Lucozade	Lucozade Energy Tropical Fusion Pineapple & Kiwi Flavour 1L	1000		35		4.3		
Asda	Asda Chosen By You Original Blue Charge 1L	1000		37		7.7		30.0
Lucozade	Lucozade Energy Pink Lemonade 1L	1000	57	40	14.0	6.8		
Lucozade	Lucozade Energy Caribbean Crush litre	1000	57	40	13.5	6.5		
Lucozade	Lucozade Energy The Brazilian Mango Mandarin 1L	1000		62		12		
Lucozade	Lucozade Energy Original 1L	1000	70	70	8.7	8.7		
Sainsbury's	Sainsbury's Orange Energy Drink 1L	1000	67		15.9			
Lucozade	Lucozade Energy Limited Edition Melonade 1L	1000	62		12.4			
Asda	Asda Chosen by You Mixed Fruit Blue Charge Energy Drink 1L	1000	46		10.4			

For peer review only

STROBE Statement—checklist of items that should be included in reports of observational studies

	Item No	Recommendation	Page Number Reported
**Title and abstract**	1	(*a*) Indicate the study's design with a commonly used term in the title or the abstract	1-2
		(*b*) Provide in the abstract an informative and balanced summary of what was done and what was found	2
**Introduction**			
Background/rationale	2	Explain the scientific background and rationale for the investigation being reported	3-4
Objectives	3	State specific objectives, including any prespecified hypotheses	2
**Methods**			
Study design	4	Present key elements of study design early in the paper	5-6
Setting	5	Describe the setting, locations, and relevant dates, including periods of recruitment, exposure, follow-up, and data collection	5-6
Participants	6	(*a*) *Cohort study*—Give the eligibility criteria, and the sources and methods of selection of participants. Describe methods of follow-up<br>*Case-control study*—Give the eligibility criteria, and the sources and methods of case ascertainment and control selection. Give the rationale for the choice of cases and controls<br>*Cross-sectional study*—Give the eligibility criteria, and the sources and methods of selection of participants	Not applicable
		(*b*) *Cohort study*—For matched studies, give matching criteria and number of exposed and unexposed<br>*Case-control study*—For matched studies, give matching criteria and the number of controls per case	Not applicable
Variables	7	Clearly define all outcomes, exposures, predictors, potential confounders, and effect modifiers. Give diagnostic criteria, if applicable	5-7
Data sources/ measurement	8*	For each variable of interest, give sources of data and details of methods of assessment (measurement). Describe comparability of assessment methods if there is more than one group	4-7
Bias	9	Describe any efforts to address potential sources of bias	Not applicable
Study size	10	Explain how the study size was arrived at	Not applicable
Quantitative variables	11	Explain how quantitative variables were handled in the analyses. If applicable, describe which groupings were chosen and why	4-7
Statistical methods	12	(*a*) Describe all statistical methods, including those used to control for confounding	7
		(*b*) Describe any methods used to examine subgroups and interactions	7
		(*c*) Explain how missing data were addressed	4-7
		(*d*) *Cohort study*—If applicable, explain how loss to follow-up was addressed<br>*Case-control study*—If applicable, explain how matching of cases and controls was addressed	Not applicable

For peer review only

		Cross-sectional study—If applicable, describe analytical methods taking account of sampling strategy	
		(*e*) Describe any sensitivity analyses	4-7
**Results**			
Participants	13*	(a) Report numbers of individuals at each stage of study—eg numbers potentially eligible, examined for eligibility, confirmed eligible, included in the study, completing follow-up, and analysed	Not applicable
		(b) Give reasons for non-participation at each stage	Not applicable
		(c) Consider use of a flow diagram	Not applicable
Descriptive data	14*	(a) Give characteristics of study participants (eg demographic, clinical, social) and information on exposures and potential confounders	7-8
		(b) Indicate number of participants with missing data for each variable of interest	7-8
		(c) *Cohort study*—Summarise follow-up time (eg, average and total amount)	Not applicable
Outcome data	15*	*Cohort study*—Report numbers of outcome events or summary measures over time	Not applicable
		Case-control study—Report numbers in each exposure category, or summary measures of exposure	7-8
		Cross-sectional study—Report numbers of outcome events or summary measures	Not applicable
Main results	16	(*a*) Give unadjusted estimates and, if applicable, confounder-adjusted estimates and their precision (eg, 95% confidence interval). Make clear which confounders were adjusted for and why they were included	7-11
		(*b*) Report category boundaries when continuous variables were categorized	Fig 1 & 2
		(*c*) If relevant, consider translating estimates of relative risk into absolute risk for a meaningful time period	Not applicable
Other analyses	17	Report other analyses done—eg analyses of subgroups and interactions, and sensitivity analyses	7-8 & Tab 1&2
**Discussion**			
Key results	18	Summarise key results with reference to study objectives	8
Limitations	19	Discuss limitations of the study, taking into account sources of potential bias or imprecision. Discuss both direction and magnitude of any potential bias	14
Interpretation	20	Give a cautious overall interpretation of results considering objectives, limitations, multiplicity of analyses, results from similar studies, and other relevant evidence	8-14
Generalisability	21	Discuss the generalisability (external validity) of the study results	9-14
**Other information**			
Funding	22	Give the source of funding and the role of the funders for the present study and, if applicable, for the original study on which the present article is based	16

*Give information separately for cases and controls in case-control studies and, if applicable, for exposed and unexposed groups in cohort and cross-sectional studies.

For peer review only

Note: An Explanation and Elaboration article discusses each checklist item and gives methodological background and published examples of transparent reporting. The STROBE checklist is best used in conjunction with this article (freely available on the Web sites of PLoS Medicine at http://www.plosmedicine.org/, Annals of Internal Medicine at http://www.annals.org/, and Epidemiology at http://www.epidem.com/). Information on the STROBE Initiative is available at www.strobe-statement.org.

For peer review only

For peer review only - http://bmjopen.bmj.com/site/about/guidelines.xhtml

BMJ Open

Cross-sectional surveys of the amount of sugar, energy and caffeine in sugary soft drinks marketed and consumed as energy drinks in the UK between 2015 and 2017 - Monitoring reformulation progress

Journal:	*BMJ Open*
Manuscript ID	bmjopen-2017-018136.R2
Article Type:	Research
Date Submitted by the Author:	06-Sep-2017
Complete List of Authors:	Hashem, Kawther; Wolfson Institute of Preventive Medicine, Queen Mary University of London<br>He, Feng; Queen Mary University of London, Wolfson Institute of Preventive Medicine<br>MacGregor, Graham; Barts and the London School of Medicine and Dentistry, Wolfson Institute of Preventive Medicine
**Primary Subject Heading**:	Public health
Secondary Subject Heading:	Health policy, Public health
Keywords:	sugar, calories, energy drinks, caffiene

SCHOLARONE™
Manuscripts

For peer review only

Title: Cross-sectional surveys of the amount of sugar, energy and caffeine in sugary soft drinks marketed and consumed as energy drinks in the UK between 2015 and 2017 - Monitoring reformulation progress

Kawther M Hashem, Feng J He, Graham A MacGregor

Wolfson Institute of Preventive Medicine, Barts and The London School of Medicine & Dentistry, Queen Mary University of London, UK

Running title: Sugar, energy and caffeine in sugary energy drinks

Correspondence to:

Kawther Hashem

Wolfson Institute of Preventive Medicine, Barts and The London School of Medicine & Dentistry,

Queen Mary University of London, Charterhouse Square, London, EC1M 6BQ.

Tel: 44 (0)20 7882 5941

Fax: 44 (0)20 7882 6270

E-mail: k.hashem@qmul.ac.uk

Keywords: sugar, energy, caffeine, energy drinks

For peer review only - http://bmjopen.bmj.com/site/about/guidelines.xhtml

ABSTRACT

Objectives: To investigate the sugar, energy and caffeine content of sugary soft drinks marketed and consumed as energy drinks available in the UK.

Study design: We carried out a cross-sectional survey in 2015 and 2017 of energy drinks available in the main UK retailers.

Methods: The sugar (sugars g/100ml), energy (kcals/100ml), caffeine (mg/100ml) and serving size were collected from product packaging and nutrition information panels of energy drinks available in the nine main UK grocery retailers, three beauty and health retailers and one convenience store.

Results: The number of formulations (per 100ml) and number of products (per serving) have fallen (from 75 to 49 and from 90 to 59) between 2015 and 2017 respectively. Energy drinks surveyed showed a 10% reduction in sugar, from 10.6 to 9.5 g/100ml (p=0·014) and a 6% reduction in energy content (p=0·007) per 100ml between 2015 and 2017. The average caffeine content of energy drinks, with a warning label, has remained high at 30·7±3.0 in 2015 and 31·7±0·7 mg/100ml in 2017. Despite there being reductions, sugar, energy and caffeine content remain at concerning levels in 2017.

Conclusions: In order to reduce the harmful impact of energy drinks, further reduction in sugar and a reduction in caffeine by reformulation are urgently needed. Other measures such as ban on the sale of energy drinks to children and smaller product sizes should also be explored, while warning labels should be kept. A reduction in sugar, energy and caffeine content and overall energy drinks consumption could be beneficial in reducing sugar, energy and caffeine intake of consumers of energy drinks.

Strengths and limitations of this study

- This paper for the first time investigates and documents the sugar, energy and caffeine content of energy drinks sold in the UK.

For peer review only - http://bmjopen.bmj.com/site/about/guidelines.xhtml

- These results demonstrate that the amount of sugar and energy has fallen, without technical issues, while caffeine levels remain high.
- This paper shows that serving sizes of energy drinks are large and should be reduced.
- The study was based on the levels provided on energy drinks packaging labels in store; hence we relied on the accuracy of the data provided on the label.

INTRODUCTION

In July 2015, the Scientific Advisory Committee on Nutrition (SACN) recommended that average free sugars (sugar) intake, across the UK population, should not exceed 5% of total energy intake. [1] This is in line with the World Health Organisation's new guidelines on sugar intake. [2, 3] SACN also advised that consumption of sugar-sweetened drinks, including energy drinks, should be minimised in children and adults, [1] because high intake of sugar is contributing to obesity, type 2 diabetes and dental caries, [3-11] major public health problems in the UK, [12-19] with significant healthcare costs. [20]

The average intakes in 2014 of sugar exceeded recommendations in all age groups. [21] The mean sugar intake in adults was 60g per day and contributes to 12% of daily energy intake. Children have a higher sugar intake with an average of 54 (13.4%) and 73g (15.1%) per day in 4-10 and 11-18 year olds respectively. [21]

Soft drinks are the main contributor of sugar intake in children (4-10 years) and teenagers (11-18 years) as well as the second main contributor in adults (18-64 years), contributing to 30%, 40% and 25% of sugar intake respectively. [21] Within soft drinks, energy drinks are a booming sub-category. [22-25]

Products described as 'energy drinks' by the industry are typically glucose based energy drinks; functional or stimulation energy drinks which claim a particular energy boost from caffeine, guarana, taurine, ginseng or other herbs or some combination of these ingredients, which are known to have stimulant properties and are distinct from sports drinks (which are often described as 'sports', 'isotonic' or 'hypotonic' and hypertonic). [22, 26] Besides the health concerns around sugar content, the high levels of caffeine in some energy drinks are

For peer review only

associated with chronic sleep loss, addiction/dependence, withdrawal and intoxication. [26-29]

In the UK, sales of energy drinks have increased by 155% between 2006 and 2014, from 235 to 600 million litres, with an average per capita consumption of 9.4 litres in 2014. [30] The leading brands' shares in the UK energy drinks market, by value in 2013/14, were Red Bull (25%), own-label (12%), Monster (10%), Relentless (6%), Rockstar (5%), Mountain Dew (2%), Boost (2%) and Emerge (2%). [31]

Energy drinks consumption in the UK is a growing problem, particularly among children and teenagers. In 2011, a study by the European Food Safety Authority found young people in the UK consumed more energy drinks than in other EU countries (3.1 litres per person per month, compared to 2 litres) [32] and market research data suggest a potential increase in purchase among 10-14 year olds. [31]

The increase in consumption and popularity of energy drinks has raised concerns among the scientific community, governments and the general public about the health effects of these products, particularly among children and adolescents. [26, 33, 34] There have been two policies to tackle sugar and caffeine content of energy drinks. In terms of sugar content, a two-tiered Soft Drinks Industry Levy (SDIL) on the production and importation of sugar-sweetened drinks, including energy drinks, will be introduced in 2018. [35] The policy is intended to drive product reformulation and lower sugar consumption. Reformulation is commonly described as efforts by the manufacturer to lower the 'unhealthy' components (e.g. saturated fat, trans fats, sugar, salt) of food and drink product at the time of production, without worsening the profile of other ingredients (e.g. increasing calorie content). [36] The reformulated products become a replacement of an existing product (e.g. the same brand of energy drink with less sugar). The SDIL aims to incentivise manufacturers of sugar-sweetened drinks with more than 8g of sugar, per 100ml, to reduce the levels to below 8g and pay a lower tax rate and manufacturers of sugary drinks with more than 5g of sugar per 100ml, to lower the sugar levels to less than 5g and pay no tax. [35]

For peer review only - http://bmjopen.bmj.com/site/about/guidelines.xhtml

In terms of caffeine content, the EU Food Information Regulation requires specific labelling for high caffeine drinks (over 150mg per litre (mg/l). [37] The warning states that the product is not recommended for children. However, these products can be easily purchased and consumed by children.

This cross-sectional survey has been conducted to document levels of sugar and energy in energy drinks, as well as caffeine in the UK. The data available to monitor the energy drinks market, including whether reformulation is taking place, is generally the preserve of companies and not in the public domain. The main purpose of this research was to (a) report the variability in sugar and energy content in 2015 and 2017, (b) assess the sugar content in relation to the UK's new daily recommendation for sugar intake and by energy drinks brands, (c) quantify the levels of caffeine in energy drinks and determine the products on the market with the back of pack EU caffeine warning, (d) assess the number of products that will be taxed based on the criteria suggested in the SDIL [35] and (e) evaluate reformulation of energy drinks to reduce sugar and energy content between 2015 and 2017.

METHODS

The data was collected from product packaging and nutrient information panels in January 2015 and January 2017. The survey was designed as a comprehensive survey of all energy drinks available in a snapshot in time, using one large outlet for each of the nine main grocery retailers, three beauty and health retailers and one convenience store. This study used the same study design and procedures as reported in our previous work on carbonated sugar sweetened drinks, which excluded energy drinks. [38]

Definition

Energy drinks were defined as any drink products with 'energy', 'energise', 'energiser', 'caffeine' and 'stimulation' in the product name or description e.g. Red Bull Energy Drink, Monster Energy Drink, Relentless Origin Energy Drink and Tropicana Energy Mango and Guava with Passion fruit or products with high caffeine warning label including products not described as energy drink (such as Mountain Dew).

For peer review only - http://bmjopen.bmj.com/site/about/guidelines.xhtml

Since the focus of this study is on product reformulation, products labelled ‘zero’ or ‘light’ or ‘no added sugar’ etc. were excluded. We also excluded products described solely as ‘sports’ drinks, which consist primarily of carbohydrates and electrolytes and are intended for athletes to rehydrate after exercise e.g. ‘Lucozade Sports’.[22] However, ‘Lucozade Energy’ was included.

Data Collection

For each energy drink the data collected included the company name, product name, pack weight, serving size, sugars (g), energy (kcal) and caffeine (mg) per 100ml and per serving. Where data was not available per serving, it was calculated from pack size and per 100ml data. Caffeine content was only collected in a sub-sample of the 2015 products.

Where products only labelled carbohydrate content in 2015 but labelled sugar content in 2017 and the content of all nutrients and ingredients were the same, it was assumed that the sugar content from 2015 was the same as 2017 (applied to only one product).

The data were double checked after entry into excel spreadsheet, and a further 5% of entries were checked against the original source in a random selection of products by the lead author.

Stores

Data was collected from each of the major UK supermarkets in store (Aldi, Asda, Lidl, Marks and Spencer, Morrisons, Sainsbury’s, Tesco, The Co-operative and Waitrose), which altogether hold 93.2% of the grocery market share in the UK. [39] Three beauty, health and food retailers (Boots, Superdrug and Holland and Barrett) and one convenience store (Costcutter) were also included since they sell a wide variety of branded energy drinks.

Analysis

Per 100ml: Some brands sell the same formulation in different serving sizes. Therefore, the 100ml data only included an example of one formulation regardless of the different serving

For peer review only - http://bmjopen.bmj.com/site/about/guidelines.xhtml

sizes.

Per serving: The per serving data included all the different serving sizes available apart from 1 litre bottles. 1 litre products were excluded from the per serving analysis since it was deemed that the industry standardised serving of 250ml was too little and that consumers are likely to overconsume. Any product with up to 500ml can or bottle size was considered as one serving, regardless of what is stated on the packaging as a serving size e.g. often a 500ml bottle is split into two servings but we consider that most consumers drink these drinks as one serving.

Caffeine: Separate analysis was conducted on the products with the high caffeine warning label (over 150mg per litre (mg/l).

High, Medium and Low criteria for sugars content: The sugar content was compared to the UK front of pack colour-coded labelling for drinks. Portion size criteria applied to portion/serving sizes greater than 150ml. Colour coding is based on the following front of pack colour-coded nutrition labelling criteria (Sugars – Red/High >13·5g/portion or >11·25g/100ml, Amber/Medium >2·5 to ≤11·25/100ml, Green/Low ≤2·25g/100ml). [40]

In addition to the above analyses, the sugar content was compared to the UK's recommendation for sugar intake for adults (30g/day) and 7-10 year old children (24g/day). [40] The highest energy containing products were compared to women's daily energy intake (2000 kcal). [41] Also, assess the number of products that will be taxed based on the criteria suggested in the SDIL. [35]

Statistical analysis

Comparison among products within each survey

Independent-Samples Mann-Whitney U Test was used to compare the levels of sugar between supermarket own label and branded products.

For peer review only

For peer review only - http://bmjopen.bmj.com/site/about/guidelines.xhtml

Comparison of the same products between the two years

For the purpose of assessing reformulation, only the products with data available in both surveys were included in this analysis. Related-Samples Wilcoxon Signed Rank Test was used to examine whether there was a significant change in the sugar, energy and caffeine content of energy drinks from 2015 to 2017.

Data are reported as mean, SD, range as indicated. Significance in all tests carried out was deemed significant as being $p<0{\cdot}05$. All data were analysed using SPSS Version 22.

RESULTS

Sugar, energy and caffeine content per 100ml in 2015 and 2017

A total of 75 and 49 energy drinks met the per 100ml inclusion criteria in 2015 and 2017 respectively. The average sugar content was 10.6±2.9 g and 9.7±3.0 g/100ml, with a large variation in sugar content between different energy drinks ranging from 1·9 to 15·9g and 2·1 to 16·0g/100ml in 2015 and 2017 respectively (Table 1). There were no significant differences in sugar content between supermarket own label and branded products in 2015 (P=0·397) and 2017 (P=0·245). The product with the highest and lowest sugar content per 100ml in 2015 and 2017 are highlighted in Table 2.

The average energy content in energy drinks was 47±13 kcal/100ml ranging from 10 to 70 kcal/100ml in 2015 and 44±13 kcal/100ml, ranging from 10 to 70 kcal/100ml in 2017 (Table 1). There were no significant differences in energy content between supermarket own label and branded products in 2015 (P=0·872) and 2017 (P=0·113).

Among all of the manufacturers with three or more formulations of energy drinks in 2015, Lucozade and Rockstar product ranges contained the highest average sugar and energy per 100ml respectively (Table 3). However, in 2017 the Rockstar product range contained the highest average sugar and energy content per 100ml (Table 3), which suggests Lucozade have been reformulating their products.

For peer review only - http://bmjopen.bmj.com/site/about/guidelines.xhtml

Since not all 'energy drinks' have a high caffeine content, we analysed the caffeine, sugar and energy content of the products with the warning label separately. A total of 22 and 40 energy drinks formulations had caffeine content labelled on pack in 2015 and 2017 (there may have been more that were labelled but not all were collected in 2015). The average caffeine content in energy drinks was 30.7±3.0 in 2015 and 31·7±0·7 mg/100ml in 2017 (Table 1). The levels of sugar, energy and caffeine were similar to the levels in the full sample of energy drinks.

Sugar, energy and caffeine content per serving in 2015 and 2017

A total of 90 and 59 energy drinks products met the inclusion criteria in 2015 and 2017 respectively (Table 4). The total number of products available has fallen between 2015 and 2017. The total number of products analysed per serving is different to the per 100ml data because some brands sell the same formulation in different serving sizes. The serving data included all the different serving sizes available apart from 1 litre bottles. The serving size varied from 150 to 500ml in 2015 and 250 to 500ml in 2017. 42% and 48% were sold in 500ml cans/bottles in 2015 and 2017 respectively.

The average sugar content in energy drinks was high (±SD) 41·1±17.9 in 2015 and 38.4±18.0g/serving in 2017 (Table 4), more than the entire maximum daily recommendation for sugar intake in the UK for an adult (30g). Indeed, 59% of products in 2015 and 54% in 2017 exceeded the maximum UK's recommendation for sugar intake per serving for an adult (30g/d). Additionally, 86% in 2015 and 78% in 2017 of products exceeded the maximum daily recommendation for sugar intake for a child aged 7-10 years (24g/d). 96% and 95% would receive a 'red' (high) label for sugars per serving (>13·5g/serving) in 2015 and 2017 respectively.

The serving size was significantly larger in branded verses supermarket own label products in 2015 (P=0·004) and 2017 (P=0·002), and as a result the branded products contained on average higher levels of sugar compared to supermarket products per serving in 2015 (P=0·028) and 2017 (P=0·027) (Figure 1 and Table 4).

For peer review only - http://bmjopen.bmj.com/site/about/guidelines.xhtml

The average energy content in energy drinks was 185±81kcal and 176±76/serving in 2015 and 2017 respectively. There was a large variation in energy content between different energy drinks ranging from 50 to 350 and 50 to 335kcal/serving in 2015 and 2017 respectively (Figure 2 and Table 4).

The energy drink products with the highest energy content (Lucozade Energy Original 500ml and Rockstar Punched Energy + Punch Fruit Punch Flavour 500ml) in 2015 and 2017 can contribute up to 17-17.5% of a woman's daily energy intake (2000 kcal).[41]

A total of 22 and 43 energy drinks formulations had high caffeine labels on pack (Table 4) in 2015 and 2017 respectively. The levels of sugar, energy and caffeine were similar to the full sample of energy drinks.

Soft Drink Industry Levy in 2017

A total of 36 products (69%) would be taxed at the higher sugar levy rate (>8g /100ml), 9 products (22%) at the lower rate (8-5g /100ml) and 4 products (9%) wouldn't be taxed (<5g /100ml). [35]

Comparison of the same products between the two years

The overall number of energy drinks formulations has decreased between 2015 and 2017, from 74 to 49 products.

Reformulation

There were 30 products surveyed repeatedly in 2015 and in 2017. The average sugar content per 100ml for these 30 products was 10·6±3·1g in 2015 and 9·5±3·3g in 2017. This represents a reduction of 10% (p=0·014). The sugar content has reduced in 12 products, remained the same in 16 and has increased in 2. The average energy content per 100ml was 47±14 kcal in 2015 and 44±15 kcal in 2017, representing a 6% reduction (p=0·007).

For peer review only - http://bmjopen.bmj.com/site/about/guidelines.xhtml

For peer review only

There were 11 products surveyed repeatedly in 2015 and 2017 displaying the high caffeine warning label. The caffeine content of these products stayed the same, apart from one product, where the caffeine content was reduced, from 32mg to 32mg.

Note that these averages are slightly different from those when all products were included in each year and this trend analysis reflects reductions made in the same products rather than the overall products available, giving a better reflection of reformulation, for a full list of products refer to supplementary file 1.

DISCUSSION

This survey has documented the levels of sugar, energy and caffeine in energy drinks in the UK between 2015 and 2017, a topic which hasn't been previously documented for easy access by researchers and policy-makers. It will allow for evaluation of trends in the energy drinks market in the future.

The surveys have shown early changes in the energy drinks market. Formulations (per 100ml) and number of products (per serving) have fallen between 2015 and 2017. This may be due to the pressure on the soft drinks industry to reduce sugary drinks offerings. [42] Furthermore, the energy drinks surveyed showed a small but statistically significant reduction in sugar content; this is likely due to the 12 products which have reduced sugar content between 2015 and 2017, probably as a result of reformulation due to the SDIL.[35] Manufacturers of the reduced products have either only reduced sugar or have alternatively reduced sugar and replaced it with non-caloric sweeteners without changing the product name e.g. by calling the product 'light' etc. Given the volume consumed, [22] even small reductions in sugar content of energy drinks would have a significant impact on sugar and energy intake among regular consumers of energy drinks. [43]

This survey may suggest the early success of the SDIL on bringing down sugar levels in energy drinks but we are yet to see if the SDIL will have an impact on overall consumption of energy drinks by reducing sales. [44] Other countries have imposed taxes on soft drinks, including energy drinks, and have seen reductions in sales.[45] [46]

For peer review only - http://bmjopen.bmj.com/site/about/guidelines.xhtml

Despite this, sugar content remains at concerning levels, 96% would receive a 'red' (high) label for sugars per serving in 2017 respectively. There was also a large variation in sugar content between different energy drinks ranging from 2·1 to 16.0g/100ml, which suggests further reductions, are possible.

As well as the concerns around the sugar and energy content of energy drinks, there are also concerns about caffeine levels, particularly in the products labelled as high caffeine. In 2017, average caffeine content in energy drinks was 125.3 ± 39.2 mg/serving among the 42 products labelled, almost equivalent to two cups of coffee or four cans of cola.[37] There is also some evidence to suggest that the caffeine content increases sugar-sweetened drinks consumption further and therefore sugar intake too.[47] Since children and teenagers are the main consumers of energy drinks, manufacturers should consider reducing levels of caffeine - again through reformulation. The removal of caffeine also allows for the removal of 10·3% of sugar with affecting taste, which has the potential to reduce body weight of adults by 0.6kg, without any change in sugar-sweetened drinks consumption. [48]

It important to note that not all manufacturers label the amount of caffeine on their energy drinks and this is likely because their products do not exceed the cut-off for adding the warning label.[37] Among the manufacturers that do, a warning is stated on the pack, acknowledging the potential danger these products can pose to children. However, these products can be easily purchased and consumed by children. Perhaps the UK should consider restrictions like other countries have. In Lithuania, sales of energy drinks (containing at least 150mg of caffeine per litre) are banned to under-18s. [49] A similar law applies in Sweden and sales of some types of energy drinks are restricted too.[26]

Furthermore, serving sizes of energy drinks matter since the larger the serving size, the more sugar, energy and caffeine is consumed. Indeed, typical serving sizes of energy drinks are larger than other sugar-sweetened drinks; [38] this survey showed that the most common serving size was 500ml. The average sugar content in energy drink in both surveys per serving was more than an adult's entire maximum daily recommendation for sugar intake in the UK. Likewise, 86% in 2015 and 78% in 2017 of the products exceeded the

For peer review only - http://bmjopen.bmj.com/site/about/guidelines.xhtml

maximum daily recommendation for sugar intake for a child aged 7-10 years (24g/d). [1] The products with the highest sugar and energy content per serving could contribute up 17.5% of a woman's total daily energy intake. Therefore, in order to reduce the amount of sugar, energy and caffeine consumed from energy drinks, there is scope for introducing restrictions on larger serving sizes (500ml bottles and cans). This is a growing problem, that must be tackled, particularly since the Grocer recently reported an increase in sales of larger cans of energy drinks on the UK market. [23]

Our study was based on sugar, energy and caffeine content data provided on the available energy drinks packaging labels in store on the dates of collection; hence we relied on the accuracy of the data provided on the label. Therefore, it is assumed that the manufacturers provide accurate and up to date information in line with EU Regulations. However, future studies should include sugar and energy content determined through laboratory analysis to achieve a better understanding of the true sugar and energy content, and breakdown of the types of sugars.

Also, the data collection process was slightly different in the two surveys. In 2015, caffeine was collected in a sub-sample of products, since the aim was not to collect caffeine at the time, but the data that was collect was included in the analysis, this may not give a full reflection of caffeine content of all products on the market in 2015.

Still, the results of this study are relevant and serve to document the sugar, energy and caffeine content of energy drinks sold the in UK, underpinning future studies and providing evidence for the Treasury and the soft drinks industry, who are reformulating. [50, 51]

CONCLUSION

Factors such as an increase in sales, [24, 25, 30] concerning levels of sugar, energy and caffeine (assessed in this study), ease of access [33], large serving sizes, as well as limited regulation have created an environment where energy drinks could pose a major threat to public health.

In order to reduce the impact of energy drinks, sugar (and therefore energy) reformulation

For peer review only

For peer review only - http://bmjopen.bmj.com/site/about/guidelines.xhtml

should continue and begin regarding caffeine content. Other measures such as a ban on the sale of energy drinks to children should be explored, while warning labels should be kept. Can and bottle sizes of energy drinks should also be decreased to reduce the overall consumption of sugar, energy and caffeine. A reduction in sugar, energy and caffeine content and overall energy drinks consumption could be beneficial in reducing sugar, energy and caffeine intake of consumers of energy drinks.

COMPETING INTERESTS

All authors have completed the ICMJE uniform disclosure form at www.icmje.org/coi_disclosure.pdf (available on request from the corresponding author) and declare: no support from any organisation for the submitted work; no financial relationships with any organisations that might have an interest in the submitted work in the previous three years. KH is an employee of Consensus Action on Salt, Sugar & Health (CASSH), a nonprofit charitable organisation. FH is a member of Consensus Action on Salt and Health (CASH) and its international branch World Action on Salt & Health (WASH) and does not receive any financial support from CASH or WASH. MacGregor GA is Chairman of Blood Pressure UK (BPUK), Chairman of CASSH and Chairman of WASH. BPUK, CASSH and WASH are nonprofit charitable organisations. GM does not receive any financial support from any of these organisations.

CONTRUBUTORSHIP

KH conducted research; KH and FH analysed data; KH wrote the first draft of the manuscript and all authors contributed to the interpretation of the results and revision of the manuscript, and approved the final manuscript. GM had primary responsibility for final content.

FUNDING

This research received no specific grant from any funding agency in the public, commercial or not-for-profit sectors.

DATA SHARING STATEMENT

For peer review only - http://bmjopen.bmj.com/site/about/guidelines.xhtml

Extra data is available by emailing Kawther Hashem on k.hashem@qmul.ac.uk.

REFERENCE

1. PHE. Why 5%? 2015 [1st July 2017]. Available from: https://www.gov.uk/government/uploads/system/uploads/attachment_data/file/446010/Why_5___-_The_Science_Behind_SACN.pdf.
2. WHO. Guideline: Sugars intake for adults and children 2015 [updated 1 July 2015]. Available from: http://apps.who.int/iris/bitstream/10665/149782/1/9789241549028_eng.pdf?ua=1.
3. Moynihan PJ, Kelly SA. Effect on Caries of Restricting Sugars Intake: Systematic Review to Inform WHO Guidelines. *J Dent Res*. 2014;93:8-18.
4. SACN. Carbohydrates and Health 2015 [updated 1 December 2015]. Available from: https://www.gov.uk/government/uploads/system/uploads/attachment_data/file/445503/SACN_Carbohydrates_and_Health.pdf.
5. Romaguera D, Norat T, Wark PA, et al. Consumption of sweet beverages and type 2 diabetes incidence in European adults: results from EPIC-InterAct. *Diabetologia*. 2013;56:1520-30.
6. de Koning L, Malik VS, Rimm EB, et al. Sugar-sweetened and artificially sweetened beverage consumption and risk of type 2 diabetes in men. *The American journal of clinical nutrition*. 2011;93:1321-7.
7. Maki KC, Phillips AK. Dietary substitutions for refined carbohydrate that show promise for reducing risk of type 2 diabetes in men and women. *J Nutr*. 2015;145:159S-63S.
8. Feinman RD, Pogozelski WK, Astrup A, et al. Dietary carbohydrate restriction as the first approach in diabetes management: Critical review and evidence base. *Nutrition*. 2015;31:1-13.
9. Te Morenga L, Mallard S, Mann J. Dietary sugars and body weight: systematic review and meta-analyses of randomised controlled trials and cohort studies. *Bmj*. 2013;346:e7492.
10. Johnson RK, Appel LJ, Brands M, et al. Dietary sugars intake and cardiovascular health: a scientific statement from the American Heart Association. *Circulation*. 2009;120:1011-20.
11. Xi B, Li SS, Liu ZL, et al. Intake of Fruit Juice and Incidence of Type 2 Diabetes: A Systematic Review and Meta-Analysis. *PloS one*. 2014;9:e93471.
12. PHE. UK and Ireland prevalence and trends.2013 2 July 2015. Available from: https://www.noo.org.uk/NOO_about_obesity/adult_obesity/UK_prevalence_and_trends.
13. HSCIC. Statistics on Obesity, Physical Activity and Diet: England 20142014 July 2015. Available from: http://www.hscic.gov.uk/catalogue/PUB13648/Obes-phys-acti-diet-eng-2014-rep.pdf.
14. PHE. Adult obesity and type 2 diabetes2014 2 July 2015. Available from: https://www.gov.uk/government/uploads/system/uploads/attachment_data/file/338934/Adult_obesity_and_type_2_diabetes_.pdf.
15. HSCIC. National Diabetes Audit2008 July 2015. Available from: http://www.hscic.gov.uk/catalogue/PUB02580/nati-diab-audi-07-08-exec-summ.pdf.
16. Houses-of-Parliament. Sugar and Health. POSTNOTE: 4932015 2 December 2015. Available from: http://www.actiononsalt.org.uk/actiononsugar/Sugar%20in%20the%20news/2015/159388.pdf.
17. PHE. National Dental Epidemiology Programme for England: oral health survey of five-year-old children 2012. A report on the prevalence and severity of dental decay2012 2 December 2015. Available from: http://www.nwph.net/dentalhealth/Oral%20Health%205yr%20old%20children%202012%20final%20report%20gateway%20approved.pdf.
18. PHE. Dental public health epidemiology programme. Oral health survey of three-year-old children 2013. A report on the prevalence and severity of dental decay2014 2 December 2015.

For peer review only - http://bmjopen.bmj.com/site/about/guidelines.xhtml

Available from: http://www.nwph.net/dentalhealth/reports/DPHEP%20for%20England%20OH%20Survey%203yr%202013%20Report.pdf.
19. NHS. Executive Summary: Adult Dental Health Survey2009 2 December 2015. Available from: http://www.hscic.gov.uk/catalogue/PUB01086/adul-dent-heal-surv-summ-them-exec-2009-rep2.pdf.
20. PHE. Sugar reduction Responding to the challenge 2014 [updated 1 June 2014]. Available from: https://www.gov.uk/government/uploads/system/uploads/attachment_data/file/324043/Sugar_Reduction_Responding_to_the_Challenge_26_June.pdf.
21. PHE. NDNS: results from Years 5 and 6 (combined) 2016 [updated 2 June 2016]. Available from: https://www.gov.uk/government/statistics/ndns-results-from-years-5-and-6-combined.
22. BSDA. Creating New Choices. The 2014 UK Soft Drinks Report2014 23 June 2015. Available from: http://www.britishsoftdrinks.com/write/MediaUploads/Publications/Revised_BSDA_Annual_Report_2014.pdf.
23. The-Grocer. Red Bull flies high as full-sugar sales surge 2017 [30 July 2017]. Available from: https://www.thegrocer.co.uk/buying-and-supplying/health/red-bull-flies-high-as-full-sugar-sales-surge/550194.article.
24. Kleiman S, Ng SW, Popkin B. Drinking to our health: can beverage companies cut calories while maintaining profits? *Obesity reviews : an official journal of the International Association for the Study of Obesity*. 2012;13:258-74.
25. Grocer. T. Focus on energy drinks 2015 2015 [25 August 2016]. Available from: http://www.thegrocer.co.uk/reports/category-reports/focus-on-energy-drinks-2015/520323.article.
26. Breda JJ, Whiting SH, Encarnação R, et al. Energy drink consumption in Europe: A review of the risks, adverse health effects and policy options to respond. *Frontiers in Public Health*. 2014;2.
27. Owens J. Insufficient Sleep in Adolescents and Young Adults: An Update on Causes and Consequences. *Pediatrics*. 2014;134:e921-e32.
28. Seifert SM, Schaechter JL, Hershorin ER, et al. Health Effects of Energy Drinks on Children, Adolescents, and Young Adults. *Pediatrics*. 2011.
29. Reissig CJ, Strain EC, Griffiths RR. Caffeinated Energy Drinks -- A Growing Problem. *Drug and alcohol dependence*. 2009;99:1-10.
30. BSDA. Changing Tastes. The UK Soft Drinks Annual Report 2015. In: Association BSD, editor. 2015.
31. Mintel. Sports and Energy Drinks - UK - July 2014 2014.
32. Consortium N-A. Gathering consumption data on specific consumer groups of energy drinks2013 20 March 2017. Available from: http://onlinelibrary.wiley.com/doi/10.2903/sp.efsa.2013.EN-394/pdf.
33. Visram S, Hashem, K,. Energy drinks: what's the evidence? *Food Research Collaboration* 2016.
34. European-Parliament. Schaldemose: "Energy drinks shouldn't have any kind of health claims on them" 2016 [1 September]. Available from: http://www.europarl.europa.eu/news/en/news-room/20160613STO31976/schaldemose-energy-drinks-shouldn't-have-any-kind-of-health-claims-on-them.
35. Gov.uk. Policy paper Soft Drinks Industry Levy 2016 [July 2017]. Available from: https://www.gov.uk/government/publications/soft-drinks-industry-levy/soft-drinks-industry-levy.
36. van Raaij J, Hendriksen M, Verhagen H. Potential for improvement of population diet through reformulation of commonly eaten foods. *Public health nutrition*. 2009;12:325-30.
37. FSA. High caffeine 'energy' drinks and other foods containing caffeine n.d [1 July 2017]. Available from: https://www.food.gov.uk/science/additives/energydrinks.

For peer review only

For peer review only - http://bmjopen.bmj.com/site/about/guidelines.xhtml

38. Hashem KM, He FJ, Jenner KH, et al. Cross-sectional survey of the amount of free sugars and calories in carbonated sugar-sweetened beverages on sale in the UK. *BMJ open*. 2016;6.
39. Kantar-Worldpanel. Grocery Market Share - Kantar Worldpanel2016 30th April 2016. Available from: http://www.kantarworldpanel.com/en/grocery-market-share/great-britain.
40. Department-of-Health. Guide to creating a front of pack (FoP) nutrition label for pre-packed products sold through retail outlets.2016 1 July 2017. Available from: https://www.food.gov.uk/sites/default/files/multimedia/pdfs/pdf-ni/fop-guidance.pdf.
41. NHS. What should my daily intake of calories be? 2016 [1 July 2017]. Available from: http://www.nhs.uk/chq/pages/1126.aspx?categoryid=51.
42. PHE. Sugar Reduction The evidence for action 2015 2 October 2015 Available from: https://www.gov.uk/government/uploads/system/uploads/attachment_data/file/470179/Sugar_reduction_The_evidence_for_action.pdf.
43. Ma Y, He FJ, Yin Y, et al. Gradual reduction of sugar in soft drinks without substitution as a strategy to reduce overweight, obesity, and type 2 diabetes: a modelling study. *The Lancet Diabetes & Endocrinology*. 2016;4:105-14.
44. Briggs ADM, Mytton OT, Kehlbacher A, et al. Health impact assessment of the UK soft drinks industry levy: a comparative risk assessment modelling study. *The Lancet Public Health*.2:e15-e22.
45. WCRF. Use economic tools to address food affordability and purchase incentives 2016 [11 August]. Available from: http://www.wcrf.org/int/policy/nourishing-framework/use-economic-tools.
46. Colchero MA, Popkin BM, Rivera JA, et al. Beverage purchases from stores in Mexico under the excise tax on sugar sweetened beverages: observational study. *Bmj*. 2016;352.
47. Keast RSJ, Swinburn BA, Sayompark D, et al. Caffeine increases sugar-sweetened beverage consumption in a free-living population: a randomised controlled trial. *The British Journal of Nutrition*. 2015;113:366-71.
48. Keast RS, Sayompark D, Sacks G, et al. The influence of caffeine on energy content of sugar-sweetened beverages: 'the caffeine-calorie effect'. *European journal of clinical nutrition*. 2011;65:1338-44.
49. Law. EF. Lithuania bans sale of energy drinks to children 2014. Available from: http://www.eurofoodlaw.com/country-reports/eu-member-states/lithuania/lithuania-bans-sale-of-energy-drinks-to-children-104098.htm.
50. Lucozade-Ribena-Suntory. Health and Wellbeing n.d. [1st July 2017]. Available from: https://www.lrsuntory.com/health-and-wellbeing/.
51. Tesco. Tesco reduces sugar content in all own brand soft drinks 2016 [1st July 2017]. Available from: https://www.tescoplc.com/news/news-releases/2016/tesco-reduces-sugar-content-in-all-own-brand-soft-drinks.

For peer review only

For peer review only - http://bmjopen.bmj.com/site/about/guidelines.xhtml

Legend for figure 1: Sugar content in supermarket and branded energy drinks (g/serving) in 2017

Legend for figure 2: Energy content in supermarket and branded energy drinks (g/serving) in 2017

For peer review only

For peer review only - http://bmjopen.bmj.com/site/about/guidelines.xhtml

Table 1: Sugar, energy and caffeine content in energy drinks per 100ml

	2015				2017			
**Mean±SD (range)**	**N (caffeine)**	**sugar (g)**	**Energy (kcal)**	**Caffeine (mg)**	**N (caffeine)**	**Sugar (g)**	**Energy (kcal)**	**Caffeine (mg)**
**All**								
**Own label**	13 (5)	10.8 ± 1.6 (9.6 - 15.9)	48 ± 6 (44 - 67)	30.4 ± 0.9 (30.0 - 32.0)	9 (9)	9.1 ± 2.3 (4.9 - 12.8)	39 ± 8 (24 - 52)	30.9 ± 1.1 (30.0 - 32.0)
**Branded**	62 (19)	10.5 ± 3.1 (1.9 - 15.6)	47 ± 14 (10 - 70)	29.2 ± 7.8 (0.0 - 32.0)	40 (30)	9.8 ± 3.1 (2.1 - 16.0)	45 ± 13 (10 - 70)	31.7 ± 0.6 (30.0 - 32.0)
**Total**	75 (24)	10.6 ± 2.9 (1.9 - 15.9)	47 ± 13 (10 - 70)	29.4 ± 6.9 (0.0 - 32.0)	49 (39)	9.7 ± 3.0 (2.1 - 16.0)	44 ± 13 (10 - 70)	31.5 ± 0.8 (30.0 - 32.0)
**Labelled high caffeine**								
**Supermarket**	4	10.2 ± 0.6 (9.8 - 10.9)	45 ± 2 (44 - 48)	30.5 ± 1.0 (30.0 - 32.0)	7	9.5 ± 2.5 (4.9 - 12.8)	40 ± 9 (24 - 52)	31.1 ± 1.1 (30.0 - 32.0)
**Branded**	18	11.1 ± 3.3 (3.0 - 15.6)	49 ± 12 (17 - 67)	30.7 ± 3.3 (18.0 - 32.0)	33	10.6 ± 2.8 (2.1 - 16.0)	45 ± 11 (10 - 67)	31.8 ± 0.6 (30.0 - 32.0)
**Total**	22	11.0 ± 3.0 (3.0 - 15.6)	48 ± 11 (17 - 67)	30.7 ± 3.0 (18.0 - 32.0)	40	10.4 ± 2.8 (2.1 - 16.0)	44 ± 11 (10 - 67)	31.7 ± 0.7 (30.0 - 32.0)

Table 2: Highest and lowest sugar energy drink per 100ml in 2015 and 2017

	2015		2017	
	**Product name**	**Sugar (g)**	**Product name**	**Sugar (g)**
**Highest**	Sainsbury's Orange Energy Drink	15.9	AG Barr - Rockstar Punched Energy + Guava Tropical Guava Flavour	16.0
**Lowest**	Monster Rehab Green Tea Energy	1.9	Monster Rehab Tea + Still Lemonade + Energy	2.7

For peer review only - http://bmjopen.bmj.com/site/about/guidelines.xhtml

Table 3. Sugar and energy content in energy drinks by manufacturer per 100ml in 2017

2015			2017		
**Manufacturer**	**N**	**Sugar (g) mean±SD (range)**	**Manufacturer**	**N**	**Sugar (g) mean±SD (range)**
Rockstar	8	14.1 ± 1.1 (12.1 - 15.6)	Rockstar	5	14.2 ± 1.5 (12.0 - 16.0)
Lucozade	10	12.5 ± 1.5 (8.7 - 14.0)	Relentless	5	11.1 ± 2.4 (7.5 - 14.0)
Sainsbury's	4	12.1 ± 2.6 (10.6 - 15.9)	Red Bull	3	11.0 ± 0.0 (11.0 - 11.0)
Relentless	5	10.6 ± 0.6 (10.1 - 11.6)	Lidl	5	10.2 ± 1.7 (8.8 - 12.8)
Asda	3	10.5 ± 0.2 (10.4 - 10.7)	Lucozade	6	8.6 ± 3.4 (4.3 - 13.0)
KX	3	10.2 ± 0.2 (10.1 - 10.5)	Monster	8	8.5 ± 2.8 (2.1 - 11.0)
Little Miracles	3	6.4 ± 0.2 (6.2 - 6.6)	Little Miracles	3	5.7 ± 0.5 (5.1 - 6.1)
Monster	7	6.4 ± 4.2 (1.9 - 11.0)			
**Manufacturer**	**N**	**Energy (kcal) mean±SD (range)**	**Manufacturer**	**N**	**Energy (kcal) mean±SD (range)**
Lucozade	10	63.0 ± 4.8 (57.0 - 70.0)	Rockstar	5	60.0 ± 6.6 (51.0 - 67.0)
Rockstar	8	59.9 ± 5.4 (52.0 - 67.0)	Lucozade	6	51.5 ± 14.8 (35.0 - 70.0)
Sainsbury's	4	53.0 ± 9.3 (48.0 - 67.0)	Red Bull	3	45.7 ± 0.6 (45.0 - 46.0)
Asda	4	10.3 ± 0.5 (9.6 - 10.7)	Relentless	5	45.6 ± 9.7 (31.0 - 58.0)
Relentless	5	44.4 ± 2.1 (43.0 - 48.0)	Lidl	5	42.4 ± 6.7 (36.0 - 52.0)
KX	3	44.0 ± 2.0 (42.0 - 46.0)	Monster	8	37.0 ± 12.0 (10.0 - 48.0)
Monster	7	28.6 ± 17.7 (10.0 - 48.0)	Little Miracles	3	24.7 ± 1.5 (23.0 - 26.0)
Little Miracles	3	26.7 ± 0.6 (26.0 - 27.0)			

For peer review only

For peer review only - http://bmjopen.bmj.com/site/about/guidelines.xhtml

Table 4: Sugar, energy and caffeine content in energy drinks per serving

Mean±SD (range)	2015 N (caffeine)	Sugar (g)	Energy (kcal)	Caffeine (mg)	2017 N (caffeine)	Sugar (g)	Energy (kcal)	Caffeine (mg)
**All**								
**Supermarket**	12 (4)	30.8 ± 11.1 (23.9 - 54.5)	137 ± 49 (110 - 245)	76.2 ± 2.5 (75.0 - 80.0)	8 (8)	27.3 ± 15.6 (12.3 - 64.0)	116 ± 61 (60 - 260)	87.5 ± 29.4 (75.0 - 160.0)
**Branded**	78 (19)	42.7 ± 18.3 (9.5 - 78.0)	193 ± 83 (50 - 350)	115.7 ± 47.1 (0.0 - 160.0)	51 (34)	40.2 ± 18.0 (9.9 - 80.0)	185 ± 75 (50 - 335)	134.2 ± 36.0 (75.0 - 160.0)
**Total**	90 (23)	41.3 ± 17.9 (9.5 - 78.0)	185 ± 81 (50 - 350)	108.9 ± 45.3 (0.0 - 160.0)	59 (43)	38.4 ± 18.1 (9.9 - 80.0)	176 ± 76 (50 - 335)	124.3 ± 39.3 (75.0 - 160.0)
**Labelled high caffeine**								
**Supermarket**	4	25.5 ± 1.6 (23.9 - 27.3)	113 ± 6 (107 - 120)	76.3 ± 2.5 (75.0 - 80.0)	8	27.3 ± 15.6 (12.3 - 64.0)	116 ± 61 (60 - 260)	87.5 ± 29.4 (75.0 - 160.0)
**Branded**	18	46.1 ± 22.4 (9.9 - 78.0)	199 ± 89 (56 - 335)	122.1 ± 40.0 (75.0 - 160.0)	35	44.8 ± 18.0 (9.9 - 80.0)	191 ± 75 (50 - 335)	132.7 ± 36.6 (75.0 - 160.0)
**Total**	22	42.3 ± 21.7 (9.9 - 78.0)	183 ± 87 (56 - 335)	113.8 ± 39.5 (75.0 - 160.0)	43	41.4 ± 18.7 (9.9 - 80.0)	177 ± 78 (50 - 335)	124.3 ± 39.3 (75.0 - 160.0)

For peer review only

For peer review only - http://bmjopen.bmj.com/site/about/guidelines.xhtml

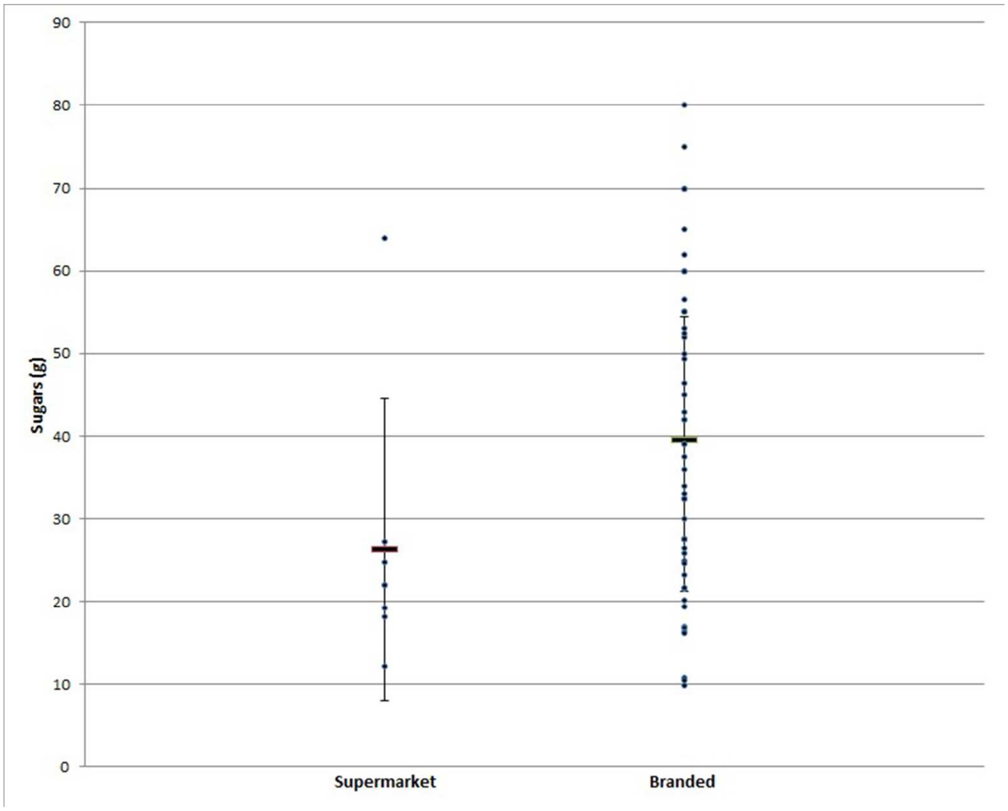

figure 1: Free sugars content in supermarket and branded energy drinks (g/serving) in 2017

66x53mm (300 x 300 DPI)

w only

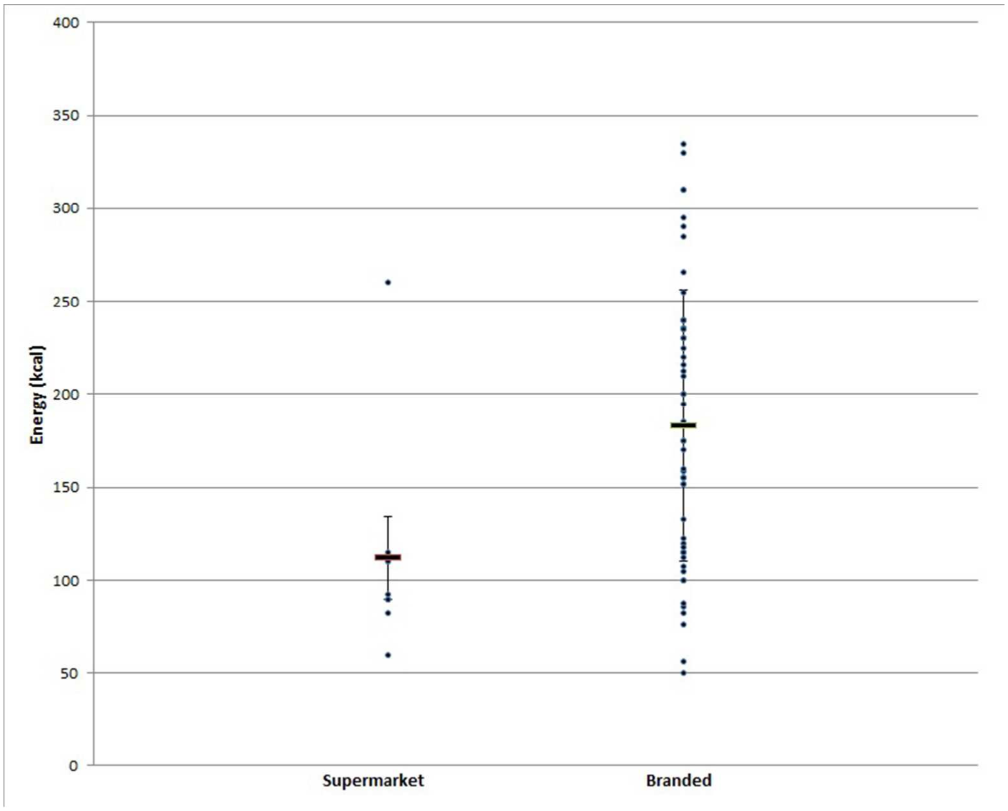

figure 2: Calorie content in supermarket and branded energy drinks (g/serving) in 2017

66x53mm (300 x 300 DPI)

w only

NA - product did not exist in 2015, so caffeine data collection was not applicable
NC - not collected and caffeine label may have not been available
NL - no caffeine warning label

Brand Name	Product Name	Pack Size (ml)	Energy (kcal) per 100ml 2015	Energy (kcal) per 100ml 2017	Sugars (g) per 100ml 2015	Sugars (g) per 100ml 2017	Caffeine (mg) per 100ml 2015	Caffeine (mg) per 100ml 2017
PepsiCo	Tropicana Energy Mango and Guava with Passionfruit 150ml	150	53		10.9		NC	NA
PepsiCo	Tropicana Energy Pineapple, Mango and Banana 150ml	150	49		9.8		NC	NA
Tesco	Tesco Blue Spark 6 x 250ml	250	44	24	9.8	4.9	32.0	30.0
Lidl	Lidl Freeway Up Tropical Juiced Energy Drink 250ml	250	46	36	10.5	8.8	NC	32.0
Lidl	Lidl Freeway Up Berry Juiced Energy Drink 250ml	250		36		8.8	NA	32.0
Asda	Asda Chosen by You Original Blue Charge 250ml	250	48	37	10.7	7.7	NC	30.0
Asda	Asda Chosen by You Chai Spice Flavour Blue Charge 250ml	250	43		9.6		30.0	NA
Lucozade	Lucozade Energy Pink Lemonade 250ml	250		40		6.8	NA	NL
Cott	Emerge Energy Drink Original 250ml	250	42	42	9.8	9.3	30.0	30.0
Scheckter's	Scheckter's Organic Energy 250ml	250	47	43	10.8	10	NC	32.0
Lidl	Lidl Freeway Up Lime Energy Drink 250ml	250		44		10.9	NA	32.0
Lidl	Lidl Freeway Up Classic Stimulation Drink 250ml	250		44		9.9	NA	30.0
Red Bull	Red Bull Energy Drink The Orange edition 250ml	250		45		11	NA	32.0
V	V Guarana Energy Drink 250ml	250	45	45	11.2	11	NC	31.0
Morrisons	Morrisons Source Orange and Passion Fruit Energy Drink 250ml	250	46	46	10.6	10.6	30.0	30.0
Red Bull	Red Bull Energy Drink 250ml	250	46	46	11.0	11	NC	32.0
Dico	MTV UP! Energy Drink Classic 250ml	250		46		11	NA	32.0
Red Bull	Red Bull Energy Drink The Tropical Edition 250ml	250		46		11	NA	32.0
Hergesteilt	Seagull Energy Drink 250ml	250		46		11	NA	32.0
Boost	Boost Energy Original 250ml	250	47	47	10.6	10.6	NC	30.0
Sun Mark	Bulldog Power Energy Drink 250ml	250		49		11	NA	31.5
Lucozade	Lucozade Energy Orange 250ml	250	62	62	12.5	13	NC	NL
Sainsbury's	Sainsbury's Blue Bolt Cherry & Blackcurrant Energy Drink 250ml	250	48		10.9		30.0	NA
Sainsbury's	Sainsbury's Blue Bolt Energy Drink 250ml	250	49		10.9		NC	NA
Sainsbury's	Sainsbury's Blue Bolt Mango & Passion Fruit Energy Drink 250ml	250	48		10.6		NC	NA
Asda	Asda Chosen by You Orange Twist Blue Charge Energy Drink 250ml	250	46		10.4		NC	NA
Waitrose	Waitrose Love Life Energy Release Drink 250ml	250	47		10.4		NC	NA
Big Up	Big Up Power Fruit Punch Energy Drink 250ml	250	52		12.2		30.0	NA
Big Up	Big Up Power Wild Ginger Energy Drink 250ml	250	51		12.1		30.0	NA
SoBe	SoBe Pure Rush Energy Drink Appleberry Burst 250ml	250	54		12.0		NC	NA
SoBe	SoBe Pure Rush Energy Drink Tropical Twist 250ml	250	54		12.0		NC	NA
Black	Black Mojito Energy Drink 250ml	250	47		11.3		NC	NA
Tiger	Tiger Energy Drink 250ml	250	46		10.9		NC	NA
Black	Black Energy Drink 250ml	250	46		10.8		NC	NA
Singh is King	Singh is King Energy Drink India's Energy Drink 250ml	250	48		10.8		30.0	NA
KX	KX Energy Stimulation Drink 250ml	250	44		10.1		32.0	NA
KX	KX Red Berry Stimulation Drink 250ml	250	42		10.1		NC	NA
Relentless	Relentless Origin Energy Drink 250ml	250	43		10.1		NC	NA
EQ8	EQ8 Natural Energy Drink Orange & Passion Fruit 250ml	250	40		9.3		NC	NA
EQ8	EQ8 Natural Energy Drink Cranberry & Apple 250ml	250	29		6.3		NC	NA
Organiq	Organiq Natural Energy Drink 269ml	269	42		5.6		32.0	NA
Atlantic Multipower	Multipower Red Kick 330ml	330	17	17	3	3	32.0	32.0
Little Miracles	Little Miracles Organic Energiser Lemongrass, Orange, Ginger, Ginseng, Agave	330		23		5.1	NA	NL
Little Miracles	Organic Energy Green Tea, Ginseng, Pomegranate, Acai, Acave 330ml	330	26	25	6.2	5.9	NL	NL
Little Miracles	Organic Energy White Tea, Ginseng, Cherry, Acai, Agave 330ml	330	27	26	6.3	6.1	NL	NL
Effect	Effect the Mental Energizer 330ml	330	45		10.7		32.0	NA
Little BigShot	Little BigShot Energy Mixed Berry 330ml - without caffeine!	330	36		9.0		0.0	NA
Little Miracles	Organic Energy Black Tea, Ginseng, Peach, Acai, Agave 330ml	330	27		6.6		NL	NA
Red Bull	Red Bull Energy Drink 355ml	355	46	45	11.0	11	NC	32.0
Innocent	Innocent Super Smoothie Energise 360ml	360	62	60	14.0	10	NL	NL
Powerade	Powerade Energy Sparkling Berry 375ml	375	44		10.5		NC	NA
Lucozade	Lucozade Energy Tropical Fusion Pineapple & Kiwi Flavour 6 x 380ml	380		35		4.3	NA	NL
Lucozade	Lucozade Energy Pink Lemonade 380ml	380	57	40	14.0	6.8	NC	NL
Lucozade	Lucozade Energy Caribbean Crush 380ml	380	57	40	14.0	6.5	NC	NL
Lucozade	Lucozade Energy Orange 380ml	380	62	62	12.5	13	NC	NL
Lucozade	Lucozade Energy Original 380ml	380	70	70	8.7	8.7	NC	NL
Lucozade	Lucozade Energy Apple 380ml	380	68		13.4		NC	NA
Lucozade	Lucozade Energy Tropical 380ml	380	70		13.0		NC	NA
Lucozade	Lucozade Energy Limited Edition Melonade 380ml	380	62		12.4		NC	NA
Lucozade	Lucozade Energy Cherry 380ml	380	61		12.3		NC	NA
Lucozade	Lucozade Energy Blackcurrant 380ml	380	61		12.2		NC	NA
Red Bull	Red Bull Energy Drink 473ml	473	46	45	11.0	11	32.0	32.0
No Fear	No Fear Extreme Energy 485ml	485	46		10.1		NC	NA
Monster	Monster Rehab Tea + Still Lemonade + Energy 500ml	500	10	10	2.1	2.1	NC	32.0
Relentless	Relentless Energy Drink Cherry 500ml	500		31		7.5	NA	32.0
Monster	Monster Khaos Energy + Juice 500ml	500	34	34	7.8	7.8	32.0	32.0
Lucozade	Lucozade Energy Tropical Fusion Pineapple & Kiwi Flavour 500ml	500		35		4.3	NA	NL
Monster	Monster Ripper Energy + Juice 500ml	500	47	37	10.6	8.4	NC	32.0
Monster	Monster Juiced Energy + Juice 500ml	500		37		8.4	NA	32.0
Monster	Monster Punch Energy 500ml	500		39		9	NA	32.0
Lucozade	Lucozade Energy Pink Lemonade 500ml	500	57	40	14.0	6.8	NL	NL
Lucozade	Lucozade Energy Caribbean Crush 500ml	500	57	40	14.0	6.5	NL	NL
Cott	Emerge Energy Drink Original 500ml	500	42	42	9.8	9.3	NC	30.0
Monster	Monster Energy The Doctor 500ml	500		44		10	NA	32.0
Relentless	Relentless Origin Energy Drink 500ml	500	43	45	10.1	11	NC	32.0
Relentless	Relentless Energy Drink Apple Kiwi 500ml	500		46		11	NA	32.0
KX	KX Energy Stimulation Drink 500ml	500	44	46	10.1	10.5	NC	30.0
Monster	Monster Energy 500ml	500	48	47	11.0	11	32.0	32.0
Boost	Boost Energy Original 500ml	500	47	47	10.9	10.6	NC	30.0
Relentless	Relentless Lemon Ice Energy Drink 500ml	500	48	48	11.6	12	NC	32.0
Sun Mark	Bulldog Power Energy Drink 500ml	500		48		11.3	NA	31.5
Monster	Monster Energy Assault 500ml	500		48		11	NA	32.0
Rockstar	Rockstar Energy Drink 500ml	500		51		12	NA	32.0
Lidl	Lidl Freeway Up Colossus Energy Drink 500ml	500		52		12.8	NA	32.0
Rockstar	Rockstar Xdurance Performance Energy Blueberry, Pomegranate and Acai Flavour 500ml	500	57	57	13.8	14	32.0	32.0

For peer review only

Brand Name	Product Name	Pack Size (ml)	Energy (kcal) per 100ml 2015	Energy (kcal) per 100ml 2017	Sugars (g) per 100ml 2015	Sugars (g) per 100ml 2017	Caffeine (mg) per 100ml 2015	Caffeine (mg) per 100ml 2017
Relentless	Relentless Energy Drink Passion Punch 500ml	500		58		14	NA	32.0
Rockstar	Rockstar Super Sours Energy Drink Bubbleburst 500ml	500	59	59	13.8	14	32.0	32.0
Lucozade	Lucozade Energy Orange 500ml	500	62	62	12.5	12.4	NC	NL
Lucozade	Lucozade Energy The Brazilian Mango Mandarin 500ml	500	62	62	12.4	12	NC	NL
Rockstar	Rockstar Juiced Energy + Juice Mango Orange Passion Fruit Flavour 500ml	500	66	66	15.0	15	32.0	32.0
Rockstar	Rockstar Punched Energy + Guava Tropical Guava Flavour 500ml	500	67	67	15.6	16	32.0	32.0
Sainsbury's	Sainsbury's Blue Bolt Cherry & Blackcurrant Energy Drink 500ml	500	48		10.9		NC	NA
Sainsbury's	Sainsbury's Blue Bolt Energy Drink 500ml	500	49		10.9		NC	NA
Rockstar	Rockstar Punched Energy + Punch Fruit Punch Flavour 500ml	500	64		15.2		32.0	NA
Rockstar	Rockstar Super Sours Energy Drink Green Apple 500ml	500	59		13.8		NC	NA
Rockstar	Rockstar Xdurance Performance Energy Orange Flavour 500ml	500	55		13.5		NC	NA
Lucozade	Lucozade Energy Blackcurrant 500ml	500	67		13.3		NC	NA
PepsiCo	Mountain Dew 500ml	500	48		13.0		18.0	NA
Lucozade	Lucozade Energy Limited Edition Melonade 500ml	500	62		12.4		NC	NA
Lucozade	Lucozade Energy Cherry 500ml	500	61		12.3		NC	NA
Rockstar	Rockstar Recovery Energy + Hydration Non-Carbonated Lemonade with Added Juice 500ml	500	52		12.1		NC	NA
Black	Black Energy Drink Pet 500ml	500	46		10.8		32.0	NA
Relentless	Relentless Tropical Juiced Energy Drink 500ml	500	44		10.7		NC	NA
Relentless	Relentless Berry Juiced Energy Drink 500ml	500	44		10.6		NC	NA
KX	KX Special Edition Jason Plato Energy Stimulation Drink 500ml	500	46		10.5		NC	NA
Powerade	Powerade Energy Sparkling Berry 500ml	500	44		10.5		NC	NA
Powerade	Powerade Energy Sparkling Orange 500ml	500	45		10.5		NC	NA
Relentless	Relentless Orange Energy Drink 500ml	500	43		10.2		NC	NA
Monster	Monster Energy Export 500ml	500	40		9.0		NC	NA
Lucozade	Lucozade Energy Original 500ml	500	70		8.7		NC	NA
Monster	Monster Rehab Tea Orangeade Energy 500ml	500	11		2.1		NC	NA
Monster	Monster Rehab Green Tea Energy 500ml	500	10		1.9		NC	NA
Morrisons	Morrisons Original Source Energy Drink 1 Litre	1000	44	33	10.3	7.3	30.0	30.0
Lucozade	Lucozade Energy Tropical Fusion Pineapple & Kiwi Flavour 1L	1000		35		4.3	NA	NL
Asda	Asda Chosen By You Original Blue Charge 1L	1000		37		7.7	NA	30.0
Lucozade	Lucozade Energy Pink Lemonade 1L	1000	57	40	14.0	6.8	NC	NL
Lucozade	Lucozade Energy Caribbean Crush litre	1000	57	40	13.5	6.5	NC	NL
Lucozade	Lucozade Energy The Brazilian Mango Mandarin 1L	1000		62		12	NA	NL
Lucozade	Lucozade Energy Original 1L	1000	70	70	8.7	8.7	NC	NL
Sainsbury's	Sainsbury's Orange Energy Drink 1L	1000	67		15.9		NC	NA
Lucozade	Lucozade Energy Limited Edition Melonade 1L	1000	62		12.4		NC	NA
Asda	Asda Chosen by You Mixed Fruit Blue Charge Energy Drink 1L	1000	46		10.4		NC	NA

For peer review only

STROBE Statement—checklist of items that should be included in reports of observational studies

	Item No	Recommendation	Page Number Reported
**Title and abstract**	1	(*a*) Indicate the study's design with a commonly used term in the title or the abstract	1-2
		(*b*) Provide in the abstract an informative and balanced summary of what was done and what was found	2
**Introduction**			
Background/rationale	2	Explain the scientific background and rationale for the investigation being reported	3-4
Objectives	3	State specific objectives, including any prespecified hypotheses	2
**Methods**			
Study design	4	Present key elements of study design early in the paper	5-6
Setting	5	Describe the setting, locations, and relevant dates, including periods of recruitment, exposure, follow-up, and data collection	5-6
Participants	6	(*a*) *Cohort study*—Give the eligibility criteria, and the sources and methods of selection of participants. Describe methods of follow-up<br>*Case-control study*—Give the eligibility criteria, and the sources and methods of case ascertainment and control selection. Give the rationale for the choice of cases and controls<br>*Cross-sectional study*—Give the eligibility criteria, and the sources and methods of selection of participants	Not applicable
		(*b*) *Cohort study*—For matched studies, give matching criteria and number of exposed and unexposed<br>*Case-control study*—For matched studies, give matching criteria and the number of controls per case	Not applicable
Variables	7	Clearly define all outcomes, exposures, predictors, potential confounders, and effect modifiers. Give diagnostic criteria, if applicable	5-7
Data sources/ measurement	8*	For each variable of interest, give sources of data and details of methods of assessment (measurement). Describe comparability of assessment methods if there is more than one group	4-7
Bias	9	Describe any efforts to address potential sources of bias	Not applicable
Study size	10	Explain how the study size was arrived at	Not applicable
Quantitative variables	11	Explain how quantitative variables were handled in the analyses. If applicable, describe which groupings were chosen and why	4-7
Statistical methods	12	(*a*) Describe all statistical methods, including those used to control for confounding	7
		(*b*) Describe any methods used to examine subgroups and interactions	7
		(*c*) Explain how missing data were addressed	4-7
		(*d*) *Cohort study*—If applicable, explain how loss to follow-up was addressed<br>*Case-control study*—If applicable, explain how matching of cases and controls was addressed	Not applicable

For peer review only

		Cross-sectional study—If applicable, describe analytical methods taking account of sampling strategy	
		(*e*) Describe any sensitivity analyses	4-7
**Results**			
Participants	13*	(a) Report numbers of individuals at each stage of study—eg numbers potentially eligible, examined for eligibility, confirmed eligible, included in the study, completing follow-up, and analysed	Not applicable
		(b) Give reasons for non-participation at each stage	Not applicable
		(c) Consider use of a flow diagram	Not applicable
Descriptive data	14*	(a) Give characteristics of study participants (eg demographic, clinical, social) and information on exposures and potential confounders	7-8
		(b) Indicate number of participants with missing data for each variable of interest	7-8
		(c) *Cohort study*—Summarise follow-up time (eg, average and total amount)	Not applicable
Outcome data	15*	*Cohort study*—Report numbers of outcome events or summary measures over time	Not applicable
		Case-control study—Report numbers in each exposure category, or summary measures of exposure	7-8
		Cross-sectional study—Report numbers of outcome events or summary measures	Not applicable
Main results	16	(*a*) Give unadjusted estimates and, if applicable, confounder-adjusted estimates and their precision (eg, 95% confidence interval). Make clear which confounders were adjusted for and why they were included	7-11
		(*b*) Report category boundaries when continuous variables were categorized	Fig 1 & 2
		(*c*) If relevant, consider translating estimates of relative risk into absolute risk for a meaningful time period	Not applicable
Other analyses	17	Report other analyses done—eg analyses of subgroups and interactions, and sensitivity analyses	7-8 & Tab 1&2
**Discussion**			
Key results	18	Summarise key results with reference to study objectives	8
Limitations	19	Discuss limitations of the study, taking into account sources of potential bias or imprecision. Discuss both direction and magnitude of any potential bias	14
Interpretation	20	Give a cautious overall interpretation of results considering objectives, limitations, multiplicity of analyses, results from similar studies, and other relevant evidence	8-14
Generalisability	21	Discuss the generalisability (external validity) of the study results	9-14
**Other information**			
Funding	22	Give the source of funding and the role of the funders for the present study and, if applicable, for the original study on which the present article is based	16

*Give information separately for cases and controls in case-control studies and, if applicable, for exposed and unexposed groups in cohort and cross-sectional studies.

For peer review only

Note: An Explanation and Elaboration article discusses each checklist item and gives methodological background and published examples of transparent reporting. The STROBE checklist is best used in conjunction with this article (freely available on the Web sites of PLoS Medicine at http://www.plosmedicine.org/, Annals of Internal Medicine at http://www.annals.org/, and Epidemiology at http://www.epidem.com/). Information on the STROBE Initiative is available at www.strobe-statement.org.

For peer review only

BMJ Open

Cross-sectional surveys of the amount of sugar, energy and caffeine in sugary soft drinks marketed and consumed as energy drinks in the UK between 2015 and 2017 - Monitoring reformulation progress

Journal:	*BMJ Open*
Manuscript ID	bmjopen-2017-018136.R3
Article Type:	Research
Date Submitted by the Author:	03-Oct-2017
Complete List of Authors:	Hashem, Kawther; Wolfson Institute of Preventive Medicine, Queen Mary University of London<br>He, Feng; Queen Mary University of London, Wolfson Institute of Preventive Medicine<br>MacGregor, Graham; Barts and the London School of Medicine and Dentistry, Wolfson Institute of Preventive Medicine
**Primary Subject Heading**:	Public health
Secondary Subject Heading:	Health policy, Public health
Keywords:	sugar, calories, energy drinks, caffiene

SCHOLARONE™
Manuscripts

For peer review only

Title: Cross-sectional surveys of the amount of sugar, energy and caffeine in sugary soft drinks marketed and consumed as energy drinks in the UK between 2015 and 2017 - Monitoring reformulation progress

Kawther M Hashem, Feng J He, Graham A MacGregor

Wolfson Institute of Preventive Medicine, Barts and The London School of Medicine & Dentistry, Queen Mary University of London, UK

Running title: Sugar, energy and caffeine in sugary energy drinks

Correspondence to:

Kawther Hashem

Wolfson Institute of Preventive Medicine, Barts and The London School of Medicine & Dentistry,

Queen Mary University of London, Charterhouse Square, London, EC1M 6BQ.

Tel: 44 (0)20 7882 5941

Fax: 44 (0)20 7882 6270

E-mail: k.hashem@qmul.ac.uk

Keywords: sugar, energy, caffeine, energy drinks

For peer review only - http://bmjopen.bmj.com/site/about/guidelines.xhtml

ABSTRACT

Objectives: To investigate the sugar, energy and caffeine content of sugary soft drinks marketed and consumed as energy drinks available in the UK.

Study design: We carried out a cross-sectional survey in 2015 and 2017 of energy drinks available in the main UK retailers.

Methods: The sugar (sugars g/100ml), energy (kcals/100ml), caffeine (mg/100ml) and serving size were collected from product packaging and nutrition information panels of energy drinks available in the nine main UK grocery retailers, three beauty and health retailers and one convenience store.

Results: The number of formulations (per 100ml) and number of products (per serving) have fallen (from 75 to 49 and from 90 to 59) between 2015 and 2017 respectively. Energy drinks surveyed showed a 10% reduction in sugar, from 10.6 to 9.5 g/100ml (p=0·011) and a 6% reduction in energy content (p=0·005) per 100ml between 2015 and 2017. The average caffeine content of energy drinks, with a warning label, has remained high at 31.5±0.9 in 2015 and 31·3±1.0 mg/100ml in 2017. Despite there being reductions, sugar, energy and caffeine content remain at concerning levels in 2017.

Conclusions: In order to reduce the harmful impact of energy drinks, further reduction in sugar and a reduction in caffeine by reformulation are urgently needed. Other measures such as ban on the sale of energy drinks to children and smaller product sizes should also be explored, while warning labels should be kept. A reduction in sugar, energy and caffeine content and overall energy drinks consumption could be beneficial in reducing sugar, energy and caffeine intake of consumers of energy drinks.

Strengths and limitations of this study

- This paper for the first time investigates and documents the sugar, energy and caffeine content of products marketed as energy drinks sold in the UK.

For peer review only - http://bmjopen.bmj.com/site/about/guidelines.xhtml

- These results demonstrate that the amount of sugar and energy has fallen, without technical issues, while caffeine levels remain high.
- This paper shows that serving sizes of energy drinks are large and should be reduced.
- The study was based on the levels provided on energy drinks packaging labels in store; hence we relied on the accuracy of the data provided on the label.

INTRODUCTION

In July 2015, the Scientific Advisory Committee on Nutrition (SACN) recommended that average free sugars (sugar) intake, across the UK population, should not exceed 5% of total energy intake. [1] This is in line with the World Health Organisation's conditional new guideline on sugar intake. [2, 3] SACN also advised that consumption of sugar-sweetened drinks, including energy drinks, should be minimised in children and adults, [1] because high intake of sugar is contributing to obesity, type 2 diabetes and dental caries, [3-11] major public health problems in the UK, [12-19] with significant healthcare costs. [20]

The average intakes in 2014 of sugar exceeded recommendations in all age groups. [21] The mean sugar intake in adults was 60g per day and contributes to 12% of daily energy intake. Children have a higher sugar intake with an average of 54g (13.4%) and 73g (15.1%) per day in 4-10 and 11-18 year olds respectively. [21]

Soft drinks are the main contributor of sugar intake in children (4-10 years) and teenagers (11-18 years) as well as the second main contributor in adults (18-64 years), contributing to 30%, 40% and 25% of sugar intake respectively. [21] Within soft drinks, energy drinks are a booming sub-category. [22-25]

Products described as 'energy drinks' by the industry are typically glucose based energy drinks; functional or stimulation energy drinks which claim a particular energy boost from caffeine, guarana, taurine, ginseng or other herbs or some combination of these ingredients, which are known to have stimulant properties and are distinct from sports drinks (which are often described as 'sports', 'isotonic' or 'hypotonic' and 'hypertonic'). [22, 26] Besides the health concerns around sugar content, the high levels of caffeine in some energy drinks are

For peer review only - http://bmjopen.bmj.com/site/about/guidelines.xhtml

associated with chronic sleep loss, addiction/dependence, withdrawal and intoxication. [26-29]

In the UK, sales of energy drinks have increased by 155% between 2006 and 2014, from 235 to 600 million litres, with an average per capita consumption of 9.4 litres in 2014. [30] The leading brands' shares in the UK energy drinks market, by value in 2013/14, were Red Bull (25%), own-label (12%), Monster (10%), Relentless (6%), Rockstar (5%), Mountain Dew (2%), Boost (2%) and Emerge (2%). [31]

Energy drinks consumption in the UK is a growing problem, particularly among children and teenagers. In 2011, a study by the European Food Safety Authority found young people in the UK consumed more energy drinks than in other EU countries (3.1 litres per person per month, compared to 2 litres) [32] and market research data suggest a potential increase in purchase among 10-14 year olds. [31]

The increase in consumption and popularity of energy drinks has raised concerns among the scientific community, governments and the general public about the health effects of these products, particularly among children and adolescents. [26, 33, 34] There have been two policies to tackle sugar and caffeine content of energy drinks. In terms of sugar content, a two-tiered Soft Drinks Industry Levy (SDIL) on the production and importation of sugar-sweetened drinks, including energy drinks, will be introduced in 2018. [35] The policy is intended to drive product reformulation and lower sugar consumption. Reformulation is commonly described as efforts by the manufacturer to lower the 'unhealthy' components (e.g. saturated fat, trans fats, sugar, salt) of food and drink product at the time of production, without worsening the profile of other ingredients (e.g. increasing calorie content). [36] The reformulated products become a replacement of an existing product (e.g. the same brand of energy drink with less sugar). The SDIL aims to incentivise manufacturers of sugar-sweetened drinks with more than 8g of sugar, per 100ml, to reduce the levels to below 8g and pay a lower tax rate and manufacturers of drinks with more than 5g of sugar per 100ml, to lower the sugar levels to less than 5g and pay no tax. [35]

For peer review only - http://bmjopen.bmj.com/site/about/guidelines.xhtml

In terms of caffeine content, the EU Food Information Regulation requires specific labelling for high caffeine drinks (over 150mg per litre). [37] The warning states that the product is not recommended for children. However, these products can be easily purchased and consumed by children.

This cross-sectional survey has been conducted to document levels of sugar and energy in energy drinks, as well as caffeine in the UK. The data available to monitor the energy drinks market, including whether reformulation is taking place, is generally the preserve of companies and not in the public domain. The main purpose of this research was to (a) report the variability in sugar and energy content in 2015 and 2017, (b) assess the sugar content in relation to the UK's new daily recommendation for sugar intake and by energy drinks brands, (c) quantify the levels of caffeine in energy drinks and determine the products on the market with the back of pack EU caffeine warning, (d) assess the number of products that will be taxed based on the criteria suggested in the SDIL [35] and (e) evaluate reformulation of energy drinks to reduce sugar and energy content between 2015 and 2017.

METHODS

The data was collected from product packaging and nutrient information panels in January 2015 and January 2017. The survey was designed as a comprehensive survey of all energy drinks available in a snapshot in time, using one large outlet for each of the nine main grocery retailers, three beauty and health retailers and one convenience store. This study used the same study design and procedures as reported in our previous work on carbonated sugar sweetened drinks, which excluded energy drinks. [38]

Definition

Energy drinks were defined as any drink products with 'energy', 'energise', 'energiser', 'caffeine' and 'stimulation' in the product name or description e.g. Red Bull Energy Drink, Monster Energy Drink, Relentless Origin Energy Drink and Tropicana Energy Mango and Guava with Passion fruit or products with high caffeine warning label including products not described as energy drink (such as Mountain Dew).

For peer review only - http://bmjopen.bmj.com/site/about/guidelines.xhtml

Since the focus of this study is on product reformulation, products labelled ‘zero’ or ‘light’ or ‘no added sugar’ etc. were excluded. We also excluded products described solely as ‘sports’ drinks, which consist primarily of carbohydrates and electrolytes and are intended for athletes to rehydrate after exercise e.g. ‘Lucozade Sports’.[22] However, ‘Lucozade Energy’ was included.

Data Collection

For each energy drink the data collected included the company name, product name, pack weight, serving size, sugars (g), energy (kcal) and caffeine (mg) per 100ml and per serving. Where data was not available per serving, it was calculated from pack size and per 100ml data. Caffeine content was only collected in a sub-sample of the 2015 products.

Where products only labelled carbohydrate content in 2015 but labelled sugar content in 2017 and the content of all nutrients and ingredients were the same, it was assumed that the sugar content from 2015 was the same as 2017 (applied to only one product).

The data were double checked after entry into excel spreadsheet, and a further 5% of entries were checked against the original source in a random selection of products by the lead author.

Stores

Data was collected from each of the major UK supermarkets in store (Aldi, Asda, Lidl, Marks and Spencer, Morrisons, Sainsbury’s, Tesco, The Co-operative and Waitrose), which altogether hold 93.2% of the grocery market share in the UK. [39] Three beauty, health and food retailers (Boots, Superdrug and Holland and Barrett) and one convenience store (Costcutter) were also included since they sell a wide variety of branded energy drinks.

Analysis

Per 100ml: Some brands sell the same formulation in different serving sizes. Therefore, the 100ml data only included an example of one formulation regardless of the different serving

For peer review only - http://bmjopen.bmj.com/site/about/guidelines.xhtml

For peer review only

sizes.

Per serving: The per serving data included all the different serving sizes available apart from 1 litre bottles. 1 litre products were excluded from the per serving analysis since it was deemed that the industry standardised serving of 250ml was too little and that consumers are likely to overconsume. Any product with up to 500ml can or bottle size was considered as one serving, regardless of what is stated on the packaging as a serving size e.g. often a 500ml bottle is split into two servings but we consider that most consumers drink these drinks as one serving.

Caffeine: Separate analysis was conducted on the products with the high caffeine warning label (over 150mg per litre).

High, Medium and Low criteria for sugars content: The sugar content was compared to the UK front of pack colour-coded labelling for drinks. Portion size criteria applied to portion/serving sizes greater than 150ml. Colour coding is based on the following front of pack colour-coded nutrition labelling criteria (Sugars – Red/High >13·5g/portion or >11·25g/100ml, Amber/Medium >2·5 to ≤11·25/100ml, Green/Low ≤2·25g/100ml). [40]

In addition to the above analyses, the sugar content was compared to the UK's recommendation for sugar intake for adults (30g/day) and 7-10 year old children (24g/day). [40] The highest energy containing products were compared to women's daily energy intake (2000 kcal). [41] Also, we assessed the number of products that will be taxed based on the criteria suggested in the SDIL. [35]

Statistical analysis

Comparison among products within each survey

Independent-Samples Mann-Whitney U Test was used to compare the levels of sugar between supermarket own label and branded products.

For peer review only - http://bmjopen.bmj.com/site/about/guidelines.xhtml

Comparison of the same products between the two years

For the purpose of assessing reformulation, only the products with data available in both surveys were included in this analysis. Paired t test was used to examine whether there was a significant change in the sugar and energy content of energy drinks from 2015 to 2017.

Data are reported as mean, SD, range as indicated. Significance in all tests carried out was deemed significant as being p<0·05. All data were analysed using SPSS Version 22.

RESULTS

Sugar, energy and caffeine content per 100ml in 2015 and 2017

A total of 75 and 49 energy drinks met the per 100ml inclusion criteria in 2015 and 2017 respectively. The average sugar content was 10.6±2.9 g and 9.7±3.0 g/100ml, with a large variation in sugar content between different energy drinks ranging from 1·9 to 15·9g and 2·1 to 16·0g/100ml in 2015 and 2017 respectively (Table 1). There were no significant differences in sugar content between supermarket own label and branded products in 2015 (P=0·397) and 2017 (P=0·245). The product with the highest and lowest sugar content per 100ml in 2015 and 2017 are highlighted in Table 2.

The average energy content in energy drinks was 47±13 kcal/100ml ranging from 10 to 70 kcal/100ml in 2015 and 44±13 kcal/100ml, ranging from 10 to 70 kcal/100ml in 2017 (Table 1). There were no significant differences in energy content between supermarket own label and branded products in 2015 (P=0·872) and 2017 (P=0·113).

Among all of the manufacturers with three or more formulations of energy drinks in 2015, Lucozade and Rockstar product ranges contained the highest average sugar and energy per 100ml respectively (Table 3). However, in 2017 the Rockstar product range contained the highest average sugar and energy content per 100ml (Table 3), which suggests Lucozade have been reformulating their products.

For peer review only - http://bmjopen.bmj.com/site/about/guidelines.xhtml

Since not all 'energy drinks' have a high caffeine content, we analysed the caffeine, sugar and energy content of the products with the warning label separately. A total of 22 and 40 energy drinks formulations had caffeine content labelled on pack in 2015 and 2017 (there may have been more that were labelled but not all were collected in 2015). The average caffeine content in energy drinks was 30.7±3.0 in 2015 and 31·7±0·7 mg/100ml in 2017 (Table 1). The levels of sugar, energy and caffeine were similar to the levels in the full sample of energy drinks.

Sugar, energy and caffeine content per serving in 2015 and 2017

A total of 90 and 59 energy drinks products met the inclusion criteria in 2015 and 2017 respectively (Table 4). The total number of products available has fallen between 2015 and 2017. The total number of products analysed per serving is different to the per 100ml data because some brands sell the same formulation in different serving sizes. The serving data included all the different serving sizes available apart from 1 litre bottles. The serving size varied from 150 to 500ml in 2015 and 250 to 500ml in 2017. 42% and 47% were sold in 500ml cans/bottles in 2015 and 2017 respectively.

The average sugar content in energy drinks was high (±SD) 41·3±17.9 in 2015 and 38.4±18.1g/serving in 2017 (Table 4), more than the entire maximum daily recommendation for sugar intake in the UK for an adult (30g). Indeed, 59% of products in 2015 and 54% in 2017 exceeded the maximum UK's recommendation for sugar intake per serving for an adult (30g/d). Additionally, 86% in 2015 and 78% in 2017 of products exceeded the maximum daily recommendation for sugar intake for a child aged 7-10 years (24g/d). 96% and 97% would receive a 'red' (high) label for sugars per serving (>13·5g/serving) in 2015 and 2017 respectively.

The serving size was significantly larger in branded verses supermarket own label products in 2015 (P=0·004) and 2017 (P=0·002), and as a result the branded products contained on average higher levels of sugar compared to supermarket products per serving in 2015 (P=0·028) and 2017 (P=0·027) (Figure 1 and Table 4).

For peer review only - http://bmjopen.bmj.com/site/about/guidelines.xhtml

The average energy content in energy drinks was 185±81kcal and 176±76/serving in 2015 and 2017 respectively. There was a large variation in energy content between different energy drinks ranging from 50 to 350 and 50 to 335kcal/serving in 2015 and 2017 respectively (Figure 2 and Table 4).

The energy drink products with the highest energy content (Lucozade Energy Original 500ml and Rockstar Punched Energy + Punch Fruit Punch Flavour 500ml) in 2015 and 2017 can contribute up to 17-17.5% of a woman's daily energy intake (2000 kcal).[41]

A total of 22 and 43 energy drinks formulations had high caffeine labels on pack (Table 4) in 2015 and 2017 respectively. The levels of sugar, energy and caffeine were similar to the full sample of energy drinks.

Soft Drink Industry Levy in 2017

A total of 36 products (69%) would be taxed at the higher sugar levy rate (>8g /100ml), 9 products (22%) at the lower rate (8-5g /100ml) and 4 products (9%) wouldn't be taxed (<5g /100ml). [35]

Comparison of the same products between the two years

The overall number of energy drinks formulations has decreased between 2015 and 2017, from 74 to 49 products.

Reformulation

There were 30 products surveyed repeatedly in 2015 and 2017. The average sugar content per 100ml for these 30 products was 10·6±3·2g in 2015 and 9·5±3·3g in 2017. This represents a reduction of 10% (p=0·011). The sugar content has reduced in 12 products, remained the same in 16 and has increased in 2. The average energy content per 100ml was 47±14 kcal in 2015 and 44±15 kcal in 2017, representing a 6% reduction (p=0·005).

There were 12 products surveyed repeatedly in 2015 (31.5±0.9) and 2017 (31·3±1.0 mg/100ml) displaying the high caffeine warning label. The caffeine content of these

For peer review only - http://bmjopen.bmj.com/site/about/guidelines.xhtml

products stayed the same, apart from one product, where the caffeine content was reduced, from 32mg to 30mg.

Note that these averages are slightly different from those when all products were included in each year and this trend analysis reflects reductions made in the same products rather than the overall products available, giving a better reflection of reformulation, for a full list of products refer to supplementary file 1.

DISCUSSION

This survey has documented the levels of sugar, energy and caffeine in energy drinks in the UK between 2015 and 2017, a topic which hasn't been previously documented for easy access by researchers and policy-makers. It will allow for evaluation of trends in the energy drinks market in the future.

The surveys have shown early changes in the energy drinks market. Formulations (per 100ml) and number of products (per serving) have fallen between 2015 and 2017. This may be due to the pressure on the soft drinks industry to reduce sugary drinks offerings. [42] Furthermore, the energy drinks surveyed showed a small but statistically significant reduction in sugar content; this is likely due to the 12 products which have reduced sugar content between 2015 and 2017, probably as a result of reformulation due to the impending SDIL.[35] Manufacturers of the reduced products have either only reduced sugar or have alternatively reduced sugar and replaced it with non-caloric sweeteners without changing the product name e.g. by calling the product 'light' etc. Given the volume consumed, [22] even small reductions in sugar content of energy drinks would have a significant impact on sugar and energy intake among regular consumers of energy drinks. [43]

This survey may suggest the early success of the impending SDIL. Even though the SDIL is yet to come in, manufacturers have started to reformulate their products in advance, in order to avoid paying the levy. However, we are yet to see if the SDIL will have an impact on

For peer review only - http://bmjopen.bmj.com/site/about/guidelines.xhtml

overall consumption of energy drinks by reducing sales. [44] Other countries have imposed taxes on soft drinks, including energy drinks, and have seen reductions in sales.[45] [46]

Despite this, sugar content remains at concerning levels, 97% would receive a 'red' (high) label for sugars per serving in 2017 respectively. There was also a large variation in sugar content between different energy drinks ranging from 2·1 to 16.0g/100ml, which suggests further reductions, are possible.

As well as the concerns around the sugar and energy content of energy drinks, there are also concerns about caffeine levels, particularly in the products labelled as high caffeine. In 2017, average caffeine content in energy drinks was 124.3 ± 39.2 mg/serving among the 43 products labelled, almost equivalent to two cups of coffee or four cans of cola.[37] There is also some evidence to suggest that the caffeine content increases sugar-sweetened drinks consumption further and therefore sugar intake too.[47] Since children and teenagers are the main consumers of energy drinks, manufacturers should consider reducing levels of caffeine - again through reformulation. The removal of caffeine also allows for the removal of 10·3% of sugar without affecting taste, which has the potential to reduce body weight of adults by 0.6kg, without any change in sugar-sweetened drinks consumption. [48]

It important to note that not all manufacturers label the amount of caffeine on their energy drinks and this is likely because their products do not exceed the cut-off for adding the warning label.[37] Among the manufacturers that do, a warning is stated on the pack, acknowledging the potential danger these products can pose to children. However, these products can be easily purchased and consumed by children. Perhaps the UK should consider restrictions like other countries have. In Lithuania, sales of energy drinks (containing at least 150mg of caffeine per litre) are banned to under-18s. [49] A similar law applies in Sweden where sales energy drinks are banned to under-15s and sales of some energy drinks are restricted to pharmacies.[26]

Furthermore, serving sizes of energy drinks matter since the larger the serving size, the more sugar, energy and caffeine is consumed. Indeed, typical serving sizes of energy drinks

For peer review only - http://bmjopen.bmj.com/site/about/guidelines.xhtml

are larger than other sugar-sweetened drinks; [38] this survey showed that the most common serving size was 500ml. The average sugar content in energy drink in both surveys per serving was more than an adult's entire maximum daily recommendation for sugar intake in the UK. Likewise, 86% in 2015 and 78% in 2017 of the products exceeded the maximum daily recommendation for sugar intake for a child aged 7-10 years (24g/d). [1] The products with the highest sugar and energy content per serving could contribute up 17.5% of a woman's total daily energy intake. Therefore, in order to reduce the amount of sugar, energy and caffeine consumed from energy drinks, there is scope for introducing restrictions on larger serving sizes (500ml bottles and cans). This is a growing problem, which must be tackled, particularly since the Grocer recently reported an increase in sales of larger cans of energy drinks on the UK market. [23]

Our study was based on sugar, energy and caffeine content data provided on the available energy drinks packaging labels in store on the dates of collection; hence we relied on the accuracy of the data provided on the label and the availability of products in store. It is assumed that the manufacturers provide accurate and up to date information in line with EU Regulations. However, future studies should include sugar and energy content determined through laboratory analysis to achieve a better understanding of the true sugar and energy content, and breakdown of the types of sugars. Furthermore, an analysis of what are the best selling products in the UK would allow for an accurate estimate of the potential compact of reformulation.

Also, the data collection process of caffeine content was slightly different in the two surveys. In 2015, caffeine was collected in a sub-sample of products, since the aim was not to collect caffeine at the time, but the data that was collected was included in the analysis, this may not give a full reflection of caffeine content of all products on the market in 2015.

Still, the results of this study are relevant and serve to document the sugar, energy and caffeine content of energy drinks sold the in UK, underpinning future studies and providing evidence for the Treasury and the soft drinks industry, who are reformulating. [50, 51]

For peer review only - http://bmjopen.bmj.com/site/about/guidelines.xhtml

CONCLUSION

Factors such as an increase in sales, [24, 25, 30] concerning levels of sugar, energy and caffeine (assessed in this study), ease of access [33], large serving sizes, as well as limited regulation have created an environment where energy drinks could pose a major threat to public health.

In order to reduce the impact of energy drinks, sugar (and therefore energy) reformulation should continue and begin regarding caffeine content. Other measures such as a ban on the sale of energy drinks to children should be explored, while warning labels should be kept. Can and bottle sizes of energy drinks should also be decreased to reduce the overall consumption of sugar, energy and caffeine. A reduction in sugar, energy and caffeine content and overall energy drinks consumption could be beneficial in reducing sugar, energy and caffeine intake of consumers of energy drinks.

COMPETING INTERESTS

All authors have completed the ICMJE uniform disclosure form at www.icmje.org/coi_disclosure.pdf (available on request from the corresponding author) and declare: no support from any organisation for the submitted work; no financial relationships with any organisations that might have an interest in the submitted work in the previous three years. KH is an employee of Consensus Action on Salt, Sugar & Health (CASSH), a nonprofit charitable organisation. FH is a member of Consensus Action on Salt and Health (CASH) and its international branch World Action on Salt & Health (WASH) and does not receive any financial support from CASH or WASH. MacGregor GA is Chairman of Blood Pressure UK (BPUK), Chairman of CASSH and Chairman of WASH. BPUK, CASSH and WASH are nonprofit charitable organisations. GM does not receive any financial support from any of these organisations.

CONTRUBUTORSHIP

KH conducted research; KH and FH analysed data; KH wrote the first draft of the manuscript and all authors contributed to the interpretation of the results and revision of the manuscript, and approved the final manuscript. GM had primary responsibility for final

For peer review only - http://bmjopen.bmj.com/site/about/guidelines.xhtml

content.

FUNDING

This research received no specific grant from any funding agency in the public, commercial or not-for-profit sectors.

DATA SHARING STATEMENT

Extra data is available by emailing Kawther Hashem on k.hashem@qmul.ac.uk.

REFERENCE

1. PHE. Why 5%? 2015 [1st July 2017]. Available from: https://www.gov.uk/government/uploads/system/uploads/attachment_data/file/446010/Why_5___-_The_Science_Behind_SACN.pdf.
2. WHO. Guideline: Sugars intake for adults and children 2015 [updated 1 July 2015]. Available from: http://apps.who.int/iris/bitstream/10665/149782/1/9789241549028_eng.pdf?ua=1.
3. Moynihan PJ, Kelly SA. Effect on Caries of Restricting Sugars Intake: Systematic Review to Inform WHO Guidelines. *J Dent Res*. 2014;93:8-18.
4. SACN. Carbohydrates and Health 2015 [updated 1 December 2015]. Available from: https://www.gov.uk/government/uploads/system/uploads/attachment_data/file/445503/SACN_Carbohydrates_and_Health.pdf.
5. Romaguera D, Norat T, Wark PA, et al. Consumption of sweet beverages and type 2 diabetes incidence in European adults: results from EPIC-InterAct. *Diabetologia*. 2013;56:1520-30.
6. de Koning L, Malik VS, Rimm EB, et al. Sugar-sweetened and artificially sweetened beverage consumption and risk of type 2 diabetes in men. *The American journal of clinical nutrition*. 2011;93:1321-7.
7. Maki KC, Phillips AK. Dietary substitutions for refined carbohydrate that show promise for reducing risk of type 2 diabetes in men and women. *J Nutr*. 2015;145:159S-63S.
8. Feinman RD, Pogozelski WK, Astrup A, et al. Dietary carbohydrate restriction as the first approach in diabetes management: Critical review and evidence base. *Nutrition*. 2015;31:1-13.
9. Te Morenga L, Mallard S, Mann J. Dietary sugars and body weight: systematic review and meta-analyses of randomised controlled trials and cohort studies. *Bmj*. 2013;346:e7492.
10. Johnson RK, Appel LJ, Brands M, et al. Dietary sugars intake and cardiovascular health: a scientific statement from the American Heart Association. *Circulation*. 2009;120:1011-20.
11. Xi B, Li SS, Liu ZL, et al. Intake of Fruit Juice and Incidence of Type 2 Diabetes: A Systematic Review and Meta-Analysis. *PloS one*. 2014;9:e93471.
12. PHE. UK and Ireland prevalence and trends.2013 2 July 2015. Available from: https://www.noo.org.uk/NOO_about_obesity/adult_obesity/UK_prevalence_and_trends.
13. HSCIC. Statistics on Obesity, Physical Activity and Diet: England 20142014 July 2015. Available from: http://www.hscic.gov.uk/catalogue/PUB13648/Obes-phys-acti-diet-eng-2014-rep.pdf.
14. PHE. Adult obesity and type 2 diabetes2014 2 July 2015. Available from: https://www.gov.uk/government/uploads/system/uploads/attachment_data/file/338934/Adult_obesity_and_type_2_diabetes_.pdf.

For peer review only - http://bmjopen.bmj.com/site/about/guidelines.xhtml

15. HSCIC. National Diabetes Audit2008 July 2015. Available from: http://www.hscic.gov.uk/catalogue/PUB02580/nati-diab-audi-07-08-exec-summ.pdf.
16. Houses-of-Parliament. Sugar and Health. POSTNOTE: 4932015 2 December 2015. Available from: http://www.actiononsalt.org.uk/actiononsugar/Sugar%20in%20the%20news/2015/159388.pdf.
17. PHE. National Dental Epidemiology Programme for England: oral health survey of five-year-old children 2012. A report on the prevalence and severity of dental decay2012 2 December 2015. Available from: http://www.nwph.net/dentalhealth/Oral%20Health%205yr%20old%20children%202012%20final%20report%20gateway%20approved.pdf.
18. PHE. Dental public health epidemiology programme. Oral health survey of three-year-old children 2013. A report on the prevalence and severity of dental decay2014 2 December 2015. Available from: http://www.nwph.net/dentalhealth/reports/DPHEP%20for%20England%20OH%20Survey%203yr%202013%20Report.pdf.
19. NHS. Executive Summary: Adult Dental Health Survey2009 2 December 2015. Available from: http://www.hscic.gov.uk/catalogue/PUB01086/adul-dent-heal-surv-summ-them-exec-2009-rep2.pdf.
20. PHE. Sugar reduction Responding to the challenge 2014 [updated 1 June 2014]. Available from: https://www.gov.uk/government/uploads/system/uploads/attachment_data/file/324043/Sugar_Reduction_Responding_to_the_Challenge_26_June.pdf.
21. PHE. NDNS: results from Years 5 and 6 (combined) 2016 [updated 2 June 2016]. Available from: https://www.gov.uk/government/statistics/ndns-results-from-years-5-and-6-combined.
22. BSDA. Creating New Choices. The 2014 UK Soft Drinks Report2014 23 June 2015. Available from: http://www.britishsoftdrinks.com/write/MediaUploads/Publications/Revised_BSDA_Annual_Report_2014.pdf.
23. The-Grocer. Red Bull flies high as full-sugar sales surge 2017 [30 July 2017]. Available from: https://www.thegrocer.co.uk/buying-and-supplying/health/red-bull-flies-high-as-full-sugar-sales-surge/550194.article.
24. Kleiman S, Ng SW, Popkin B. Drinking to our health: can beverage companies cut calories while maintaining profits? *Obesity reviews : an official journal of the International Association for the Study of Obesity*. 2012;13:258-74.
25. Grocer. T. Focus on energy drinks 2015 2015 [25 August 2016]. Available from: http://www.thegrocer.co.uk/reports/category-reports/focus-on-energy-drinks-2015/520323.article.
26. Breda JJ, Whiting SH, Encarnação R, et al. Energy drink consumption in Europe: A review of the risks, adverse health effects and policy options to respond. *Frontiers in Public Health*. 2014;2.
27. Owens J. Insufficient Sleep in Adolescents and Young Adults: An Update on Causes and Consequences. *Pediatrics*. 2014;134:e921-e32.
28. Seifert SM, Schaechter JL, Hershorin ER, et al. Health Effects of Energy Drinks on Children, Adolescents, and Young Adults. *Pediatrics*. 2011.
29. Reissig CJ, Strain EC, Griffiths RR. Caffeinated Energy Drinks -- A Growing Problem. *Drug and alcohol dependence*. 2009;99:1-10.
30. BSDA. Changing Tastes. The UK Soft Drinks Annual Report 2015. In: Association BSD, editor. 2015.
31. Mintel. Sports and Energy Drinks - UK - July 2014 2014.
32. Consortium N-A. Gathering consumption data on specific consumer groups of energy drinks2013 20 March 2017. Available from: http://onlinelibrary.wiley.com/doi/10.2903/sp.efsa.2013.EN-394/pdf.

For peer review only - http://bmjopen.bmj.com/site/about/guidelines.xhtml

33. Visram S, Hashem, K,. Energy drinks: what's the evidence? *Food Research Collaboration* 2016.
34. European-Parliament. Schaldemose: "Energy drinks shouldn't have any kind of health claims on them" 2016 [1 September]. Available from: http://www.europarl.europa.eu/news/en/news-room/20160613STO31976/schaldemose-energy-drinks-shouldn't-have-any-kind-of-health-claims-on-them.
35. Gov.uk. Policy paper Soft Drinks Industry Levy 2016 [July 2017]. Available from: https://www.gov.uk/government/publications/soft-drinks-industry-levy/soft-drinks-industry-levy.
36. van Raaij J, Hendriksen M, Verhagen H. Potential for improvement of population diet through reformulation of commonly eaten foods. *Public health nutrition*. 2009;12:325-30.
37. FSA. High caffeine 'energy' drinks and other foods containing caffeine n.d [1 July 2017]. Available from: https://www.food.gov.uk/science/additives/energydrinks.
38. Hashem KM, He FJ, Jenner KH, et al. Cross-sectional survey of the amount of free sugars and calories in carbonated sugar-sweetened beverages on sale in the UK. *BMJ open*. 2016;6.
39. Kantar-Worldpanel. Grocery Market Share - Kantar Worldpanel2016 30th April 2016. Available from: http://www.kantarworldpanel.com/en/grocery-market-share/great-britain.
40. Department-of-Health. Guide to creating a front of pack (FoP) nutrition label for pre-packed products sold through retail outlets.2016 1 July 2017. Available from: https://www.food.gov.uk/sites/default/files/multimedia/pdfs/pdf-ni/fop-guidance.pdf.
41. NHS. What should my daily intake of calories be? 2016 [1 July 2017]. Available from: http://www.nhs.uk/chq/pages/1126.aspx?categoryid=51.
42. PHE. Sugar Reduction The evidence for action 2015 2 October 2015 Available from: https://www.gov.uk/government/uploads/system/uploads/attachment_data/file/470179/Sugar_reduction_The_evidence_for_action.pdf.
43. Ma Y, He FJ, Yin Y, et al. Gradual reduction of sugar in soft drinks without substitution as a strategy to reduce overweight, obesity, and type 2 diabetes: a modelling study. *The Lancet Diabetes & Endocrinology*. 2016;4:105-14.
44. Briggs ADM, Mytton OT, Kehlbacher A, et al. Health impact assessment of the UK soft drinks industry levy: a comparative risk assessment modelling study. *The Lancet Public Health*.2:e15-e22.
45. WCRF. Use economic tools to address food affordability and purchase incentives 2016 [11 August]. Available from: http://www.wcrf.org/int/policy/nourishing-framework/use-economic-tools.
46. Colchero MA, Popkin BM, Rivera JA, et al. Beverage purchases from stores in Mexico under the excise tax on sugar sweetened beverages: observational study. *Bmj*. 2016;352.
47. Keast RSJ, Swinburn BA, Sayompark D, et al. Caffeine increases sugar-sweetened beverage consumption in a free-living population: a randomised controlled trial. *The British Journal of Nutrition*. 2015;113:366-71.
48. Keast RS, Sayompark D, Sacks G, et al. The influence of caffeine on energy content of sugar-sweetened beverages: 'the caffeine-calorie effect'. *European journal of clinical nutrition*. 2011;65:1338-44.
49. Law. EF. Lithuania bans sale of energy drinks to children 2014. Available from: http://www.eurofoodlaw.com/country-reports/eu-member-states/lithuania/lithuania-bans-sale-of-energy-drinks-to-children-104098.htm.
50. Lucozade-Ribena-Suntory. Health and Wellbeing n.d. [1st July 2017]. Available from: https://www.lrsuntory.com/health-and-wellbeing/.
51. Tesco. Tesco reduces sugar content in all own brand soft drinks 2016 [1st July 2017]. Available from: https://www.tescoplc.com/news/news-releases/2016/tesco-reduces-sugar-content-in-all-own-brand-soft-drinks.

For peer review only - http://bmjopen.bmj.com/site/about/guidelines.xhtml

Legend for figure 1: Sugar content in supermarket and branded energy drinks (g/serving) in 2017

Legend for figure 2: Energy content in supermarket and branded energy drinks (g/serving) in 2017

For peer review only

For peer review only - http://bmjopen.bmj.com/site/about/guidelines.xhtml

Table 1: Sugar, energy and caffeine content in energy drinks per 100ml

	2015				2017			
**Mean±SD (range)**	**N (caffeine)**	**sugar (g)**	**Energy (kcal)**	**Caffeine (mg)**	**N (caffeine)**	**Sugar (g)**	**Energy (kcal)**	**Caffeine (mg)**
**All**								
**Own label**	13 (5)	10.8 ± 1.6 (9.6 - 15.9)	48 ± 6 (44 - 67)	30.4 ± 0.9 (30.0 - 32.0)	9 (9)	9.1 ± 2.3 (4.9 - 12.8)	39 ± 8 (24 - 52)	30.9 ± 1.1 (30.0 - 32.0)
**Branded**	62 (19)	10.5 ± 3.1 (1.9 - 15.6)	47 ± 14 (10 - 70)	29.2 ± 7.8 (0.0 - 32.0)	40 (30)	9.8 ± 3.1 (2.1 - 16.0)	45 ± 13 (10 - 70)	31.7 ± 0.6 (30.0 - 32.0)
**Total**	75 (24)	10.6 ± 2.9 (1.9 - 15.9)	47 ± 13 (10 - 70)	29.4 ± 6.9 (0.0 - 32.0)	49 (39)	9.7 ± 3.0 (2.1 - 16.0)	44 ± 13 (10 - 70)	31.5 ± 0.8 (30.0 - 32.0)
**Labelled high caffeine**								
**Own label**	4	10.2 ± 0.6 (9.8 - 10.9)	45 ± 2 (44 - 48)	30.5 ± 1.0 (30.0 - 32.0)	7	9.5 ± 2.5 (4.9 - 12.8)	40 ± 9 (24 - 52)	31.1 ± 1.1 (30.0 - 32.0)
**Branded**	18	11.1 ± 3.3 (3.0 - 15.6)	49 ± 12 (17 - 67)	30.7 ± 3.3 (18.0 - 32.0)	33	10.6 ± 2.8 (2.1 - 16.0)	45 ± 11 (10 - 67)	31.8 ± 0.6 (30.0 - 32.0)
**Total**	22	11.0 ± 3.0 (3.0 - 15.6)	48 ± 11 (17 - 67)	30.7 ± 3.0 (18.0 - 32.0)	40	10.4 ± 2.8 (2.1 - 16.0)	44 ± 11 (10 - 67)	31.7 ± 0.7 (30.0 - 32.0)

Table 2: Highest and lowest sugar energy drink per 100ml in 2015 and 2017

	2015		2017	
	**Product name**	**Sugar (g)**	**Product name**	**Sugar (g)**
**Highest**	Sainsbury's Orange Energy Drink	15.9	AG Barr - Rockstar Punched Energy + Guava Tropical Guava Flavour	16.0
**Lowest**	Monster Rehab Green Tea Energy	1.9	Monster Rehab Tea + Still Lemonade + Energy	2.7

For peer review only - http://bmjopen.bmj.com/site/about/guidelines.xhtml

Table 3. Sugar and energy content in energy drinks by manufacturer per 100ml in 2017

2015			2017		
**Manufacturer**	**N**	**Sugar (g) mean±SD (range)**	**Manufacturer**	**N**	**Sugar (g) mean±SD (range)**
Rockstar	8	14.1 ± 1.1 (12.1 - 15.6)	Rockstar	5	14.2 ± 1.5 (12.0 - 16.0)
Lucozade	10	12.5 ± 1.5 (8.7 - 14.0)	Relentless	5	11.1 ± 2.4 (7.5 - 14.0)
Sainsbury's	4	12.1 ± 2.6 (10.6 - 15.9)	Red Bull	3	11.0 ± 0.0 (11.0 - 11.0)
Relentless	5	10.6 ± 0.6 (10.1 - 11.6)	Lidl	5	10.2 ± 1.7 (8.8 - 12.8)
Asda	3	10.5 ± 0.2 (10.4 - 10.7)	Lucozade	6	8.6 ± 3.4 (4.3 - 13.0)
KX	3	10.2 ± 0.2 (10.1 - 10.5)	Monster	8	8.5 ± 2.8 (2.1 - 11.0)
Little Miracles	3	6.4 ± 0.2 (6.2 - 6.6)	Little Miracles	3	5.7 ± 0.5 (5.1 - 6.1)
Monster	7	6.4 ± 4.2 (1.9 - 11.0)			
**Manufacturer**	**N**	**Energy (kcal) mean±SD (range)**	**Manufacturer**	**N**	**Energy (kcal) mean±SD (range)**
Lucozade	10	63.0 ± 4.8 (57.0 - 70.0)	Rockstar	5	60.0 ± 6.6 (51.0 - 67.0)
Rockstar	8	59.9 ± 5.4 (52.0 - 67.0)	Lucozade	6	51.5 ± 14.8 (35.0 - 70.0)
Sainsbury's	4	53.0 ± 9.3 (48.0 - 67.0)	Red Bull	3	45.7 ± 0.6 (45.0 - 46.0)
Asda	4	10.3 ± 0.5 (9.6 - 10.7)	Relentless	5	45.6 ± 9.7 (31.0 - 58.0)
Relentless	5	44.4 ± 2.1 (43.0 - 48.0)	Lidl	5	42.4 ± 6.7 (36.0 - 52.0)
KX	3	44.0 ± 2.0 (42.0 - 46.0)	Monster	8	37.0 ± 12.0 (10.0 - 48.0)
Monster	7	28.6 ± 17.7 (10.0 - 48.0)	Little Miracles	3	24.7 ± 1.5 (23.0 - 26.0)
Little Miracles	3	26.7 ± 0.6 (26.0 - 27.0)			

For peer review only

For peer review only - http://bmjopen.bmj.com/site/about/guidelines.xhtml

Table 4: Sugar, energy and caffeine content in energy drinks per serving

Mean±SD (range)	2015 N (caffeine)	2015 Sugar (g)	2015 Energy (kcal)	2015 Caffeine (mg)	2017 N (caffeine)	2017 Sugar (g)	2017 Energy (kcal)	2017 Caffeine (mg)
**All**								
**Own label**	12 (4)	30.8 ± 11.1 (23.9 - 54.5)	137 ± 49 (110 - 245)	76.2 ± 2.5 (75.0 - 80.0)	8 (8)	27.3 ± 15.6 (12.3 - 64.0)	116 ± 61 (60 - 260)	87.5 ± 29.4 (75.0 - 160.0)
**Branded**	78 (19)	42.7 ± 18.3 (9.5 - 78.0)	193 ± 83 (50 - 350)	115.7 ± 47.1 (0.0 - 160.0)	51 (34)	40.2 ± 18.0 (9.9 - 80.0)	185 ± 75 (50 - 335)	134.2 ± 36.0 (75.0 - 160.0)
**Total**	90 (23)	41.3 ± 17.9 (9.5 - 78.0)	185 ± 81 (50 - 350)	108.9 ± 45.3 (0.0 - 160.0)	59 (43)	38.4 ± 18.1 (9.9 - 80.0)	176 ± 76 (50 - 335)	124.3 ± 39.3 (75.0 - 160.0)
**Labelled high caffeine**								
**Own label**	4	25.5 ± 1.6 (23.9 - 27.3)	113 ± 6 (107 - 120)	76.3 ± 2.5 (75.0 - 80.0)	8	27.3 ± 15.6 (12.3 - 64.0)	116 ± 61 (60 - 260)	87.5 ± 29.4 (75.0 - 160.0)
**Branded**	18	46.1 ± 22.4 (9.9 - 78.0)	199 ± 89 (56 - 335)	122.1 ± 40.0 (75.0 - 160.0)	35	44.8 ± 18.0 (9.9 - 80.0)	191 ± 75 (50 - 335)	132.7 ± 36.6 (75.0 - 160.0)
**Total**	22	42.3 ± 21.7 (9.9 - 78.0)	183 ± 87 (56 - 335)	113.8 ± 39.5 (75.0 - 160.0)	43	41.4 ± 18.7 (9.9 - 80.0)	177 ± 78 (50 - 335)	124.3 ± 39.3 (75.0 - 160.0)

For peer review only - http://bmjopen.bmj.com/site/about/guidelines.xhtml

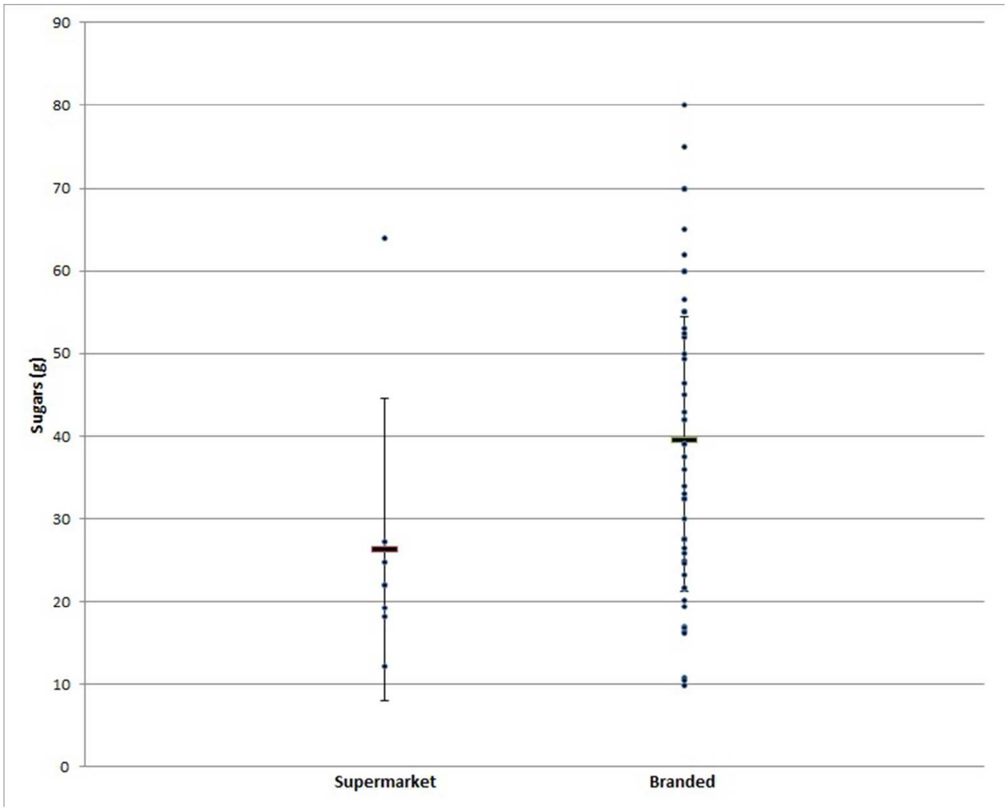

figure 1: Free sugars content in supermarket and branded energy drinks (g/serving) in 2017

66x53mm (300 x 300 DPI)

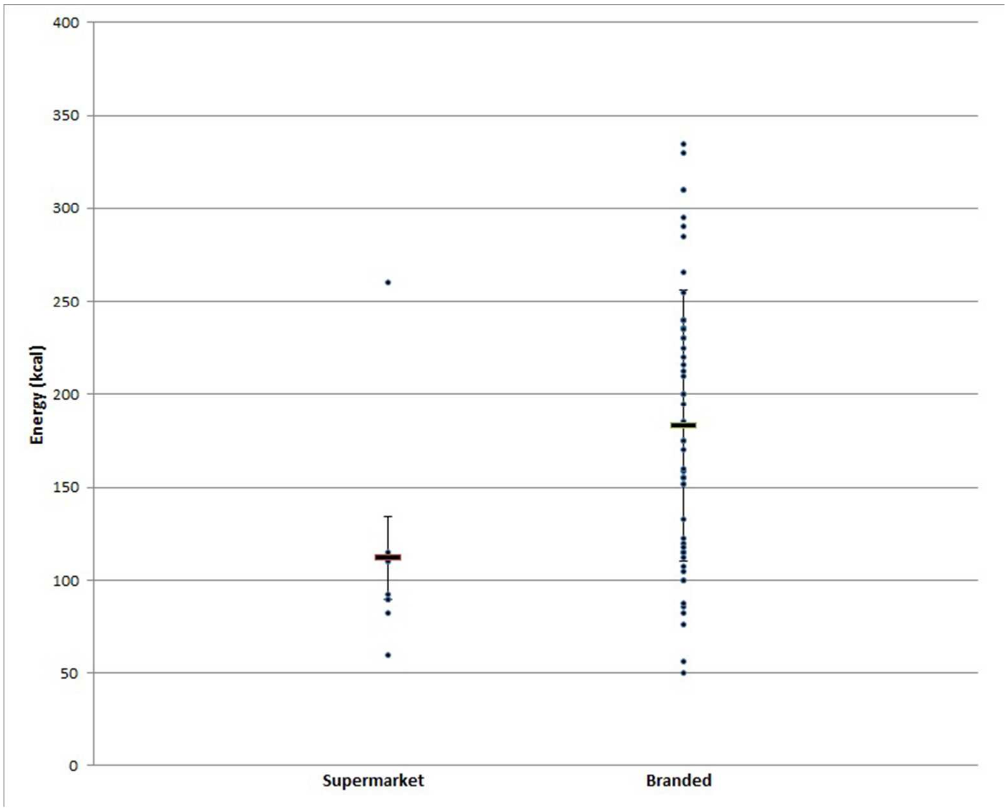

figure 2: Calorie content in supermarket and branded energy drinks (g/serving) in 2017

66x53mm (300 x 300 DPI)

w only

NA - product did not exist in 2015, so caffeine data collection was not applicable
NC - not collected and caffeine label may have not been available
NL - no caffeine warning label

Brand Name	Product Name	Pack Size (ml)	Energy (kcal) per 100ml 2015	Energy (kcal) per 100ml 2017	Sugars (g) per 100ml 2015	Sugars (g) per 100ml 2017	Caffeine (mg) per 100ml 2015	Caffeine (mg) per 100ml 2017
PepsiCo	Tropicana Energy Mango and Guava with Passionfruit 150ml	150	53		10.9		NC	NA
PepsiCo	Tropicana Energy Pineapple, Mango and Banana 150ml	150	49		9.8		NC	NA
Tesco	Tesco Blue Spark 6 x 250ml	250	44	24	9.8	4.9	32.0	30.0
Lidl	Lidl Freeway Up Tropical Juiced Energy Drink 250ml	250	46	36	10.5	8.8	NC	32.0
Lidl	Lidl Freeway Up Berry Juiced Energy Drink 250ml	250		36		8.8	NA	32.0
Asda	Asda Chosen by You Original Blue Charge 250ml	250	48	37	10.7	7.7	NC	30.0
Asda	Asda Chosen by You Chai Spice Flavour Blue Charge 250ml	250	43		9.6		30.0	NA
Lucozade	Lucozade Energy Pink Lemonade 250ml	250		40		6.8	NA	NL
Cott	Emerge Energy Drink Original 250ml	250	42	42	9.8	9.3	30.0	30.0
Scheckter's	Scheckter's Organic Energy 250ml	250	47	43	10.8	10	NC	32.0
Lidl	Lidl Freeway Up Lime Energy Drink 250ml	250		44		10.9	NA	32.0
Lidl	Lidl Freeway Up Classic Stimulation Drink 250ml	250		44		9.9	NA	30.0
Red Bull	Red Bull Energy Drink The Orange edition 250ml	250		45		11	NA	32.0
V	V Guarana Energy Drink 250ml	250	45	45	11.2	11	NC	31.0
Morrisons	Morrisons Source Orange and Passion Fruit Energy Drink 250ml	250	46	46	10.6	10.6	30.0	30.0
Red Bull	Red Bull Energy Drink 250ml	250	46	46	11.0	11	NC	32.0
Dico	MTV UP! Energy Drink Classic 250ml	250		46		11	NA	32.0
Red Bull	Red Bull Energy Drink The Tropical Edition 250ml	250		46		11	NA	32.0
Hergesteilt	Seagull Energy Drink 250ml	250		46		11	NA	32.0
Boost	Boost Energy Original 250ml	250	47	47	10.6	10.6	NC	30.0
Sun Mark	Bulldog Power Energy Drink 250ml	250		49		11	NA	31.5
Lucozade	Lucozade Energy Orange 250ml	250	62	62	12.5	13	NC	NL
Sainsbury's	Sainsbury's Blue Bolt Cherry & Blackcurrant Energy Drink 250ml	250	48		10.9		30.0	NA
Sainsbury's	Sainsbury's Blue Bolt Energy Drink 250ml	250	49		10.9		NC	NA
Sainsbury's	Sainsbury's Blue Bolt Mango & Passion Fruit Energy Drink 250ml	250	48		10.6		NC	NA
Asda	Asda Chosen by You Orange Twist Blue Charge Energy Drink 250ml	250	46		10.4		NC	NA
Waitrose	Waitrose Love Life Energy Release Drink 250ml	250	47		10.4		NC	NA
Big Up	Big Up Power Fruit Punch Energy Drink 250ml	250	52		12.2		30.0	NA
Big Up	Big Up Power Wild Ginger Energy Drink 250ml	250	51		12.1		30.0	NA
SoBe	SoBe Pure Rush Energy Drink Appleberry Burst 250ml	250	54		12.0		NC	NA
SoBe	SoBe Pure Rush Energy Drink Tropical Twist 250ml	250	54		12.0		NC	NA
Black	Black Mojito Energy Drink 250ml	250	47		11.3		NC	NA
Tiger	Tiger Energy Drink 250ml	250	46		10.9		NC	NA
Black	Black Energy Drink 250ml	250	46		10.8		NC	NA
Singh is King	Singh is King Energy Drink India's Energy Drink 250ml	250	48		10.8		30.0	NA
KX	KX Energy Stimulation Drink 250ml	250	44		10.1		32.0	NA
KX	KX Red Berry Stimulation Drink 250ml	250	42		10.1		NC	NA
Relentless	Relentless Origin Energy Drink 250ml	250	43		10.1		NC	NA
EQ8	EQ8 Natural Energy Drink Orange & Passion Fruit 250ml	250	40		9.3		NC	NA
EQ8	EQ8 Natural Energy Drink Cranberry & Apple 250ml	250	29		6.3		NC	NA
Organiq	Organiq Natural Energy Drink 269ml	269	42		5.6		32.0	NA
Atlantic Multipower	Multipower Red Kick 330ml	330	17	17	3	3	32.0	32.0
Little Miracles	Little Miracles Organic Energiser Lemongrass, Orange, Ginger, Ginseng, Agave	330		23		5.1	NA	NL
Little Miracles	Organic Energy Green Tea, Ginseng, Pomegranate, Acai, Acave 330ml	330	26	25	6.2	5.9	NL	NL
Little Miracles	Organic Energy White Tea, Ginseng, Cherry, Acai, Agave 330ml	330	27	26	6.3	6.1	NL	NL
Effect	Effect the Mental Energizer 330ml	330	45		10.7		32.0	NA
Little BigShot	Little BigShot Energy Mixed Berry 330ml - without caffeine!	330	36		9.0		0.0	NA
Little Miracles	Organic Energy Black Tea, Ginseng, Peach, Acai, Agave 330ml	330	27		6.6		NL	NA
Red Bull	Red Bull Energy Drink 355ml	355	46	45	11.0	11	NC	32.0
Innocent	Innocent Super Smoothie Energise 360ml	360	62	60	14.0	10	NL	NL
Powerade	Powerade Energy Sparkling Berry 375ml	375	44		10.5		NC	NA
Lucozade	Lucozade Energy Tropical Fusion Pineapple & Kiwi Flavour 6 x 380ml	380		35		4.3	NA	NL
Lucozade	Lucozade Energy Pink Lemonade 380ml	380	57	40	14.0	6.8	NC	NL
Lucozade	Lucozade Energy Caribbean Crush 380ml	380	57	40	14.0	6.5	NC	NL
Lucozade	Lucozade Energy Orange 380ml	380	62	62	12.5	13	NC	NL
Lucozade	Lucozade Energy Original 380ml	380	70	70	8.7	8.7	NC	NL
Lucozade	Lucozade Energy Apple 380ml	380	68		13.4		NC	NA
Lucozade	Lucozade Energy Tropical 380ml	380	70		13.0		NC	NA
Lucozade	Lucozade Energy Limited Edition Melonade 380ml	380	62		12.4		NC	NA
Lucozade	Lucozade Energy Cherry 380ml	380	61		12.3		NC	NA
Lucozade	Lucozade Energy Blackcurrant 380ml	380	61		12.2		NC	NA
Red Bull	Red Bull Energy Drink 473ml	473	46	45	11.0	11	32.0	32.0
No Fear	No Fear Extreme Energy 485ml	485	46		10.1		NC	NA
Monster	Monster Rehab Tea + Still Lemonade + Energy 500ml	500	10	10	2.1	2.1	NC	32.0
Relentless	Relentless Energy Drink Cherry 500ml	500		31		7.5	NA	32.0
Monster	Monster Khaos Energy + Juice 500ml	500	34	34	7.8	7.8	32.0	32.0
Lucozade	Lucozade Energy Tropical Fusion Pineapple & Kiwi Flavour 500ml	500		35		4.3	NA	NL
Monster	Monster Ripper Energy + Juice 500ml	500	47	37	10.6	8.4	NC	32.0
Monster	Monster Juiced Energy + Juice 500ml	500		37		8.4	NA	32.0
Monster	Monster Punch Energy 500ml	500		39		9	NA	32.0
Lucozade	Lucozade Energy Pink Lemonade 500ml	500	57	40	14.0	6.8	NL	NL
Lucozade	Lucozade Energy Caribbean Crush 500ml	500	57	40	14.0	6.5	NL	NL
Cott	Emerge Energy Drink Original 500ml	500	42	42	9.8	9.3	NC	30.0
Monster	Monster Energy The Doctor 500ml	500		44		10	NA	32.0
Relentless	Relentless Origin Energy Drink 500ml	500	43	45	10.1	11	NC	32.0
Relentless	Relentless Energy Drink Apple Kiwi 500ml	500		46		11	NA	32.0
KX	KX Energy Stimulation Drink 500ml	500	44	46	10.1	10.5	NC	30.0
Monster	Monster Energy 500ml	500	48	47	11.0	11	32.0	32.0
Boost	Boost Energy Original 500ml	500	47	47	10.9	10.6	NC	30.0
Relentless	Relentless Lemon Ice Energy Drink 500ml	500	48	48	11.6	12	NC	32.0
Sun Mark	Bulldog Power Energy Drink 500ml	500		48		11.3	NA	31.5
Monster	Monster Energy Assault 500ml	500		48		11	NA	32.0
Rockstar	Rockstar Energy Drink 500ml	500		51		12	NA	32.0
Lidl	Lidl Freeway Up Colossus Energy Drink 500ml	500		52		12.8	NA	32.0
Rockstar	Rockstar Xdurance Performance Energy Blueberry, Pomegranate and Acai Flavour 500ml	500	57	57	13.8	14	32.0	32.0

Brand Name	Product Name	Pack Size (ml)	Energy (kcal) per 100ml 2015	Energy (kcal) per 100ml 2017	Sugars (g) per 100ml 2015	Sugars (g) per 100ml 2017	Caffeine (mg) per 100ml 2015	Caffeine (mg) per 100ml 2017
Relentless	Relentless Energy Drink Passion Punch 500ml	500		58		14	NA	32.0
Rockstar	Rockstar Super Sours Energy Drink Bubbleburst 500ml	500	59	59	13.8	14	32.0	32.0
Lucozade	Lucozade Energy Orange 500ml	500	62	62	12.5	12.4	NC	NL
Lucozade	Lucozade Energy The Brazilian Mango Mandarin 500ml	500	62	62	12.4	12	NC	NL
Rockstar	Rockstar Juiced Energy + Juice Mango Orange Passion Fruit Flavour 500ml	500	66	66	15.0	15	32.0	32.0
Rockstar	Rockstar Punched Energy + Guava Tropical Guava Flavour 500ml	500	67	67	15.6	16	32.0	32.0
Sainsbury's	Sainsbury's Blue Bolt Cherry & Blackcurrant Energy Drink 500ml	500	48		10.9		NC	NA
Sainsbury's	Sainsbury's Blue Bolt Energy Drink 500ml	500	49		10.9		NC	NA
Rockstar	Rockstar Punched Energy + Punch Fruit Punch Flavour 500ml	500	64		15.2		32.0	NA
Rockstar	Rockstar Super Sours Energy Drink Green Apple 500ml	500	59		13.8		NC	NA
Rockstar	Rockstar Xdurance Performance Energy Orange Flavour 500ml	500	55		13.5		NC	NA
Lucozade	Lucozade Energy Blackcurrant 500ml	500	67		13.3		NC	NA
PepsiCo	Mountain Dew 500ml	500	48		13.0		18.0	NA
Lucozade	Lucozade Energy Limited Edition Melonade 500ml	500	62		12.4		NC	NA
Lucozade	Lucozade Energy Cherry 500ml	500	61		12.3		NC	NA
Rockstar	Rockstar Recovery Energy + Hydration Non-Carbonated Lemonade with Added Juice 500ml	500	52		12.1		NC	NA
Black	Black Energy Drink Pet 500ml	500	46		10.8		32.0	NA
Relentless	Relentless Tropical Juiced Energy Drink 500ml	500	44		10.7		NC	NA
Relentless	Relentless Berry Juiced Energy Drink 500ml	500	44		10.6		NC	NA
KX	KX Special Edition Jason Plato Energy Stimulation Drink 500ml	500	46		10.5		NC	NA
Powerade	Powerade Energy Sparkling Berry 500ml	500	44		10.5		NC	NA
Powerade	Powerade Energy Sparkling Orange 500ml	500	45		10.5		NC	NA
Relentless	Relentless Orange Energy Drink 500ml	500	43		10.2		NC	NA
Monster	Monster Energy Export 500ml	500	40		9.0		NC	NA
Lucozade	Lucozade Energy Original 500ml	500	70		8.7		NC	NA
Monster	Monster Rehab Tea Orangeade Energy 500ml	500	11		2.1		NC	NA
Monster	Monster Rehab Green Tea Energy 500ml	500	10		1.9		NC	NA
Morrisons	Morrisons Original Source Energy Drink 1 Litre	1000	44	33	10.3	7.3	30.0	30.0
Lucozade	Lucozade Energy Tropical Fusion Pineapple & Kiwi Flavour 1L	1000		35		4.3	NA	NL
Asda	Asda Chosen By You Original Blue Charge 1L	1000		37		7.7	NA	30.0
Lucozade	Lucozade Energy Pink Lemonade 1L	1000	57	40	14.0	6.8	NC	NL
Lucozade	Lucozade Energy Caribbean Crush litre	1000	57	40	13.5	6.5	NC	NL
Lucozade	Lucozade Energy The Brazilian Mango Mandarin 1L	1000		62		12	NA	NL
Lucozade	Lucozade Energy Original 1L	1000	70	70	8.7	8.7	NC	NL
Sainsbury's	Sainsbury's Orange Energy Drink 1L	1000	67		15.9		NC	NA
Lucozade	Lucozade Energy Limited Edition Melonade 1L	1000	62		12.4		NC	NA
Asda	Asda Chosen by You Mixed Fruit Blue Charge Energy Drink 1L	1000	46		10.4		NC	NA

For peer review only

STROBE Statement—checklist of items that should be included in reports of observational studies

	Item No	Recommendation	Page Number Reported
**Title and abstract**	1	(*a*) Indicate the study's design with a commonly used term in the title or the abstract	1-2
		(*b*) Provide in the abstract an informative and balanced summary of what was done and what was found	2
**Introduction**			
Background/rationale	2	Explain the scientific background and rationale for the investigation being reported	3-4
Objectives	3	State specific objectives, including any prespecified hypotheses	2
**Methods**			
Study design	4	Present key elements of study design early in the paper	5-6
Setting	5	Describe the setting, locations, and relevant dates, including periods of recruitment, exposure, follow-up, and data collection	5-6
Participants	6	(*a*) *Cohort study*—Give the eligibility criteria, and the sources and methods of selection of participants. Describe methods of follow-up<br>*Case-control study*—Give the eligibility criteria, and the sources and methods of case ascertainment and control selection. Give the rationale for the choice of cases and controls<br>*Cross-sectional study*—Give the eligibility criteria, and the sources and methods of selection of participants	Not applicable
		(*b*) *Cohort study*—For matched studies, give matching criteria and number of exposed and unexposed<br>*Case-control study*—For matched studies, give matching criteria and the number of controls per case	Not applicable
Variables	7	Clearly define all outcomes, exposures, predictors, potential confounders, and effect modifiers. Give diagnostic criteria, if applicable	5-7
Data sources/ measurement	8*	For each variable of interest, give sources of data and details of methods of assessment (measurement). Describe comparability of assessment methods if there is more than one group	4-7
Bias	9	Describe any efforts to address potential sources of bias	Not applicable
Study size	10	Explain how the study size was arrived at	Not applicable
Quantitative variables	11	Explain how quantitative variables were handled in the analyses. If applicable, describe which groupings were chosen and why	4-7
Statistical methods	12	(*a*) Describe all statistical methods, including those used to control for confounding	7
		(*b*) Describe any methods used to examine subgroups and interactions	7
		(*c*) Explain how missing data were addressed	4-7
		(*d*) *Cohort study*—If applicable, explain how loss to follow-up was addressed<br>*Case-control study*—If applicable, explain how matching of cases and controls was addressed	Not applicable

For peer review only

		Cross-sectional study—If applicable, describe analytical methods taking account of sampling strategy	
		(*e*) Describe any sensitivity analyses	4-7
**Results**			
Participants	13*	(a) Report numbers of individuals at each stage of study—eg numbers potentially eligible, examined for eligibility, confirmed eligible, included in the study, completing follow-up, and analysed	Not applicable
		(b) Give reasons for non-participation at each stage	Not applicable
		(c) Consider use of a flow diagram	Not applicable
Descriptive data	14*	(a) Give characteristics of study participants (eg demographic, clinical, social) and information on exposures and potential confounders	7-8
		(b) Indicate number of participants with missing data for each variable of interest	7-8
		(c) *Cohort study*—Summarise follow-up time (eg, average and total amount)	Not applicable
Outcome data	15*	*Cohort study*—Report numbers of outcome events or summary measures over time	Not applicable
		Case-control study—Report numbers in each exposure category, or summary measures of exposure	7-8
		Cross-sectional study—Report numbers of outcome events or summary measures	Not applicable
Main results	16	(*a*) Give unadjusted estimates and, if applicable, confounder-adjusted estimates and their precision (eg, 95% confidence interval). Make clear which confounders were adjusted for and why they were included	7-11
		(*b*) Report category boundaries when continuous variables were categorized	Fig 1 & 2
		(*c*) If relevant, consider translating estimates of relative risk into absolute risk for a meaningful time period	Not applicable
Other analyses	17	Report other analyses done—eg analyses of subgroups and interactions, and sensitivity analyses	7-8 & Tab 1&2
**Discussion**			
Key results	18	Summarise key results with reference to study objectives	8
Limitations	19	Discuss limitations of the study, taking into account sources of potential bias or imprecision. Discuss both direction and magnitude of any potential bias	14
Interpretation	20	Give a cautious overall interpretation of results considering objectives, limitations, multiplicity of analyses, results from similar studies, and other relevant evidence	8-14
Generalisability	21	Discuss the generalisability (external validity) of the study results	9-14
**Other information**			
Funding	22	Give the source of funding and the role of the funders for the present study and, if applicable, for the original study on which the present article is based	16

*Give information separately for cases and controls in case-control studies and, if applicable, for exposed and unexposed groups in cohort and cross-sectional studies.

For peer review only - http://bmjopen.bmj.com/site/about/guidelines.xhtml

Note: An Explanation and Elaboration article discusses each checklist item and gives methodological background and published examples of transparent reporting. The STROBE checklist is best used in conjunction with this article (freely available on the Web sites of PLoS Medicine at http://www.plosmedicine.org/, Annals of Internal Medicine at http://www.annals.org/, and Epidemiology at http://www.epidem.com/). Information on the STROBE Initiative is available at www.strobe-statement.org.

For peer review only
